# Stabilization of KPNB1 by deubiquitinase USP7 promotes glioblastoma progression through the YBX1-NLGN3 axis

**DOI:** 10.1186/s13046-024-02954-8

**Published:** 2024-01-23

**Authors:** Jie Li, Bin Zhang, Zishan Feng, Dandan An, Zhiyuan Zhou, Chao Wan, Yan Hu, Yajie Sun, Yijun Wang, Xixi Liu, Wenwen Wei, Xiao Yang, Jingshu Meng, Mengjie Che, Yuhan Sheng, Bian Wu, Lu Wen, Fang Huang, Yan Li, Kunyu Yang

**Affiliations:** 1 Cancer Center, Union Hospital, Tongji Medical College, Huazhong University of Science and Technology, Wuhan, 430022 China; 2 Institute of Radiation Oncology, Union Hospital, Tongji Medical College, Huazhong University of Science and Technology, Wuhan, 430022 China; 3 Hubei Key Laboratory of Precision Radiation Oncology, Union Hospital, Tongji Medical College, Huazhong University of Science and Technology, Wuhan, 430022 China

**Keywords:** KPNB1, Nuclear translocation, NLGN3, Glioblastoma, USP7, Deubiquitination

## Abstract

**Background:**

Glioblastoma (GBM) is the most common malignant tumor of the central nervous system. It is an aggressive tumor characterized by rapid proliferation, diffuse tumor morphology, and poor prognosis. Unfortunately, current treatments, such as surgery, radiotherapy, and chemotherapy, are unable to achieve good outcomes. Therefore, there is an urgent need to explore new treatment targets. A detailed mechanistic exploration of the role of the nuclear pore transporter KPNB1 in GBM is lacking. This study demonstrated that KPNB1 regulated GBM progression through a transcription factor YBX1 to promote the expression of post-protrusion membrane protein NLGN3. This regulation was mediated by the deubiquitinating enzyme USP7.

**Methods:**

A tissue microarray was used to measure the expression of KPNB1 and USP7 in glioma tissues. The effects of KPNB1 knockdown on the tumorigenic properties of glioma cells were characterized by colony formation assays, Transwell migration assay, EdU proliferation assays, CCK-8 viability assays, and apoptosis analysis using flow cytometry. Transcriptome sequencing identified NLGN3 as a downstream molecule that is regulated by KPNB1. Mass spectrometry and immunoprecipitation were performed to analyze the potential interaction between KPNB1 and YBX1. Moreover, the nuclear translocation of YBX1 was determined with nuclear-cytoplasmic fractionation and immunofluorescence staining, and chromatin immunoprecipitation assays were conducted to study DNA binding with YBX1. Ubiquitination assays were performed to determine the effects of USP7 on KPNB1 stability. The intracranial orthotopic tumor model was used to detect the efficacy in vivo.

**Results:**

In this study, we found that the nuclear receptor KPNB1 was highly expressed in GBM and could mediate the nuclear translocation of macromolecules to promote GBM progression. Knockdown of KPNB1 inhibited the progression of GBM, both in vitro and in vivo. In addition, we found that KPNB1 could regulate the downstream expression of Neuroligin-3 (NLGN3) by mediating the nuclear import of transcription factor YBX1, which could bind to the NLGN3 promoter. NLGN3 was necessary and sufficient to promote glioma cell growth. Furthermore, we found that deubiquitinase USP7 played a critical role in stabilizing KPNB1 through deubiquitination. Knockdown of USP7 expression or inhibition of its activity could effectively impair GBM progression. In vivo experiments also demonstrated the promoting effects of USP7, KPNB1, and NLGN3 on GBM progression. Overall, our results suggested that KPNB1 stability was enhanced by USP7-mediated deubiquitination, and the overexpression of KPNB1 could promote GBM progression via the nuclear translocation of YBX1 and the subsequent increase in NLGN3 expression.

**Conclusion:**

This study identified a novel and targetable USP7/KPNB1/YBX1/NLGN3 signaling axis in GBM cells.

**Supplementary Information:**

The online version contains supplementary material available at 10.1186/s13046-024-02954-8.

## Introduction

GBM is the most common malignancy of the central nervous system, accounting for about 57% of all gliomas and 48% of all primary malignant central nervous system tumors [1]. GBM is characterized by aggressive and rapid cell proliferation and diffuse tumor morphology [2, 3]. As a high-grade malignant glioma, GBM typically results in a very poor prognosis due to its unique and critical location [4]. Currently, the main treatment modalities of GBM include surgery, radiotherapy, and chemotherapy [5]. The preferred treatment for GBM is maximally safe surgical resection [6]; however, its long-term efficacy is limited and there may be trauma to the surrounding normal brain tissue. The median survival time of patients after surgical resection alone is about 3 months, and postoperative radiotherapy can improve the median survival time to 8 months [7]. The combination of postoperative radiotherapy and temozolomide can further improve the median survival [8]. However, due to the high degree of malignancy of GBM and the fact that glioma cells are prone to develop treatment resistance [9–11], the median survival time of patients remains low, and the existing treatment modalities lead to an average survival time of only 15 months or less [4]. Therefore, it is of great significance to explore the pathogenesis of GBM and develop new treatment methods for glioma patients.

A key factor in cancer progression is the dysregulation of nucleoplasmic protein shuttling, resulting in abnormal protein spatial localization [12, 13]. Karyopherin β1 (KPNB1) is an important nuclear receptor that participates in the nuclear import of many cancer-associated proteins [14–16], and whose expression is related to tumor progression in multiple cancer types. For example, it is reported that KPNB1 can mediate PD-L1 nuclear translocation and promote non-small cell lung cancer cell proliferation [17]. In breast cancer, suppression of KPNB1 inhibited cancer cell proliferation by abrogating the nuclear transport of Her2 [18]. KPNB1 has also been previously studied in glioma; KPNB1 could regulate the proliferation of GBM cells through the Wnt/β-catenin pathway [19]. A subsequent study showed that KPNB1 inhibition could disrupt protein homeostasis, cause protein accumulation in the cytoplasm leading to increased polyubiquitination levels, and trigger the unfolded protein response in GBM cells, ultimately mediating apoptosis via Bcl-2 family members [20]. It has also been shown that KPNB1 can overcome TRAIL resistance by regulating the expression and function of DR5, Mcl-1, and FLIP in GBM cells [21]. These data imply that KPNB1 may be a potential target for future cancer therapy. However, it remains unclear whether KPNB1 is involved in the translocation of oncogenes and regulates the progression of GBM.

Transcription factors (TFs) are key molecular transcriptional regulators, and their roles in disease progression have been extensively reported. The Y-box binding protein (YBX1, YB-1, or nuclease-sensitive element-binding protein 1; NSEP1) is a DNA and RNA binding protein with multiple functions, including the regulation of transcription and translation [22], DNA repair [23], and pre-mRNA splicing [24]. YBX1 is also known as an oncoprotein that is overexpressed in numerous different types of cancer [25, 26]. Initially, YBX1 was found to transcriptionally activate the expressions of ABCB1, an ATP-binding cassette transporter associated with multidrug resistance [27], as well as the growth factor receptor genes *EGFR* and *HER2/ErbB2* in cancer cells [28–30]. In addition, there is strong evidence supporting that the nuclear localization and/or overexpression of YBX1 could predict poor prognosis in patients with more than 20 different tumor types [31, 32]. However, the mechanism of nuclear localization of YBX1 in GBM progression remains to be elucidated. Therefore, this study mainly focused on the effects of nuclear localization of YBX1 on the transcriptional regulation of downstream genes.

Neuroligin 3 (NLGN3) is expressed in neurons and is classified as a postsynaptic adhesion molecule. NLGN3 can interact with presynaptic neurons and plays a key role in synapse formation, differentiation, maturation, and function [33]. Studies have found that NLGN3 secreted by neurons can induce the phosphorylation and activation of several key receptor tyrosine kinases (RTKs) on glioma cells, including focal adhesion kinase activation upstream of PI3K–mTOR [34], thereby promoting glioma progression [35]. Moreover, GBM-derived NLGN3 has an oncogenic function and can induce cancer stem cell (CSC) properties within GBM [36], which is significant because clinical evidence suggests that an increased number of CSCs may contribute to the failure of conventional therapies. However, the mechanisms associated with the acquisition of CSC properties in GBM are not fully understood. Hence, NLGN3 is likely a key neuron-derived factor that regulates glioma growth.

Deubiquitinating enzymes (DUBs) are proteases that process ubiquitin or ubiquitin-like gene products, reverse the modification of proteins by a single ubiquitin(−like) protein, and remodel polyubiquitin(−like) chains on target proteins [37]. Abnormalities in DUBs are associated with various diseases, including inflammatory diseases [38], and drive de novo lipogenesis [39] and cancer [40]. USP7, a member of the ubiquitin-specific processing protease family, is considered a biomarker to predict metastasis and recurrence in various malignancies [41, 42]. USP7 can regulate protein networks by preventing the degradation of p53 and its E3 ligase MDM2 [43]. USP7 can also deubiquitinate and stabilize EZH2 in prostate cancer cells [44]. To the best of our knowledge, little is known about the function of USP7 in GBM genesis; therefore, it is of great interest to elucidate novel substrates of USP7.

The current study aimed to elucidate the mechanistic role of KPNB1 in GBM. The results showed that KPNB1 was upregulated in GBM cell lines, an increase that was mediated by the deubiquitinating enzyme USP7. Moreover, this increase in KPNB1 expression promoted the nuclear translocation of YBX1, a transcription factor [45] that is enriched in the transcriptional start region of NLGN3, thus promoting the expression of the key oncogenic factor NLGN3. Therefore, this study elucidated a previously unknown mechanism of GBM development and provided a novel therapeutic target for GBM treatment.

## Material and methods

### Cell culture

U251MG and U87MG glioma cell lines were obtained from the Cell Bank of Shanghai Biological Institution (Shanghai, China). Glioma cell lines were maintained in DMEM containing 10% FBS and antibiotics at 37 °C with 5% CO_2_ concentration. All cell lines were mycoplasma-free and routinely tested by PCR amplification.

### Tissue microarray and immunohistochemistry (IHC)

The tissue microarray (cat. no. N783701, Bioaitech, CN) and IHC were employed to assess the levels of KPNB1 (#10077–1-AP, Proteintech; 1:1000 dilution) and USP7 (#66514–1-Ig, Proteintech; 1:2500 dilution) in glioma tissue. The IHC score was evaluated as previously reported [46].

### Plasmid construction and reagents

Plasmids for the overexpression of KPNB1, USP7, YBX1, and NLGN3 were obtained from WZ Bioscience (Shandong, China). Flag-KPNB1 was cloned into the CMV-MCS-3xFlag-SV40-neomycin vector. Myc-USP7 was cloned into the pEnter vector. His-YBX1 was cloned into the pEnter vector with C-terminal Flag and His tags. HA-NLGN3 was cloned into the pCMV-N-HA vector. Antibodies against KPNB1 (#10077–1-AP), YBX1 (#20339–1-AP), NLGN3 (#AC039), USP7 (#66514–1-Ig, Proteintech, 1:5000 dilution), GAPDH (#60004–1-Ig, Proteintech, 1:10000 dilution), HA-tag (#51064–2-AP, Proteintech, 1:5000 dilution), Flag-tag (#66008–4-Ig, Proteintech, 1:5000 dilution), P5091, and MG132 (#S2619) were obtained from Selleck (Shanghai, China).

### RNA interference

Short hairpin RNAs (shRNAs) were obtained from GeneChem (Shanghai, China), and siRNA was purchased from RiboBio (Guangzhou, China). Lipofectamine 2000 (Thermo Fisher Scientific, China) and Opti-MEM media (Invitrogen, USA) were used for the transfection studies, with 50 nM of each siRNA, as previously described [47]. At least 48 hours later, cells were harvested and analyzed. The sequences of the shRNAs and siRNAs are provided in Supplementary Tables S1 and S2.

### Immunofluorescence staining

After 48 h of transfection treatment, U251MG cells were trypsinized, re-seeded into 24-well plates supplemented with sterile slides, and cultured for a further 24 hours. The next day, 4% paraformaldehyde was added to the 24-well plate to fix the cells on the slides. Immunofluorescence staining was then performed as described previously [48]. After staining was completed, fluorescence images were obtained using a fluorescent microscope (Olympus IX71, Japan) and a confocal laser-scanning microscope (LSM780, ZEISS, Germany).

### Western blotting and co-immunoprecipitation (co-IP)

Western blotting was performed to measure protein levels in glioma cell homogenates. Cells were lysed using RIPA buffer containing protease inhibitors. Lysates were incubated on ice for 20 min and then centrifuged at 10,000 g for 10 min at 4 °C. Protein concentrations were standardized across all samples. Then, samples were mixed with 5× loading buffer (1:4), boiled for 10 min, and run on SDS-PAGE gels. Proteins were transferred to PVDF membranes and blocked with 5% bovine serum albumin (BSA) in TBST for 1 h. Antibodies were incubated with membranes at 4 °C overnight. Horseradish peroxidase (HRP)-conjugated secondary anti-rabbit antibody (BioRad) was then added for 1 h (1:1000). Proteins were visualized using Clarity ECL Western Substrate (BioRad) and quantified and analyzed using ImageJ. For co-immunoprecipitation (IP), collected cells were suspended in 1 mL RIPA protein lysate containing 1% protease inhibitors on ice for at least 30 min. The supernatant was collected and co-cultured with Protein A and G Agarose beads (#G1718, Santa Cruz, America) and primary antibodies or IgG at 4 °C for 24 h. Then, the beads were washed with NETN buffer 6 times, and 50 μL of sample loading buffer was added, followed by boiling for 15 min. These samples were then subjected to Western blotting analysis. The antibodies used are as follows: KPNB1 (#10077–1-AP, Proteintech, 1:200 dilution), USP7 (#66514–1-Ig, Proteintech, 1:500 dilution), and YBX1 (#20339–1-AP, Proteintech, 1:200 dilution).

### Deubiquitination assay

The deubiquitination assay was performed using IP analysis. U251MG cells were co-transfected with sh-USP7 and HA-UB or Myc-USP7, sh-USP7, and HA-UB and allowed to grow for 60 hours. Then, the proteasome inhibitor MG132 was added for another 12 hours. The cells were collected and lysed in EBC buffer (50-mM Tris-HCl pH = 7.6–8.0, 0.5% NP-40, 1 mM EDTA, 1 mM Na3VO4, 50-mM NaF, and 1 mM β-mercaptoethanol) supplemented with protease inhibitors. After centrifugation at 14,000 rpm for 10 min, the supernatant was collected and incubated with agarose beads at 4 °C overnight with rotation. After extensive washing, 1× loading buffer was added, and the proteins were separated with SDS-PAGE followed by Western blot analysis.

### Proximity ligation assay (PLA)

The U251MG cells were fixed with the blocking solution following the manufacturer’s protocol (Duolink in situ fluorescence; Sigma). Then, the primary antibodies against KPNB1 (67,597; Proteintech; 1:200 dilution) and YBX1 (20339–1-AP; Proteintech; 1:300 dilution) or IgG (Rabbit) (3900; Cell Signaling Technology; 1:5000 dilution) and IgG (Mouse) (53,484; Cell Signaling Technology; 1:5000 dilution) were added to the cells and incubated for 2 h at 37 °C. Then, the cells were washed with 1× wash buffer and incubated with PLA probe for 1 h at 37 °C. The ligation–ligase was added to cells at 37 °C. After 30 min, the cells were incubated with amplification–polymerase solution for 100 min. The Duolink In Situ Mounting Medium with DAPI was added to cells, and images were taken under a confocal microscope. PLA and DAPI signals were counted under a fluorescence microscope, and a high-resolution intercellular visualization was performed using a confocal microscope (Leica Microsystems, Wetzlar, Germany). Detailed operation is referred to this article [47].

### Nuclear-cytoplasmic separation

Two transfected cell lines (sh-control and sh-KPNB1) were collected in a centrifuge tube, to which cytoplasmic protein extraction reagent A with pre-added phenylmethanesulfonyl fluoride (PMSF) was added. After shaking and mixing, cytoplasmic protein extraction reagent B was added to the samples and incubated on ice for 10 min. After shaking and mixing, the samples were centrifuged at 12,000 g and 4 °C for 5 min. The supernatants were collected in precooled centrifuge tubes and the cytoplasmic proteins were obtained, while the pellets were collected for nuclear protein extraction. A nuclear protein extraction reagent with pre-added PMSF was added to the pellet. The samples were then incubated in the ice bath for 2 min, followed by shaking for 20 s; this process was repeated for a total of 30 min. Then, the samples were centrifuged at 1000 g and 4 °C for 10 min, and the supernatants were obtained as nuclear protein extracts. The concentrations of cytoplasmic and nuclear proteins were measured using a BCA assay and analyzed using Western blot.

### Clonogenic survival assay

Cells were plated in 6-well plates at a density of 1000 cells per well and cultured for 10 days. Colonies were fixed and stained with 4% formaldehyde in PBS containing 0.02% crystal violet for 30 min, then washed with tap water. The number of colonies was counted and recorded.

### In vitro invasion assay

Transwell chambers (8.0 μm Pore Size, CORNING) were coated with 50 μL Matrigel. Cells were resuspended in serum-free media and added to the upper chambers (5 × 10^4^ cells/200 μL/chamber) of the Transwell plates. The lower chambers were filled with 500 μL of serum-containing media [49, 50]. After 48 h, non-invading cells on the upper surface of each Transwell membrane were removed using a cotton swab, and cells on the lower surface were stained with 1% crystal violet. Five randomly chosen areas were photographed under a microscope, and the number of stained cells was counted.

### CCK-8 assay

3000 cells were plated in 96-well plates and cultured for 5 days with 200 μL of DMEM containing 10% FBS. The CCK-8 assay was performed according to the manufacturer’s instructions. In brief, 20 μL of CCK-8 reagent (#C0037, Beyotime) was added to each well 1 hour before the end of the incubation period. The optical absorbance at 450 nm in each well was measured using a microplate reader.

### Reverse transcription-quantitative polymerase chain reaction (RT-qPCR)

Total RNA was extracted from cells using the OMEGA Total RNA extraction kit according to the manufacturer’s protocol. RNA concentrations were quantified using a Nanodrop spectrophotometer. Reverse transcription was performed to synthesize cDNA using the Novy HiScript® III RT SuperMix for qPCR retrovirus kit according to the manufacturer’s instructions. RT-qPCR was performed using a TB Green™ Fast qPCR Mix kit (TAKARA, RR430A, JPN). The data were presented as the average of three technical replicates from at least five independent experiments (biological replicates). The primer sequences for the genes are provided in Supplementary Table S3.

### EdU incorporation assay

Treated cells were seeded in 96-well plates at 5000 cells per well. The next day, the supernatant was discarded and 10 μM EdU was added to the complete medium, which was provided to cells at 200 μL per well. After 2 h of incubation at 37 °C, cells were fixed for 1 h with 4% paraformaldehyde in PBS and stained using the BeyoClick-TM EdU-488 kit and protocol (Beyotime). The percentage of EdU-labeled cells was determined by confocal microscopy at 200× magnification, and the cell proliferation index was calculated.

### Chromatin immunoprecipitation (ChIP) assay

The binding sites of YBX1 and NLGN3 were determined using a ChIP assay. For this purpose, formaldehyde was added to the cells to facilitate cross-linking of the target proteins with genomic DNA. Then, cells were digested to obtain lysates, which were then sonicated to achieve genomic DNA of 200–1000 bp fragments. The target proteins and the DNA fragments bound to them were co-immunoprecipitated, purified, and amplified using PCR. The primer sequences for ChIP-qPCR are provided in Supplementary Table S4.

### Liquid chromatography-tandem mass spectrometry (LC-MS/MS) analysis

293 T cells transfected with a Flag-KPNB1-expressing plasmid were used for the identification of novel KPNB1-binding proteins. KPNB1 protein was immunoprecipitated using an anti-KPNB1 antibody and protein A + G agarose beads (#P2012, Beyotime, China) at 4 °C. LC-MS/ MS analysis was performed using a Thermo Scientific Ultimate 3000 RSLC system combined with a Q Exactive Plus high-resolution mass spectrometer by SpecAlly Life Technology Co., Ltd., Wuhan, China. The data were retrieved using MaxQuant (v1.6.6) software and the Andromeda algorithm. The UniProt human proteome database was used as a reference database. Proteins and peptides were filtered using a false discovery rate (FDR) of 1%.

### Flow cytometry

The two stable glioma cell lines were digested with trypsin in their logarithmic growth phase and collected in a flow cytometry tube with a pre-added culture medium. After centrifugation at 300 g for 5 min, the supernatants were discarded, and the pellets were washed with PBS and re-suspended in 300 μL of binding buffer. Then, 5 μL of Annexin V (FITC) was added and samples were incubated in the dark for 10 min. Following this, 5 μL of propidium iodide (PI) was added, mixed, and incubated in the dark for a further 5 min. Flow cytometry was performed, and the corresponding channels were detected and observed within 1 hour.

### Transcriptome sequencing (RNA-seq)

Total RNA was extracted using TRIzol reagent (#R401–01 RNA isolator Total RNA Extraction Reagent, Vazyme, Nanjing, China), and transcriptome sequencing was performed by NOVOGENE (Beijing, China). The library preparations were sequenced on an Illumina NovaSeq platform, and 150 bp paired-end reads were generated. The RNA-seq data is provided in Supplementary Table S5.

### In vivo xenografts

All experimental procedures on animals were performed according to the guidelines and regulations approved by the Ethics Committee of Tongji Medical College, Huazhong University of Science and Technology. Female BALB/c nude mice (4–5 weeks old) were purchased from Shulaibao Biotech (Wuhan, China) and acclimatized for 1 week under specific pathogen-free conditions in standard cages. To establish the intracranial disease model, GBM cells expressing luciferase (U251MG-luc) (1 × 10^6^ cells) were implanted intracranially into nude mice using a previously described guide-screw system [51]. Treated U251MG cells were injected into the brains of BALB/c nude mice (4 to 5 weeks old). The origin of the anterior fontanelle was 2 mm to the left and 1.5 mm to the back, and the depth of the needle was 2.5 mm. Tumor growth and development were visualized and quantified using an IVIS Spectrum in vivo imaging system.

## Statistical analysis

All experiments were performed in at least three biological replicates. The data were statistically analyzed and plotted using GraphPad Prism 8.0. Western blot and DNA gel electrophoresis data were analyzed using Image J. Categorical data were analyzed using the chi-square test. Continuous data involving two groups were analyzed using an unpaired t-test, and data involving three or more groups of samples were analyzed using one-way ANOVA with Tukey’s post hoc test. All the graphs show the mean ± SD of triplicate experiments. A *P*-value of less than 0.05 was considered statistically significant.

## Results

### KPNB1 was abnormally expressed in glioma tissues and predicted poor prognosis

**Fig. 1:**
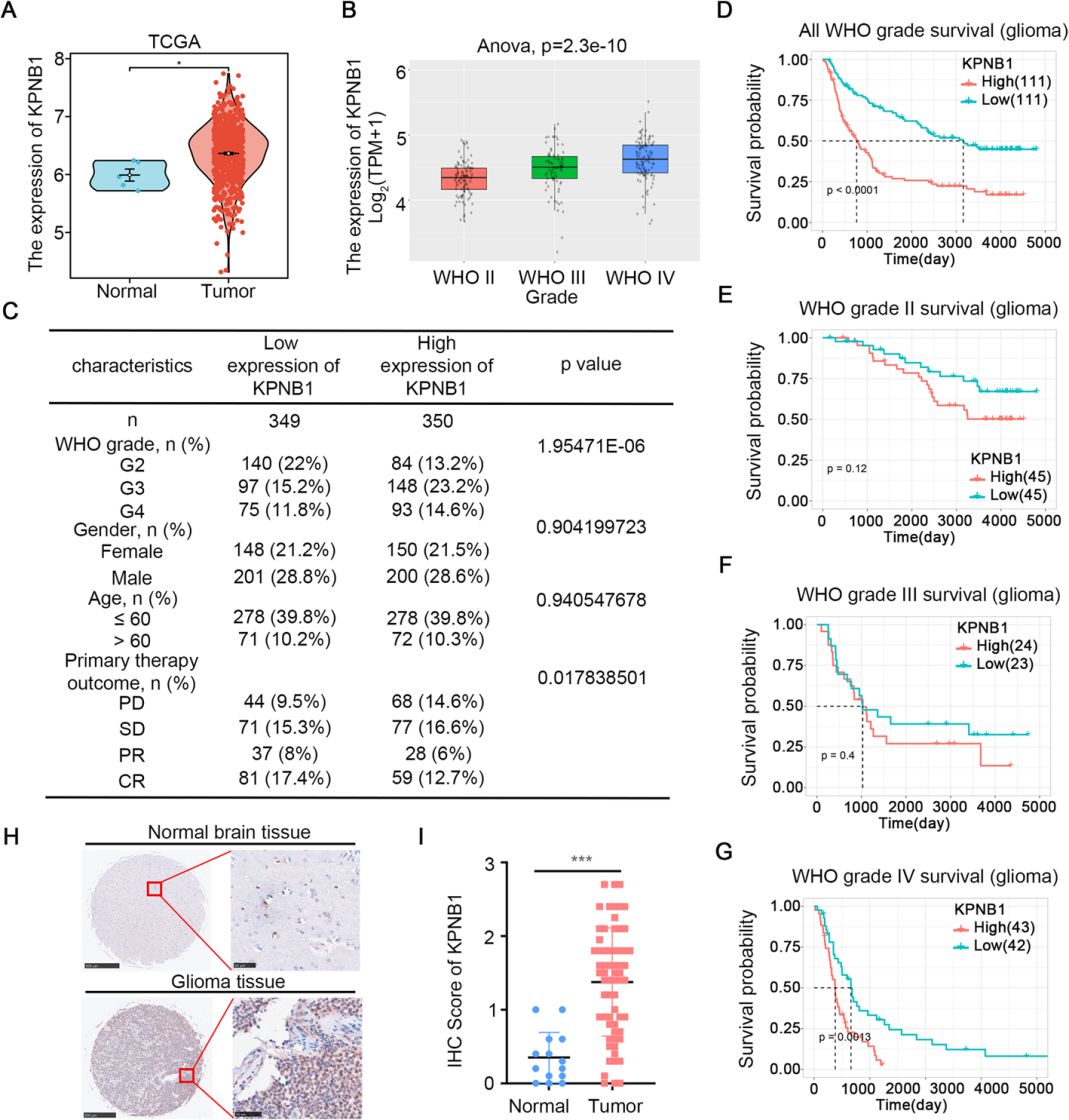
KPNB1 was abnormally expressed in glioma tissues and predicted poor prognosis. A. TCGA database analysis showed that the expression of KPNB1 was higher in glioma tissues than in normal tissues. B. CGGA database analysis showed that the expression of KPNB1 increased with increasing WHO glioma grade. C. The baseline characteristics, WHO grade, initial treatment, and prognosis of glioma patients were analyzed using a chi-square test for categorical variables and a Student’s *t*-test for continuous variables. D-G. A Kaplan-Meier plotter was used to analyze the effects of KPNB1 on the overall survival of glioma patients. The log-rank test was used to detect statistically significant differences. H-I. IHC Images (H) and dot plots (I) of KPNB1 staining using glioma and normal tissue sections (***P < 0.001, unpaired *t*-test)

To verify the relationship between KPNB1 expression and glioma grade and prognosis, the mRNA expression levels of KPNB1 in the TCGA database were analyzed. The results showed that KPNB1 expression was increased in glioma tissues compared with normal brain tissues (Fig. 1A). Further analysis using the CGGA database found that KPNB1 levels were positively associated with the WHO grade of the glioma, with higher KPNB1 expression in WHO IV gliomas than those graded as WHO II or WHO III (Fig. 1B). Analyzing the clinical characteristics of glioma patients in the TCGA database showed that KPNB1 expression was not related to age and gender but associated with the initial glioma treatment efficacy. Patients with high KPNB1 expression had worse initial treatment efficacy and lower PR and CR (Fig. 1C). Moreover, additional analysis of the TCGA database showed that KPNB1 expression was negatively correlated with the overall survival rate of patients, a relationship that became more statistically significant with increasing WHO glioma grade (Fig. 1D-G).

To confirm the database analyses, KPNB1 protein levels were measured in a tissue microarray containing specimens from a cohort of glioma patients (*n* = 64) and non-tumor brain tissue specimens (*n* = 14). The findings demonstrated that KPNB1 was upregulated in glioma tissues compared to non-tumor brain tissues (Fig. 1H and I). Therefore, our data demonstrated that KPNB1 was upregulated in glioma tissues compared to healthy tissues, with high KPNB1 expression levels associated with poor prognosis among glioma patients.

### Abnormally high expression of KPNB1 promoted glioma progression in vitro and in vivo

**Fig. 2:**
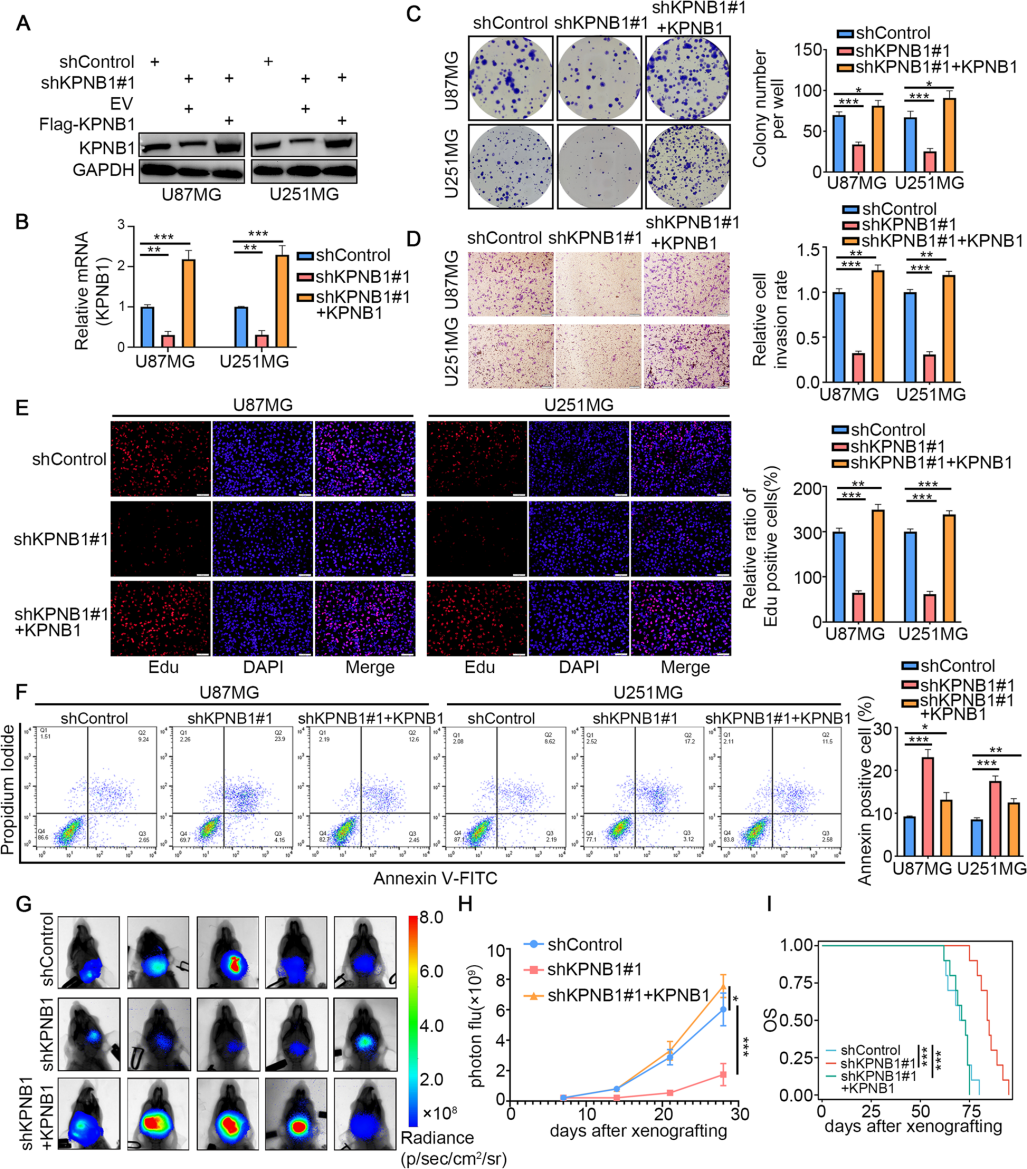
KPNB1 knockdown inhibited glioblastoma growth, which could be rescued by overexpression of KPNB1. A. Western blot analysis showing KPNB1 expression in U87MG and U251MG cells infected with lentivirus vectors expressing KPNB1-specific shRNAs or shRNAs + KPNB1 plasmid. B. RT-qPCR results showing KPNB1 expression in U87MG and U251MG cells. Data presented as the mean ± SD of three independent experiments, ***P < 0.001, one-way ANOVA. U87MG and U251MG cells infected with lentivirus vectors expressing KPNB1 specific shRNAs or shRNAs + KPNB1 plasmid after puromycin selection were harvested for colony formation assay (C), Transwell invasion assay (D), and fluorescein isothiocyanate (FITC)/PI flow cytometry (F). Scale bars: 100 μm. Each bar represents the mean ± SD of three independent experiments. *P < 0.05; **P < 0.01; ***P < 0.001, one-way ANOVA. U251MG cells were transfected with the indicated constructs. E. EdU assay of U87MG and U251MG cells infected with lentivirus vectors expressing KPNB1-specific shRNAs or shRNAs + KPNB1 plasmid after puromycin selection. EdU-positive nuclei (red) and 1× DAPI-stained nuclei of all cells (blue) were visualized by fluorescence microscopy. Quantification of the EdU assay on the right. Error bars represent mean ± SD one-way ANOVA, Tukey’s multiple comparisons test; *n* = 3 independent experiments, *P < 0.05; **P < 0.01; ***P < 0.001. G. After puromycin selection, cells were administered to nude mice by intracranial injection to establish a xenograft model. Representative bioluminescence imaging of a tumor at day 28 is shown. H. Quantitative assessments of tumor growth following implantation. Data expressed as mean ± SD, *n* = 10 per group. *P < 0.05; **P < 0.01; ***P < 0.001, one-way ANOVA. I. Mice were sacrificed at the ethical endpoint, and survival curves were plotted in Kaplan–Meier graphs, with differences evaluated using the log-rank test (*P* < 0.001 for control vs. shKPNB1; *P* < 0.001 for shKPNB1 vs. shKPNB1+ KPNB1)

Since KPNB1 is highly expressed in gliomas and associated with poor prognosis, we hypothesized that it may have a tumorigenic role in gliomas. To this point, we performed KPNB1 knockdown experiments in U251MG and U87MG cells using two different gene-specific shRNAs (Supplementary Fig. S1A and S1B). KPNB1 silencing profoundly decreased the proliferation, invasion, and migration of GBM cell lines, as determined by CCK8, colony formation, Transwell, and EdU assays (Fig. 2C, Supplementary Fig. S1C-S1E). Conversely, we overexpressed KPNB1 by ectopically transfecting U87MG and U251MG cells with KPNB1 expression plasmids (Supplementary Fig. S1F and S1G) and achieved the opposite effects to KPNB1 knockdown, showing increased proliferation, invasion, and migration of GBM cells (Supplementary Fig. S1H and S1J). Moreover, we showed that rescue of KPNB1 expression in cells with KPNB1 silencing (Fig. 2A and B) reversed the suppressive effects on cell proliferation and invasion induced by KPNB1 knockdown in vitro (Fig. 2C-E). Previous studies have shown that lentivirus-mediated expression of shKPNB1 in GBM cells can induce the cleavage of caspase-3 and PARP, leading to apoptosis [20]. As expected, increased apoptosis was observed following KPNB1 knockdown in U87MG and U251MG cells, which was rescued by KPNB1 overexpression (Fig. 2F). We further analyzed the tumorigenic effects of KPNB1 in vivo using a model of intracranial orthotopic tumors in nude mice. We found that the knockdown of KPNB1 inhibited tumor growth and prolonged the overall survival of mice. However, overexpression of KPNB1 could rescue this inhibitory effect and promote intracranial GBM growth, resulting in shortened overall survival of these mice (Fig. 2G-I). The standard treatment for GBM consists of surgical resection followed by palliative radiotherapy and chemotherapy [52]. Although temozolomide (TMZ) treatment could prolong a subset of effective patients by up to 2.5 months, GBM usually exhibits a unique phenotype of resistance to DNA damage and is inevitably resistant to the effects of TMZ [53]. Since KPNB1 had a regulatory effect on the malignant phenotype of GBM cells, it was speculated that KPNB1 might regulate TMZ sensitivity. The results showed that as compared to the TMZ group, the proliferation ability of GBM cells treated with KPNB1 knockdown combined with TMZ was further inhibited, and the number of apoptotic cells was further increased (Supplementary Fig. S1K and S1L). Taken together, these results suggested a tumor-promoting function of KPNB1 in GBM. The knockdown of KPNB1 not only limited GBM progression but also increased sensitivity to TMZ treatment. This provided a rationale for the clinical translation of KPNB1 as a therapeutic target.

### Transcriptome sequencing revealed that KPNB1 reduced the expression of NLGN3 in GBM

**Fig. 3:**
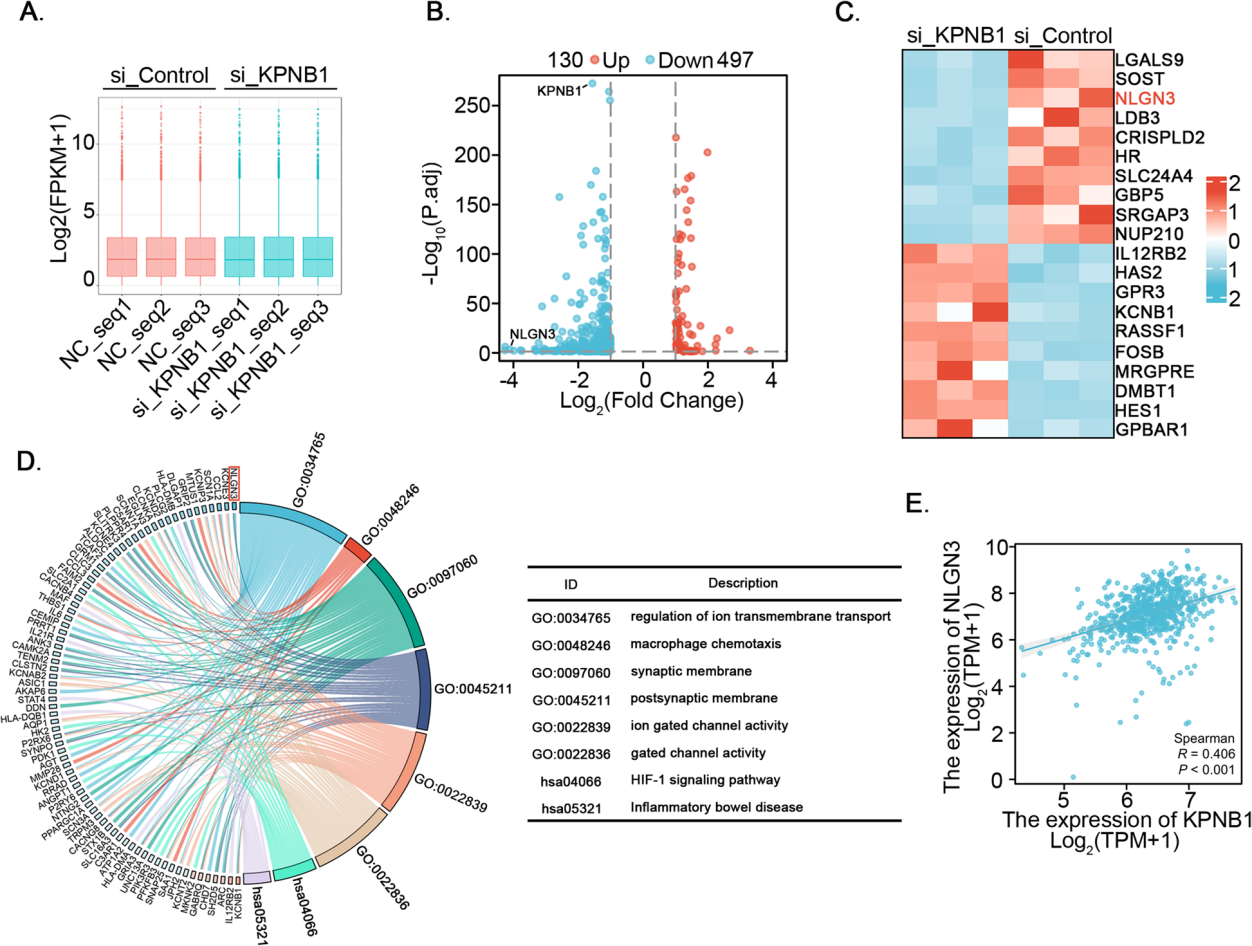
Transcriptome sequencing revealed that KPNB1 knockdown reduced the expression of NLGN3 in glioblastoma. A. U251MG cells infected by siControl or siKPNB1 were harvested for transcriptomic RNA sequencing. Boxplots show the quality control of RNA sequencing. B-C. Volcano plot (B) and heat map (C) show that NLGN3 was significantly down-regulated in KPNB1-silenced U251MG cells. D. GO functional enrichment analysis was performed and KPNB1 was associated with multiple synaptic activities in U251MG cells. E. TCGA (*n* = 701) database correlation analysis showed that there was a positive correlation between the mRNA expression levels of NLGN3 and KPNB1

To further elucidate the mechanism by which KPNB1 regulated GBM progression, RNA sequencing analysis was performed following small interfering RNA (siRNA) knockdown of KPNB1 in U251MG cells. Boxplots (Fig. 3A) were used to visualize the quality control of RNA sequencing. We analyzed and identified 130 upregulated and 497 downregulated DEGs (Fig. 3B). The volcano plot (Fig. 3B) and heat map (Fig. 3C) indicated that knockdown of KPNB1 resulted in a marked decrease in the expression of neuroligin 3 (NLGN3). NLGN3 is a postsynaptic adhesion molecule that can interact with presynaptic neuronal proteins and play a key role in the formation, differentiation, maturation, and function of synapses [54]. Notably, NLGN3 has been implicated in GBM, and a study by Venkatesh et al. published in *Cell* identified NLGN3 as a mitogen that could promote the growth of high-grade gliomas [34]. GO functional enrichment analysis showed that KPNB1 was associated with multiple synaptic activities in U251MG cells (Fig. 3D). NLGN3 protein has been characterized as a synaptic organizer [55], which is consistent with the hypothesis that KPNB1 has a role related to the function of NLGN3 and is involved in the regulation of GBM progression. Moreover, TCGA (*n* = 701) database correlation analysis showed that there was a positive correlation between the mRNA expression levels of NLGN3 and KPNB1 (Fig. 3E). These results suggested that KPNB1 might positively regulate NLGN3 expression in GBM.

### KPNB1 regulated GBM progression through NLGN3

**Fig. 4:**
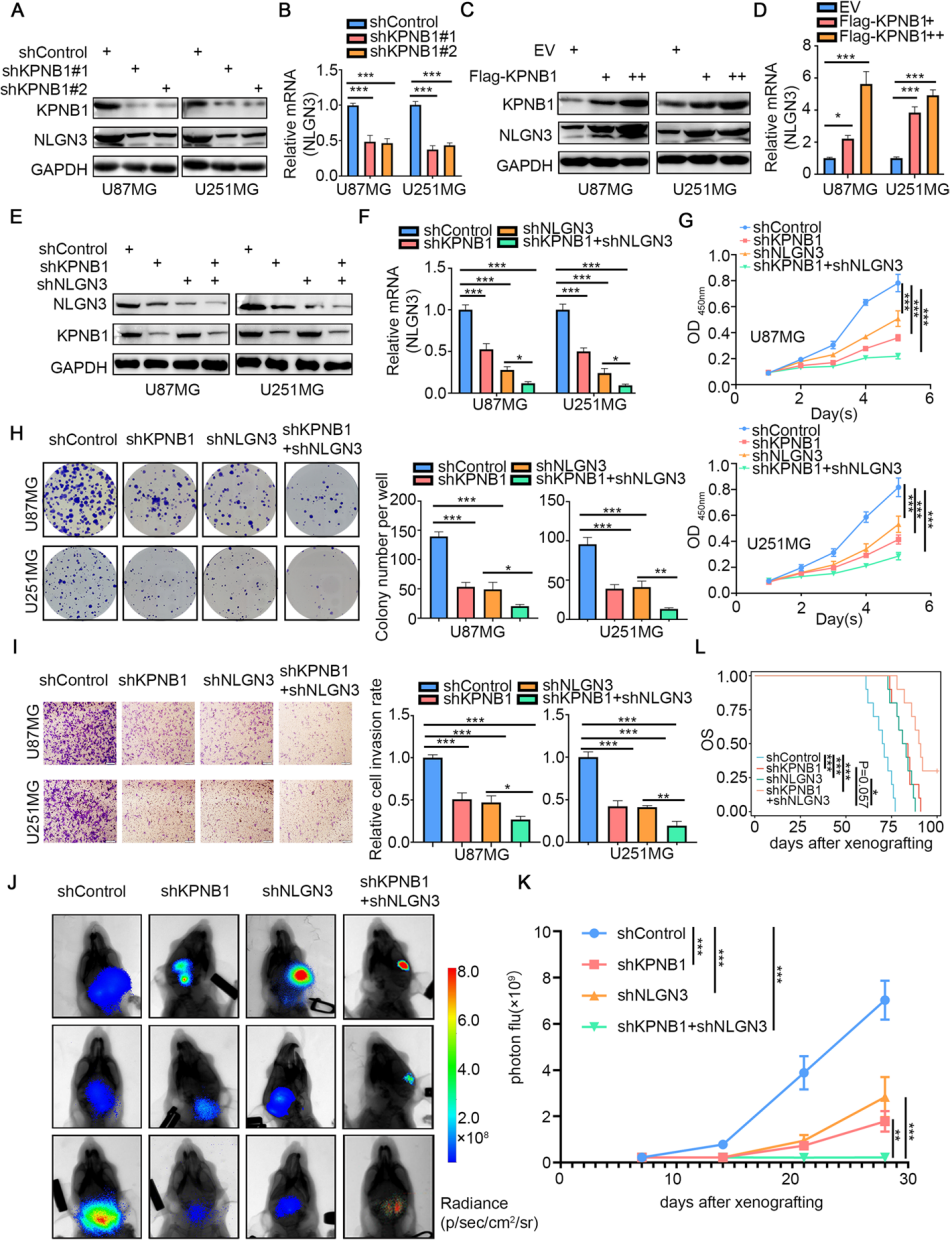
KPNB1 regulated glioblastoma progression through NLGN3. A. Western blot analysis showing KPNB1 and NLGN3 expression in U87MG and U251MG cells infected with lentivirus vectors expressing KPNB1-specific shRNAs. B. RT-qPCR results showing NLGN3 expression in U87MG and U251MG cells. Data presented as the mean ± SD of three independent experiments, ***P < 0.001, one-way ANOVA.C-D. U87MG and U251MG cells were infected with lentivirus vectors expressing KPNB1. C. Western blot analysis showing KPNB1 and NLGN3 expression. D. RT-qPCR results showing NLGN3 expression. Data presented as the mean ± SD of three independent experiments, ***P < 0.001, one-way ANOVA. E-L. U87MG and U251MG cells were infected with lentivirus vectors expressing shKPNB1, shNLGN3, or shKPNB1 combined with shNLGN3. Cells were collected for Western blot analysis (E), RT-qPCR (F), CCK8 assay (G), colony formation assay (H), and transwell invasion assay (I). J. After puromycin selection, cells were administered to nude mice by intracranial injection to establish the xenograft model. Representative bioluminescence imaging of a tumor at day 28 is shown. K. Quantitative assessments of tumor growth following implantation. Data expressed as mean + SD, *n* = 10 per group. *P < 0.05; **P < 0.01; ***P < 0.001, one-way ANOVA. L. Mice were sacrificed at ethical endpoint, survival curves were plotted in Kaplan–Meier graphs, and differences were evaluated using the log-rank test (*P* < 0.001 for control vs. shKPNB1; *P* < 0.001 for control vs. shNLGN3; *P* < 0.001 for control vs. shKPNB1 + shNLGN3; *P* = 0.057 for shKPNB1 vs. shKPNB1 + shNLGN3; *P* = 0.012 for shNLGN3 vs. shKPNB1 + shNLGN3)

Given the marked downregulation of NLGN3 following KPNB1 knockdown, and the previously reported roles of NLGN3 in various cancers, especially in nervous system tumors [36, 56–58], we hypothesized that KPNB1 regulated GBM progression through NLGN3. To test this hypothesis, we knocked down KPNB1 in two cell lines (U87MG and U251MG) and found that the protein and mRNA levels of NLGN3 were reduced in response to KPNB1 knockdown (Fig. 4A and B). The overexpression of KPNB1 produced the opposite effect, with increased NLGN3 expression (Fig. 4C and D). Next, we used lentiviral vectors to construct U87MG and U251MG cell lines as follows: KPNB1 knockdown alone, NLGN3 knockdown alone, and combined KPNB1-NLGN3 knockdown (Fig. 4E and F). CCK8, colony formation, and Transwell invasion assays showed that knockdown of each of KPNB1 and NLGN3 alone inhibited the proliferation, invasion, and migration of GBM cell lines, and these inhibitory effects were further enhanced in the combined knockdown group (Fig. 4G-I). In vivo experiments further demonstrated that intracranial tumor growth was significantly inhibited in the combined KPNB1-NLGN3 knockdown group, with prolonged survival time (Fig. 4J-L). We also showed that co-overexpression of KPNB1 and knockdown of NLGN3 attenuated the downregulation of NLGN3 expression caused by NLGN3 silencing alone (Supplementary Fig. S2A and S2B), as well as the inhibitory effect of NLGN3 silencing alone on GBM cell lines (Supplementary Fig. S2C-E). These data suggested that both KPNB1 and NLGN3 played important roles in regulating GBM progression, and the effects of KPNB1 on GBM progression were primarily mediated via NLGN3.

### KPNB1 interacted with YBX1 and regulated its nuclear localization in GBM

**Fig. 5:**
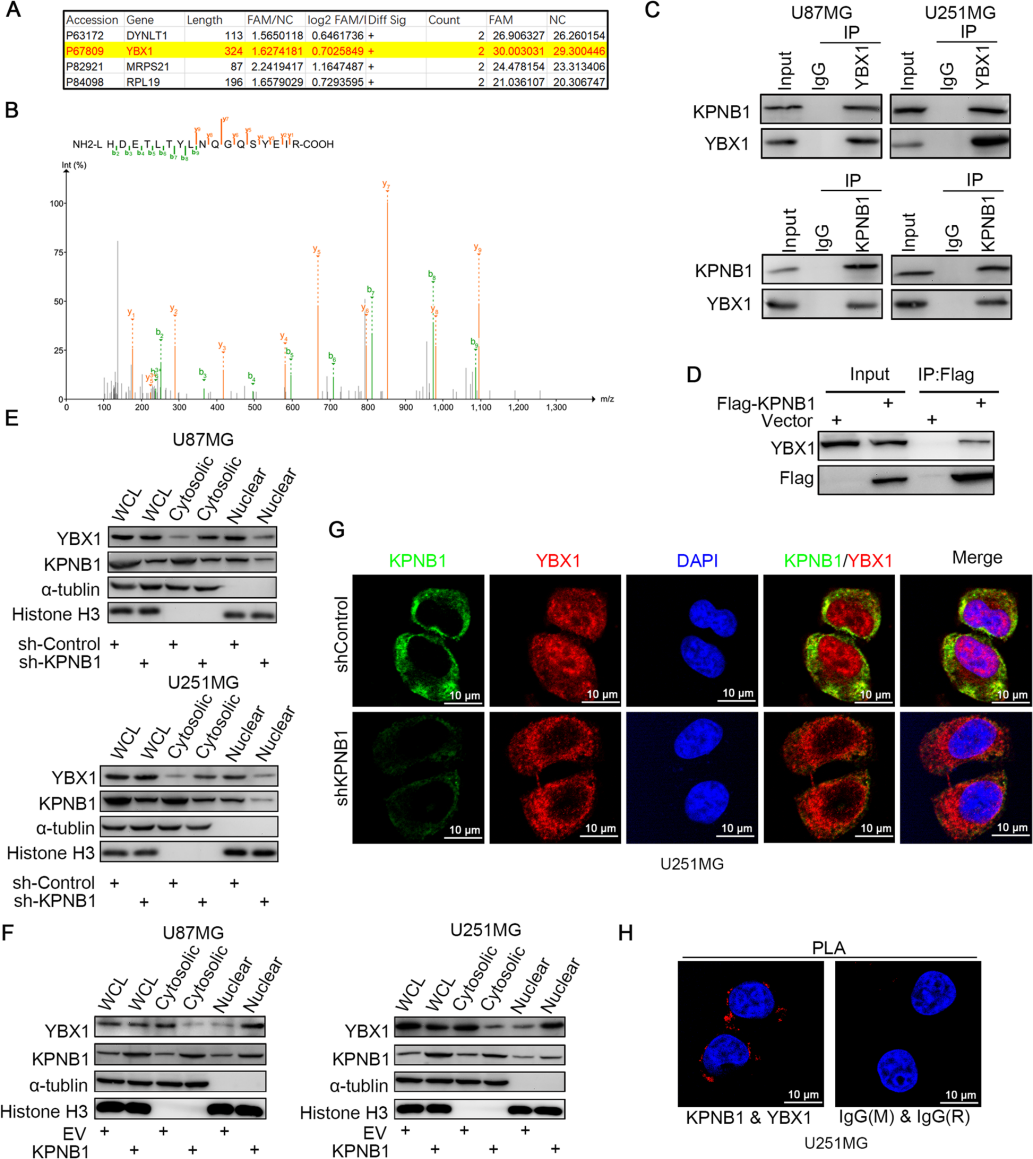
KPNB1 interacted with YBX1 and regulated its nuclear localization in glioblastoma. A. Mass spectrometry was used to identify the proteins obtained in U251MG cells. A subset of the identified peptides is displayed. B. Mass spectrometric sequencing of YBX1. C. Western blot analysis of reciprocal co-IP in U87MG and U251MG cells, demonstrating that endogenously expressed KPNB1 bound to YBX1. D. KPNB1-Flag expression plasmid was transfected into 293 T cells. Reciprocal co-IP demonstrating the interaction of exogenously expressed KPNB1 with endogenously expressed YBX1. E-F. Western blot analysis was used to detect the protein expression of YBX1 in the cytoplasm and nucleus of U87MG and U251MG infected with sh-Control or shKPNB1 (E) and EV or KPNB1 plasmids (F). G. U251MG cells were classified as treatment (shKPNB1) or control. Cells were incubated with rabbit anti-KPNB1 antibody and mouse anti-YBX1 antibody for immunofluorescence to determine the nuclear translocation of YBX1. H. PLA was used to verify the interaction between KPNB1 and YBX1 in U251MG cells. The size of the scale bar in microscopy images was 20 μm

As KPNB1 is a member of the karyopherin β family, it mediates the transport of proteins from the cytoplasm to the nucleus [59]. Since KPNB1 has not been reported to function as a transcription factor, we speculated that KPNB1 regulated NLGN3 indirectly. Therefore, we performed mass spectrometry to identify the binding partners of KPNB1 (Fig. 5A). YBX1 was identified by screening proteins associated with NLGN3 transcript levels from the mass spectrometry results; the amino acid sequence of YBX1 as determined by mass spectrometry is shown in Fig. 5B. We also found by reciprocal co-IP in U87MG and U251MG cells that endogenously expressed KPNB1 bound to YBX1 (Fig. 5C). The KPNB1-Flag expression plasmid was transfected into 293 T cells, and reciprocal co-IP assay results indicated that exogenously expressed KPNB1 interacted with endogenously expressed YBX1 in 293 T cells (Fig. 5D). To investigate the regulatory relationship between KPNB1 and YBX1, we transfected plasmids encoding shRNAs against KPNB1, YBX1, or both into the two cell lines. The results showed that the protein expression of YBX1 was not changed after KPNB1 knockdown (Supplementary Fig. S3A-C); however, the cell proliferation was significantly inhibited in the combined knockdown group compared with the corresponding single knockdown groups (Supplementary Fig. S3D and S3E). Therefore, we hypothesized that KPNB1 played a carcinogenic role by regulating the cytoplasmic/nuclear localization of YBX1. To test this, KPNB1 was knocked down in the two cell lines (U87MG and U251MG) and total protein, cytoplasmic protein, and nuclear protein were extracted to measure YBX1 expression. After silencing KPNB1, the total YBX1 protein did not change, but the expression of YBX1 in the cytoplasmic fraction increased, and the expression of nuclear YBX1 protein decreased (Fig. 5E). In contrast, the nuclear expression of YBX1 increased after overexpression of KPNB1 (Fig. 5F). Immunofluorescence staining confirmed that the cytoplasmic localization of YBX1 increased, and the nuclear expression decreased after KPNB1 knockdown (Fig. 5G). Moreover, we performed a proximity ligation assay (PLA) to confirm the interaction between KPNB1 and YBX1 in U251MG cells (Fig. 5H). These results suggested that KPNB1 interacted with YBX1 in GBM and regulated the nuclear translocation of YBX1 to promote tumor growth.

### Nuclear localization of YBX1 enhanced NLGN3 expression in GBM

**Fig. 6:**
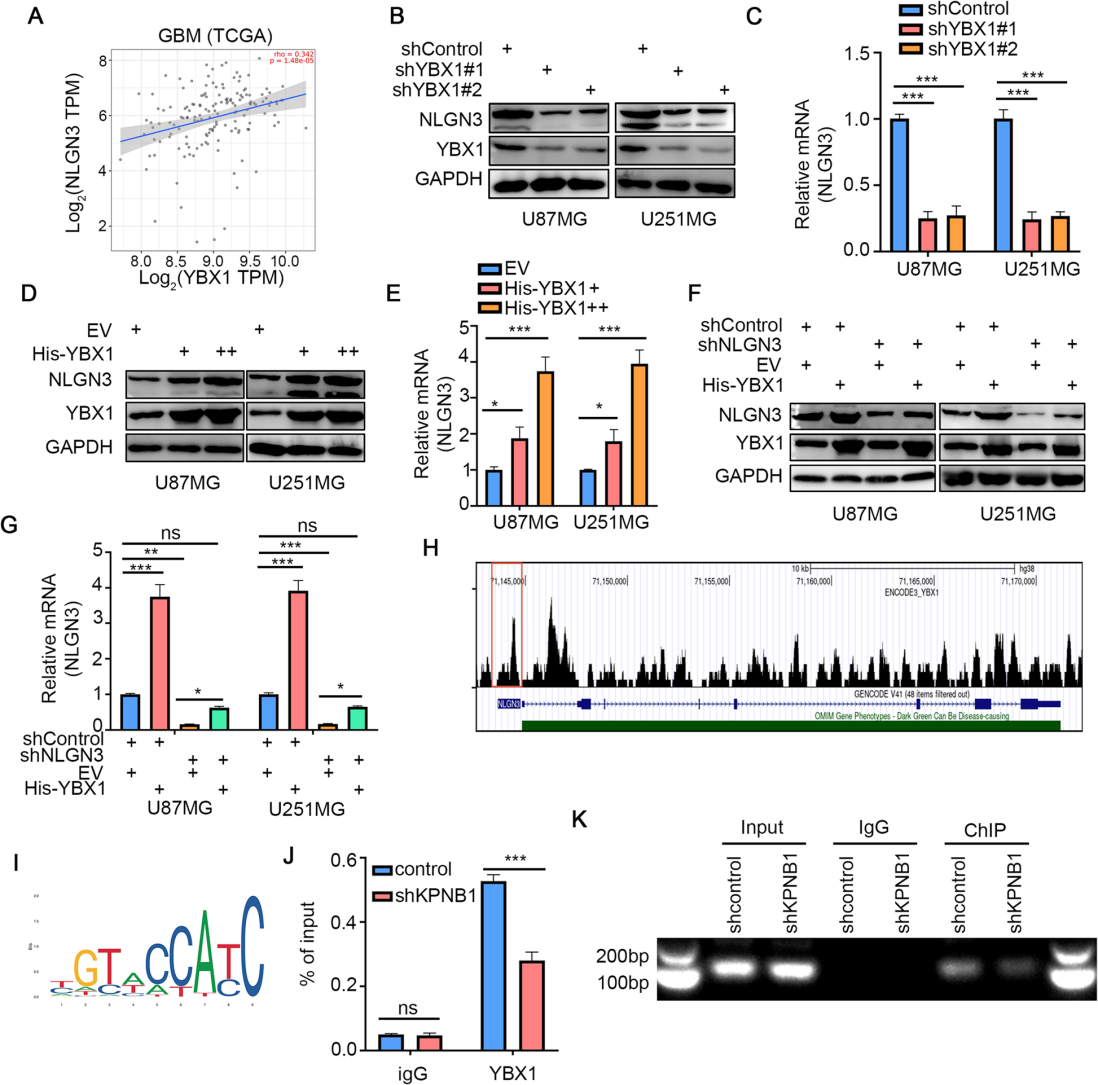
Nuclear localization of YBX1 enhanced NLGN3 expression in GBM. A. TCGA database showed that the expression of YBX1 was positively correlated with that of NLGN3 at the mRNA level. B and C. U87MG and U251MG cells were infected with shYBX1#1 and shYBX1#2. Cells were collected for Western blot analysis (B) and RT-qPCR (C). Data presented as the mean ± SD of three independent experiments, ***P < 0.001, one-way ANOVA. D and E. U87MG and U251MG cells were infected with YBX1 plasmids. Cells were collected for Western blot analysis (D) and RT-qPCR (E). Data presented as the mean ± SD of three independent experiments, ***P < 0.001, one-way ANOVA. F and G. U87MG and U251MG cells were infected with shControl or shNLGN3 for 48 h. Then, cells were transfected with pcDNA3.1 or His-YBX1 as indicated. After 24 h, cells were harvested for Western blotting analysis (F) and RT-qPCR analysis (G). Data presented as the mean ± SD of three independent experiments, ***P < 0.001, one-way ANOVA. H. ChIP-Seq peaks were mapped on the UCSC genome browser. Data presented as the mean ± SD of three independent experiments, ***P < 0.001, ns not significant, one-way ANOVA. I. The DNA motif of HSF4 was prepared using JASPAR online tools. J. Relative quantification of ChIP-qPCR. All data are shown as the mean ± SD from three replicates. ***P < 0.001, unpaired Student’s *t*-test. K. DNA electrophoresis of the products from the ChIP assay

Through TCGA database analysis, we found that the expression of YBX1 was positively correlated with NLGN3 (Fig. 6A). Next, to verify the regulatory effects of YBX1 on NLGN3, Western blot and RT-qPCR were performed, which showed that NLGN3 expression was downregulated after YBX1 silencing (Fig. 6B and C). Moreover, NLGN3 expression was also upregulated after YBX1 overexpression (Fig. 6D and E). In addition, the downregulation of NLGN3 following NGLN3 knockdown alone was rescued by overexpressing YBX1 (Fig. 6F and G). Both the colony formation assay and CCK8 assay demonstrated that overexpression of YBX1 could promote the proliferation of GBM cells and knockdown of NLGN3 could inhibit GBM cell proliferation; moreover, this inhibitory effect could be rescued by overexpression of YBX1 (Supplementary Fig. S3F-H). Next, we predicted a YBX1 binding peak in the promoter of NLGN3 by using ChIP-Atlas (Fig. 6H). The DNA motif of YBX1 was presented using JASPAR online tools (Fig. 6I). We then tested this interaction by ChIP qPCR and gel electrophoresis assay. YBX1 bonded to the promoter region of NLGN3, and this binding was significantly reduced after KPNB1 knockdown (Fig. 6J and K). These data suggested that YBX1 promoted NLGN3 transcription after nuclear translocation via KPNB1, which contributed to GBM progression.

### USP7 could bind to KPNB1 and stabilize KPNB1 expression by deubiquitination

**Fig. 7:**
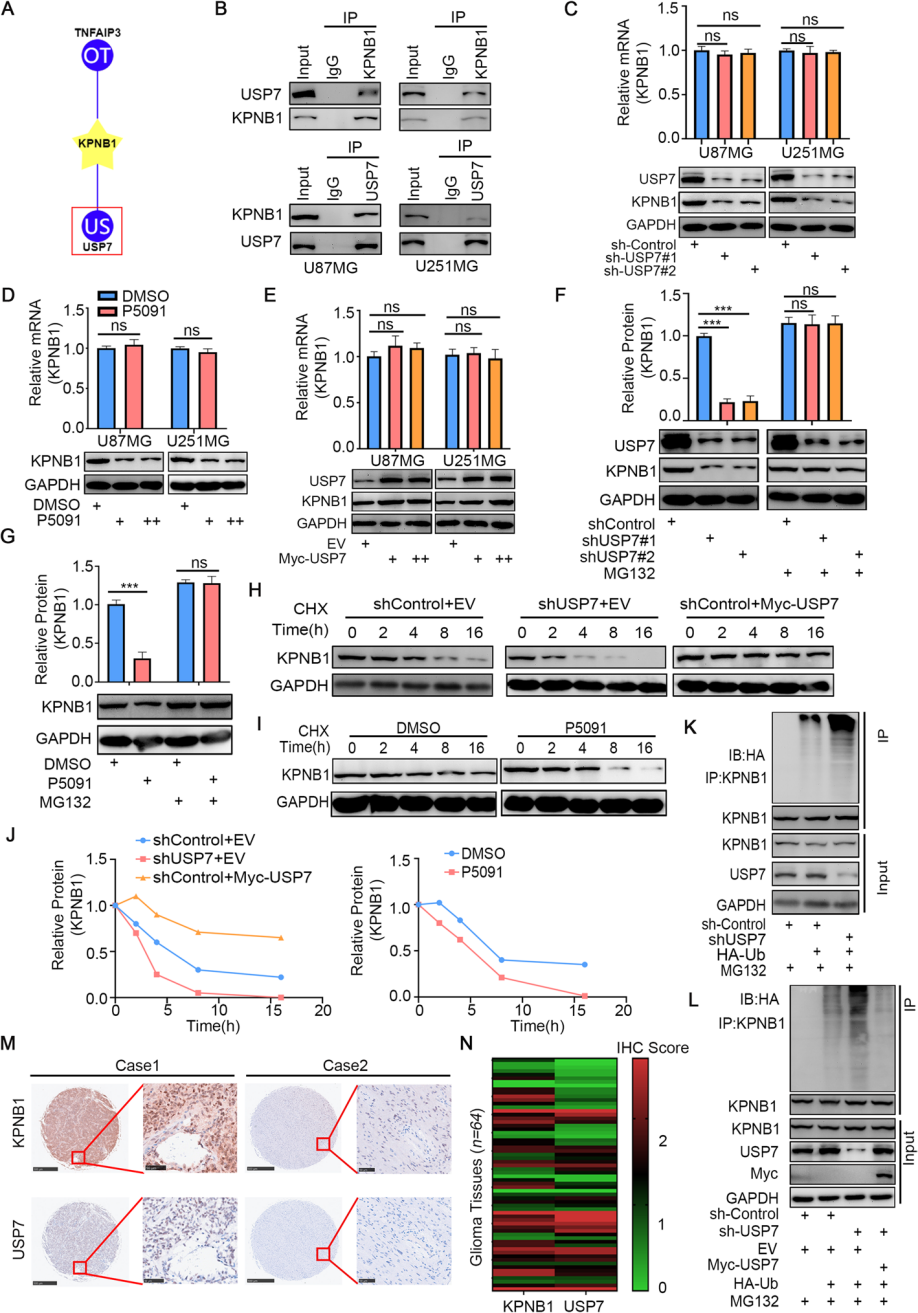
USP7 bound to KPNB1 and stabilized KPNB1 expression by deubiquitination. A. UbiBrowser identified deubiquitinases (DUBs) that might interact with KPNB1. B. Western blot analysis showing the reciprocal co-IP in U87MG and U251MG cells demonstrating the binding of endogenously expressed KPNB1 to USP7. C. U87MG and U251MG cells were infected with shUSP7#1 and shUSP7#2. Cells were collected for Western blot analysis and RT-qPCR. Data presented as the mean ± SD of three independent experiments, ns: not significant, one-way ANOVA. D. U87MG and U251MG cells were treated with or without USP7 inhibitor P5091 (10 μM) for 48 h. Then, cells were harvested for RT-qPCR and Western blotting analysis. Data presented as the mean ± SD of three independent experiments, ns: not significant, one-way ANOVA. E. U87MG and U251MG cells were infected with USP7 plasmids. Cells were collected for Western blot analysis and RT-qPCR. Data presented as the mean ± SD of three independent experiments, ns: not significant, one-way ANOVA. F. U251MG cells were infected with shUSP7#1 and shUSP7#2. After 48 h or 72 h, the corresponding groups were treated with MG132 for another 12 h. All cells were harvested for Western blotting analysis. G. U251MG cells were treated with or without USP7 inhibitor P5091 (10 μM) for 48 h then treated with MG132 for another 12 h. All cells were harvested for Western blotting analysis. H-J. U251MG cells were infected with shUSP7 or Myc USP7 (H) or treated with P5091 (I). Cells were treated with cycloheximide (CHX), and all cells were collected for Western blotting analysis at different time points. The half-life of KPNB1 protein is shown (J). K-L. U251MG cells were infected with shUSP7 (K) or Myc USP7 (L) and treated with MG132 for 12 h. Then, cells were collected for Western blot analysis. M-N. GBM tissue microarrays were stained with antibodies against USP7 and KPNB1. The correlation of these two proteins is shown in panel (N). Spearman correlation was used to determine statistical significance; *P* < 0.001

The regulation of protein expression is undoubtedly a complex process [60]. Ubiquitination, a critical post-translational modification, is currently the focus in studying the regulation of eukaryotic signaling in normal and disease states, as the ubiquitin-proteasome pathway is the most important known protein degradation pathway of high specificity in all eukaryotic organisms [61]. To determine the mechanism of KPNB1 overexpression in GBM, UbiBrowser (http://ubibrowser.bio-it.cn/) was used. The results showed that the DUBs TNFIAP3 and USP7 were closely associated with KPNB1 (Fig. 7A). Co-IP confirmed the interaction between endogenously expressed USP7 and KPNB1 in U87MG and U251MG cell lines (Fig. 7B). Next, the knockdown of USP7 in both cell lines showed that KPNB1 was downregulated at the protein level but not at the mRNA level (Fig. 7C). The results were consistent with the effects of P5091, an inhibitor of USP7 (Fig. 7D). Overexpression of USP7 also resulted in increased KPNB1 protein levels, but the mRNA levels were not perturbed (Fig. 7E). The above results suggested that USP7 regulation of KPNB1 might act through post-translational mechanisms and was unlikely to interfere with mRNA transcription. To further test this hypothesis, we added the proteasome inhibitor MG132 to both cell lines with USP7 knockdown and found that KPNB1 expression was no longer affected by USP7 knockdown (Fig. 7F). P5091 also produced consistent results (Fig. 7G). A subsequent protein half-life assay confirmed that KPNB1 protein degradation was accelerated after USP7 knockdown (Fig. 7H and J) and slowed after USP7 overexpression (Fig. 7H and J). Consistently, P5091 also accelerated the degradation of KPNB1 (Fig. 7I and J). Moreover, a protein polyubiquitination assay further confirmed that USP7 stabilized KPNB1 expression by deubiquitination (Fig. 7K and L). Additionally, we used a GBM tissue microarray to verify the positive correlation between USP7 and KPNB1 expression in gliomas (Fig. 7M and N). These results suggested that USP7 interacted with KPNB1, with a positive correlation between USP7 and KPNB1 expression. Importantly, our data implied that USP7 enhanced KPNB1 protein levels in GBM by deubiquitination.

### USP7/KPNB1/YBX1 axis regulated NLGN3 expression and promoted tumorigenesis in GBM

**Fig. 8:**
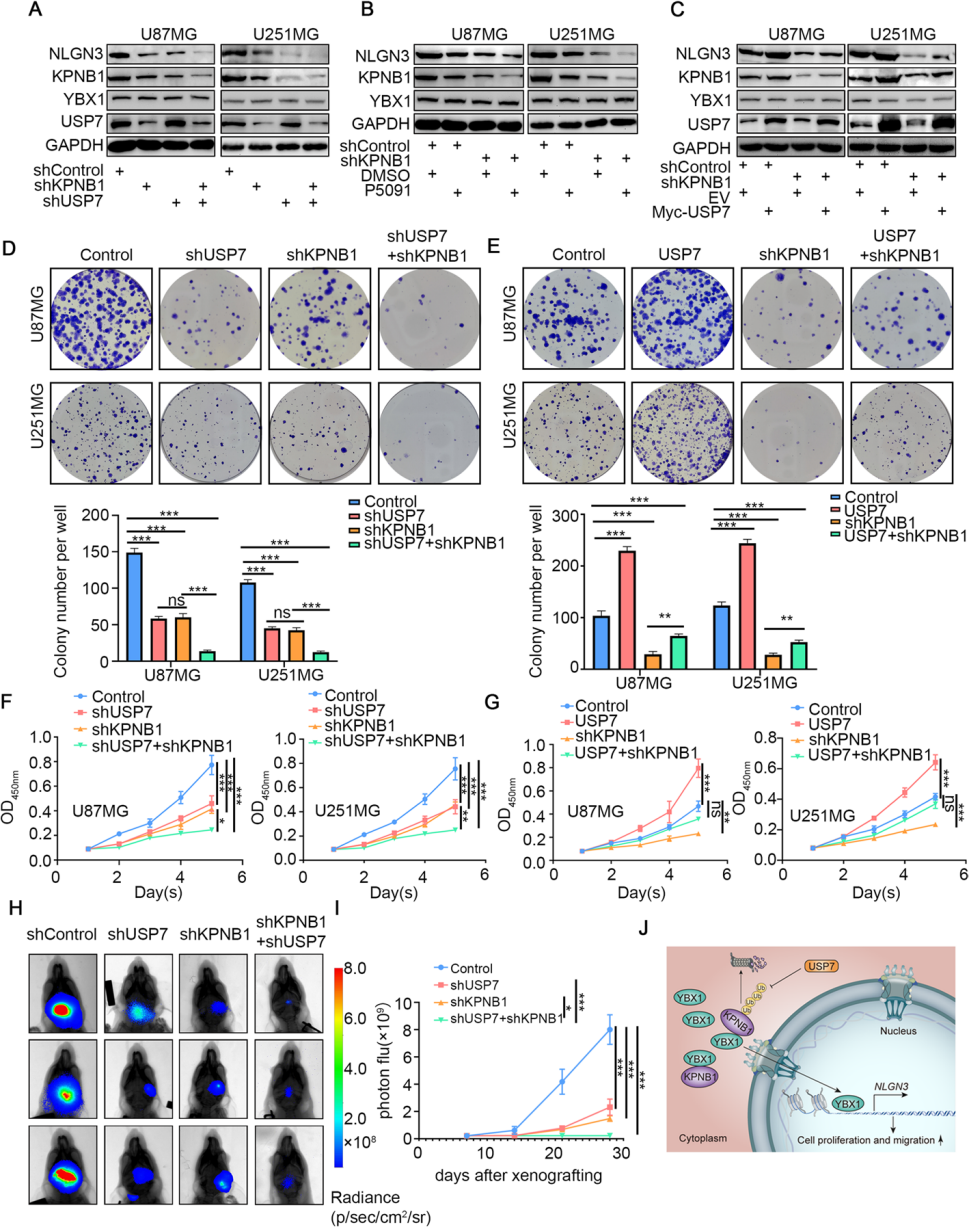
USP7/KPNB1/YBX1/NLGN3 axis promoted tumorigenesis in GBM. A, D, and F. U87MG and U251MG cells were infected with shUSP7, shKPNB1, or both. Cells were collected for Western blot (A), colony formation assay (D), and CCK8 assay (F). Data expressed as mean ± SD, *n* = 10 per group. *P < 0.05; **P < 0.01; ***P < 0.001, one-way ANOVA. B. U87MG and U251MG cells were treated with P5091 and then collected for Western blot. C, E, and G. U87MG and U251MG cells were infected with Control or shKPNB1 for 48 h. Then, cells were transfected with pcDNA3.1 or Myc USP7 as indicated. After 24 h, cells were harvested for Western blotting analysis (C), colony formation assay (E), and CCK8 assay (G). Data expressed as mean ± SD, *n* = 10 per group. *P < 0.05; **P < 0.01; ***P < 0.001, one-way ANOVA. H. U251MG cells were infected with shUSP7 and shKPNB1. After puromycin selection, cells were administered to nude mice via intracranial injection to establish a xenograft model. Representative bioluminescence imaging of a tumor at day 28 is shown. I. Quantitative assessments of tumor growth following implantation. Data expressed as mean ± SD, *n* = 10 per group. *P < 0.05; **P < 0.01; ***P < 0.001, one-way ANOVA. J. Schematic diagram summarizing the role of the USP7/KPNB1/YBX1/NLGN3 axis in promoting tumorigenesis in GBM

We next sought to further elucidate the role of USP7 in GBM progression and clarify whether this role was dependent on KPNB1. Based on the above experiments, we identified the molecular mechanism by which KPNB1 regulated GBM progression. Next, we knocked down KPNB1 while suppressing the expression of USP7 in U87MG and U251MG cell lines. The knockdown effect was verified by Western blotting (Fig. 8A), and we found that the KPNB1-USP7 combined knockdown achieved a greater effect to decrease NLGN3 protein expression compared with the single knockdown groups (Fig. 8A). However, the protein level of YBX1 was not changed (Fig. 8A). Moreover, both the colony formation assay and CCK8 assay demonstrated that the cell proliferation ability of the combined knockdown group was further inhibited compared with that of the single knockdown groups (Fig. 8D-F), and the tumor-suppressing effects of the combined knockdown were further verified in vivo (Fig. 8H-I). The use of P5091 achieved results consistent with that of USP7 knockdown (Fig. 8B). Conversely, USP7 overexpression rescued the downregulation of NLGN3 caused by KPNB1 knockdown (Fig. 8C). In addition, colony formation and CCK8 assays confirmed that overexpression of USP7 promoted GBM cell proliferation and was sufficient to rescue the decrease in GBM cell proliferation caused by KPNB1 knockdown (Fig. 8E-G). These results suggested that USP7 regulated NLGN3 expression by stabilizing KPNB1 expression, and this effect was indirectly regulated by the nuclear translocation of YBX1. Overall, these data indicated that the USP7/KPNB1/YBX1/NLGN3 axis actively regulated tumor progression in GBM (Fig. 8J).

## Discussion

As the most common primary intracranial malignant tumor, GBM is frequently associated with poor prognosis, with a median survival of less than 2 years [5]. Incidence of GBM demonstrates a sexual dimorphism, with GBM more commonly occurring in males; incidence also increases with age [62, 63]. Currently, standard therapies for newly diagnosed GBM include surgical treatment and postoperative temozolomide combined with radiotherapy, but the overall prognosis remains poor, and long-term survival is rare [64]. The advent of immunotherapy has transformed the treatment outcomes of many cancers, but its use for CNS tumors has been limited by their unique immune microenvironment [65]. Therefore, immunotherapy has not achieved good efficacy in glioma patients. Hence, despite substantial efforts to explore immunotherapy and precision oncology approaches, there is clearly a need for better treatment options. Significant challenges to the development of novel therapeutic modalities include not only the unique immune microenvironment but also the unique anatomical structures associated with GBM (e.g., blood-brain barrier). Therefore, there is an urgent need to identify novel and highly effective targets to ultimately improve the outcomes of patients with GBM.

KPNB1 is a nuclear transport receptor that can interact with KPNA to transport molecules with classical nuclear localization signals (NLS) into the nucleus, as well as molecules with non-classical NLS in a KPNA-independent manner [66]. KPNB1 plays an important role in a variety of cancers, and in this study, we revealed a novel regulatory mechanism of KPNB1 in GBM cells. We elucidate for the first time the specific mechanisms downstream of KPNB1 as a key molecular target for predicting GBM prognosis. The high expression of KPNB1 in GBM could promote the growth, proliferation, and migration of glioma cells and enhance tumor growth in vivo. It was also found that the knockdown of KPNB1 promoted TMZ sensitivity. KPNB1 could promote the function of YBX1 as a transcriptional activator by facilitating its nuclear import. However, the mechanism of the interaction between KPNB1 and YBX1 remains uncertain. Notably, YBX1 is highly enriched in the transcriptional start site of its downstream NLGN3. Therefore, we suggested that the increased nuclear import of YBX1 might promote the expression of NLGN3. As a postsynaptic adhesion molecule, NLGN3 itself is also highly expressed in glioma cells, and previous studies have reported that NLGN3 can promote glioma progression.

In most eukaryotic cells, ubiquitination is a reversible process driven by a series of enzymes. Protein deubiquitination is retained by deubiquitinating enzymes [67]. The regulatory mechanism of KPNB1 ubiquitination and deubiquitination in tumor cells remains unclear. The current study identified USP7 as a member of the ubiquitin-specific processing protease family that could stabilize KPNB1 expression by deubiquitination. USP7 is a member of the ubiquitin-specific protease family and can scavenge the ubiquitin modification of the substrate, thereby protecting the substrate from proteolysis [42, 68]. Previous studies have shown that USP7 plays different roles in the development of cancer [69]. A recent study showed that high USP7 expression was associated with poor survival and later tumor stage in GBM [70]. However, the role of USP7 in GBM progression requires further investigation. The current study initially found that USP7 expression was upregulated in GBM tissues. Subsequently, USP7 promoted GBM proliferation and invasion by stabilizing the KPNB1-YBX1-NLGN3 signaling axis in vitro and in vivo. P5091, as a novel specific inhibitor of USP7, might have clinical significance by providing an effective way to treat GBM.

It is well known that GBM is an incurable malignant brain tumor due to its high cellular heterogeneity, caused by the complex brain genetic heterogeneity of patients [71]. A subpopulation of cells in gliomas, known as brain tumor stem cells, is an aggressive, stem-like cell population that is resistant to chemotherapy and radiation therapy and may be responsible for tumor recurrence after the application of standard therapies [72]. The glioblastoma stem cells (GSCs) are distinguished by their unlimited self-renewal capability and differentiation potential, which contribute to the tumorigenesis, progression, and recurrence of GBM [73, 74]. The current study only explored the role of the USP7-KPNB1-YBX1-NLGN3 axis in GBM cell lines. However, the effects of this signal axis on the activity of GSC will be investigated in the future. Therefore, our future study will focus on further elucidation of whether KPNB1 is the molecular mechanism by which GSCs maintain stemness. The Cancer Genome Atlas Network describes a robust gene expression-based molecular classification of GBM into mesenchymal, classical, and proneural subtypes, providing a potential approach to improve the treatment of glioblastoma [75]. Studies have found that patients with different subtypes have different prognosis, with proneuronal and classical GBM showing a better prognosis than mesenchymal tumors [76, 77]. Therefore, these subtype-related differences provide different therapeutic directions to guide the application of targeted therapeutic therapies in GBM. An assessment of the response of mesenchymal, classical, and preneuronal GBM subtypes to treatment with the USP7-KPNB1-YBX1-NLGN3 signaling axis is missing from this study. We next plan to further investigate the potential differences in the therapeutic response of different subtypes of GBM to this signaling axis, thereby providing new insights for clinical application. Our study has additional limitations, such as YBX1 may not be the only transcription factor, but based on our results, YBX1 is still the most likely transcription factor regulated by KPNB1. At the same time, we know that USP7 is a deubiquitinating enzyme, and its deubiquitinating activity is usually explored in research to maintain protein stability. However, it has been reported that USP7 can interact with PcG proteins to regulate molecular transcription [78]. Thus, the regulation of NLGN3 by USP7 may not only indirectly promote NLGN3 transcription by stabilizing KPNB1 expression by deubiquitination. USP7 may also directly regulate NLGN3 expression. These questions need to be further explored. To sum up, this study elucidated that KPNB1 promoted glioma progression through the YBX1-NLGN3 axis while USP7 stabilized its high expression, which may provide a new therapeutic target for GBM.

## Conclusions

Aberrant transport of proteins into the nucleoplasm disrupts cell homeostasis and promotes cancer progression. KPNB1 is a nuclear receptor that is highly expressed in a variety of cancers and promotes tumor progression. However, few studies have investigated its function in mediating the nuclear translocation of macromolecules in the context of cancer. Our study found that KPNB1 was highly expressed in GBM and associated with poor clinical prognosis. Moreover, KPNB1 could regulate the nuclear translocation of YBX1. Transcriptome sequencing demonstrated that KPNB1 could regulate the expression of NLGN3, which has been previously reported as a postsynaptic membrane protein that is highly expressed in the central nervous system and key to glioma progression. In our study, we found that YBX1 exhibited a binding peak with the promoter region of NLGN3, and this binding was regulated by KPNB1. In addition, we also showed that USP7 could stabilize KPNB1 expression by deubiquitination, which could be inhibited by the USP7 inhibitor P5091. Taken together, our results identified the USP7/KPNB1/YBX1/NLGN3 axis as a regulator of GBM progression and provided new insights into the clinical treatment of GBM.

### Supplementary Information

**Additional file 1: Supplementary Fig. S1.** KPNB1 regulated GBM progression in vitro. A-E. U87MG and U251MG cells were infected with lentivirus vectors expressing shKPNB1#1 and shKPNB1#2. Cells were collected for Western blot analysis (A), RT-qPCR (B), CCK8 assay (C), colony formation assay (D), and Transwell invasion assay (E). Data presented as the mean ± SD of three independent experiments, ***P < 0.001, one-way ANOVA. F-J. U87MG and U251MG cells were infected with lentivirus vectors expressing KPNB1 plasmids. Cells were collected for Western blot analysis (*F*), RT-qPCR (G), CCK8 assay (H), colony formation assay (I), and Transwell invasion assay (J). K and L. U87MG and U251MG cells were infected with lentivirus vectors expressing shKPNB1. The control and knockdown KPNB1 groups were treated with TMZ(10 μM), respectively. CCK8 was used to detect cell proliferation (K), and flow cytometry was used to detect cell apoptosis (L). Data presented as the mean ± SD of three independent experiments; ***P < 0.001, one-way ANOVA.

**Additional file 2: Supplementary Fig. S2.** Overexpression of KPNB1 rescued the inhibition of GBM cell proliferation and invasion caused by NLGN3 knockdown. U87MG and U251MG cells were infected with Control or shNLGN3 for 48 h. Then, cells were transfected with pcDNA3.1 or Flag KPNB1 as indicated. After 24 h, cells were harvested for Western blotting analysis (A), RT-qPCR (B), CCK8 assay (C), colony formation assay (D), and Transwell invasion assay (E). Data presented as the mean ± SD of three independent experiments; ***P < 0.001, one-way ANOVA.

**Additional file 3: Supplementary Fig. S3.** KPNB1/YBX1/NLGN3 axis regulated GBM cell proliferation in vitro. A-C. U87MG and U251MG cells were infected with shKPNB1, shYBX1, or both. Cells were collected for Western blot (A). The relative protein expressions of YBX1 and NLGN3 are shown in B and C, respectively. D. Cells were also collected for colony formation assay, and CCK8 assay (E). F-H. U87MG and U251MG cells were infected with Control or shNLGN3 for 48 h. Then, cells were transfected with EV or His YBX1 as indicated. After 24 h, cells were harvested for Western blotting analysis. Quantification of protein expression is presented in panel F. Cells were also collected for colony formation assay (G) and CCK8 assay (H). Data presented as mean ± SD of three independent experiments; ***P < 0.001, one-way ANOVA.

**Additional file 4: Supplementary Table S1.** siRNA sequences.

**Additional file 5: Supplementary Table S2.** Primer sequences for lentivirus shRNA and amplification genes.

**Additional file 6: Supplementary Table S3.** Primer sequences for RT-qPCR.

**Additional file 7: Supplementary Table S4.** Primer sequences for ChIP.

**Additional file 8: Table S5.** RNA-seq of siKPNB1 vs. siControl.

**Additional file 9.**

## Data Availability

The data supporting the findings of this article is included within this article and its additional files. The RNA-seq data is provided in Supplementary Table S5. The datasets used and/or analyzed during the current study are available from the corresponding authors (yangkunyu@hust.edu.cn) upon reasonable request.

## Abbreviations

GBM: Glioblastoma
NLGN3: Neuroligin-3
KPNB1: Karyopherin β1
PD-L1: Programmed cell death 1 ligand 1
Her2: Human epidermal growthfactor receptor 2
Wnt/β-catenin pathway: Canonical Wnt/β-catenin pathway
Bcl-2 family: B cell lymphoma gene-2
DR5: Death receptor 5
Mcl-1: Myeloid cell leukemia—1
RTKs: Receptor tyrosine kinase
PI3K–mTOR: Phosphoinosmde-3-kinase/the mammalian target of rapamycin
CSC: Cancer stem cell
USP7: Ubiquitin specific peptidase 7
Ub: Ubiquitin
siRNA: small interfering RNA
IP: Immunoprecipitation
Co-IP: Coimmunoprecipitation
SDS-PAGE: Sodium dodecyl-sulfate polyacrylamide gel electrophoresis
RNA-seq: RNA sequencing
TCGA: The cancer genome atlas
PR: Partial response
CR: Completely mitigated
PD: Progressive disease
SD: Stable disease
WHO: World health organization
DEGs: Differential gene expression
YBX1: Y-box binding protein 1
ChIP: Chromatin immunoprecipitation
DUBs: Deubiquitinases
NLS: Nuclear localization signals
TMZ: Temozolomide
PLA: Proximity ligation assay
