# Supplementary figures and images for: Stabilization of KPNB1 by deubiquitinase USP7 promotes glioblastoma progression through the YBX1-NLGN3 axis
Source: J Exp Clin Cancer Res. 2024 Jan 23;43:28. doi: 10.1186/s13046-024-02954-8 (PMC11040697; doi:10.1186/s13046-024-02954-8)

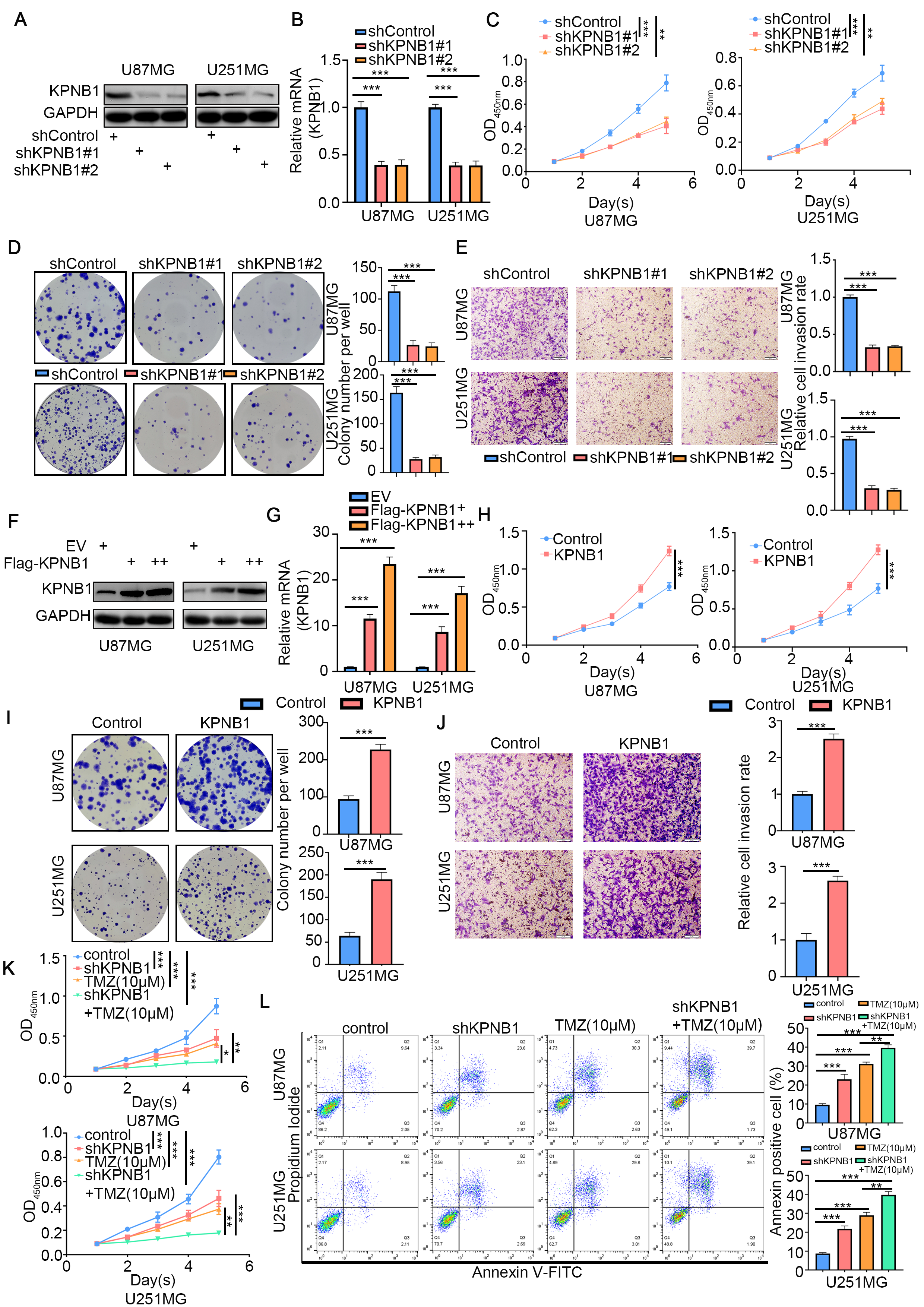

Supplement: Supplementary file 1 — Additional file 1: Supplementary Fig. S1. KPNB1 regulated GBM progression in vitro. A-E. U87MG and U251MG cells were infected with lentivirus vectors expressing shKPNB1#1 and shKPNB1#2. Cells were collected for Western blot analysis (A), RT-qPCR (B), CCK8 assay (C), colony formation assay (D), and Transwell invasion assay (E). Data presented as the mean ± SD of three independent experiments, ***P < 0.001, one-way ANOVA. F-J. U87MG and U251MG cells were infected with lentivirus vectors expressing KPNB1 plasmids. Cells were collected for Western blot analysis (F), RT-qPCR (G), CCK8 assay (H), colony formation assay (I), and Transwell invasion assay (J). K and L. U87MG and U251MG cells were infected with lentivirus vectors expressing shKPNB1. The control and knockdown KPNB1 groups were treated with TMZ(10 μM), respectively. CCK8 was used to detect cell proliferation (K), and flow cytometry was used to detect cell apoptosis (L). Data presented as the mean ± SD of three independent experiments; ***P < 0.001, one-way ANOVA. [file 13046_2024_2954_MOESM1_ESM.tif]

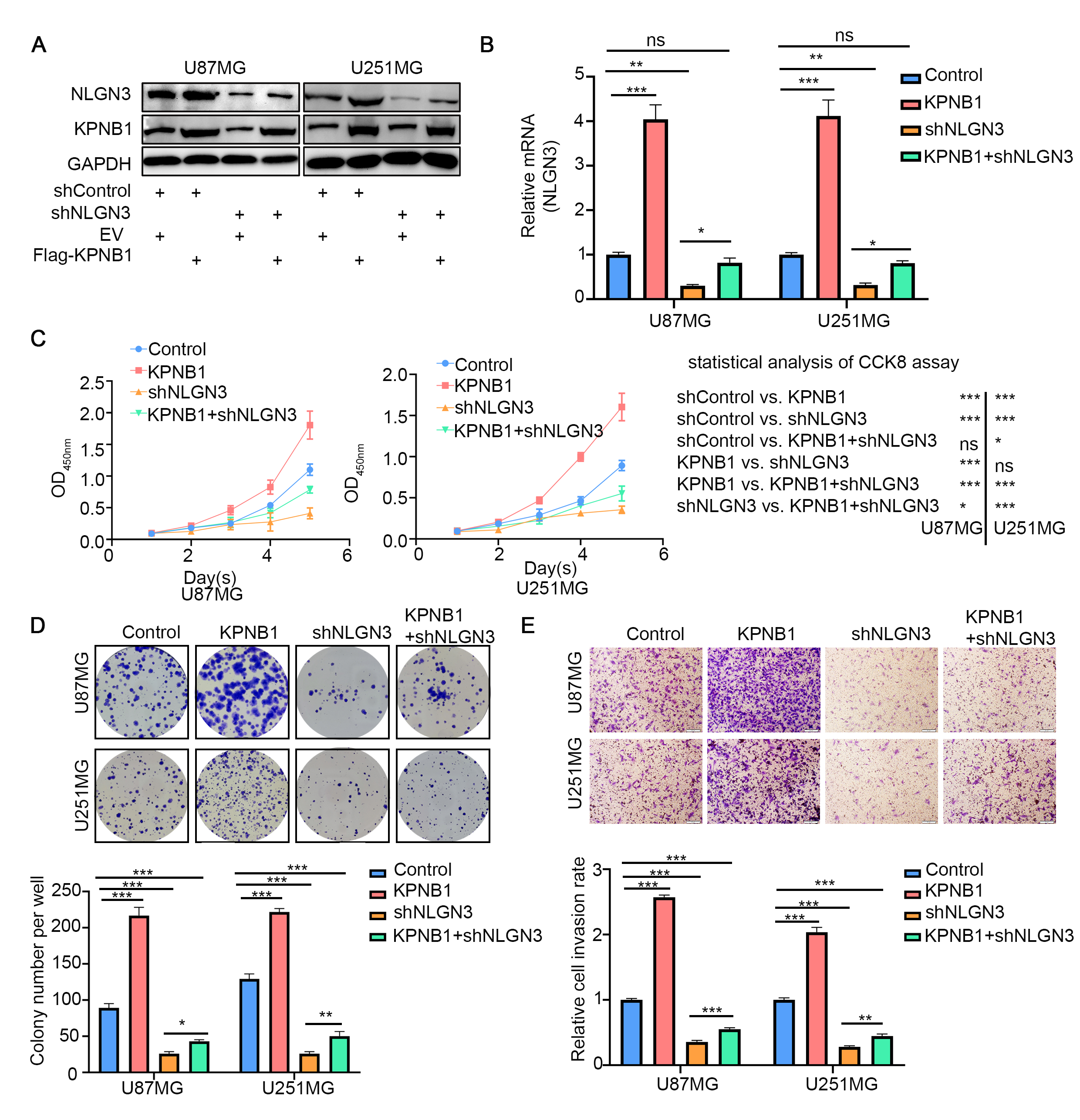

Supplement: Supplementary file 2 — Additional file 2: Supplementary Fig. S2. Overexpression of KPNB1 rescued the inhibition of GBM cell proliferation and invasion caused by NLGN3 knockdown. U87MG and U251MG cells were infected with Control or shNLGN3 for 48 h. Then, cells were transfected with pcDNA3.1 or Flag KPNB1 as indicated. After 24 h, cells were harvested for Western blotting analysis (A), RT-qPCR (B), CCK8 assay (C), colony formation assay (D), and Transwell invasion assay (E). Data presented as the mean ± SD of three independent experiments; ***P < 0.001, one-way ANOVA. [file 13046_2024_2954_MOESM2_ESM.tif]

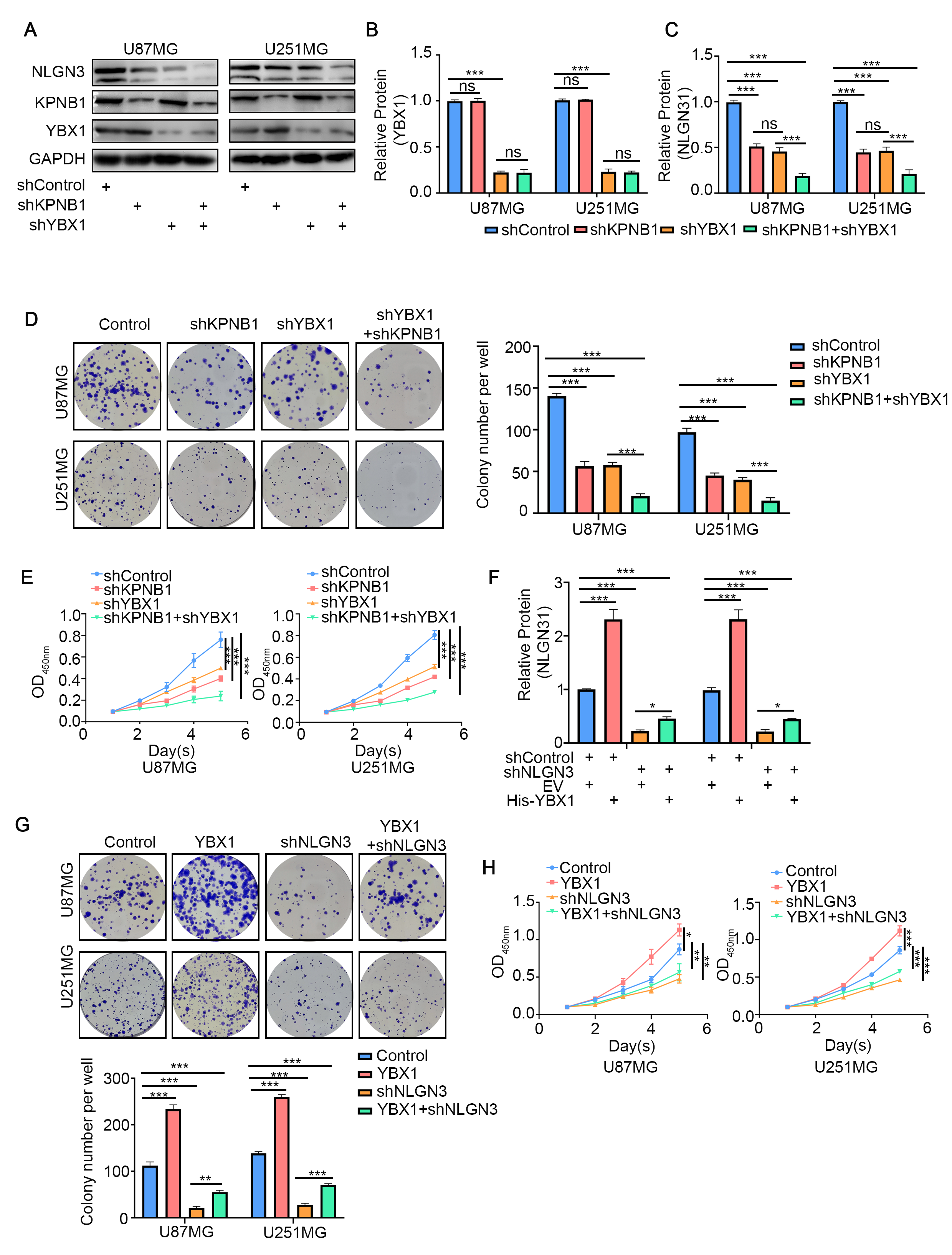

Supplement: Supplementary file 3 — Additional file 3: Supplementary Fig. S3. KPNB1/YBX1/NLGN3 axis regulated GBM cell proliferation in vitro. A-C. U87MG and U251MG cells were infected with shKPNB1, shYBX1, or both. Cells were collected for Western blot (A). The relative protein expressions of YBX1 and NLGN3 are shown in B and C, respectively. D. Cells were also collected for colony formation assay, and CCK8 assay (E). F-H. U87MG and U251MG cells were infected with Control or shNLGN3 for 48 h. Then, cells were transfected with EV or His YBX1 as indicated. After 24 h, cells were harvested for Western blotting analysis. Quantification of protein expression is presented in panel F. Cells were also collected for colony formation assay (G) and CCK8 assay (H). Data presented as mean ± SD of three independent experiments; ***P < 0.001, one-way ANOVA. [file 13046_2024_2954_MOESM3_ESM.tif]

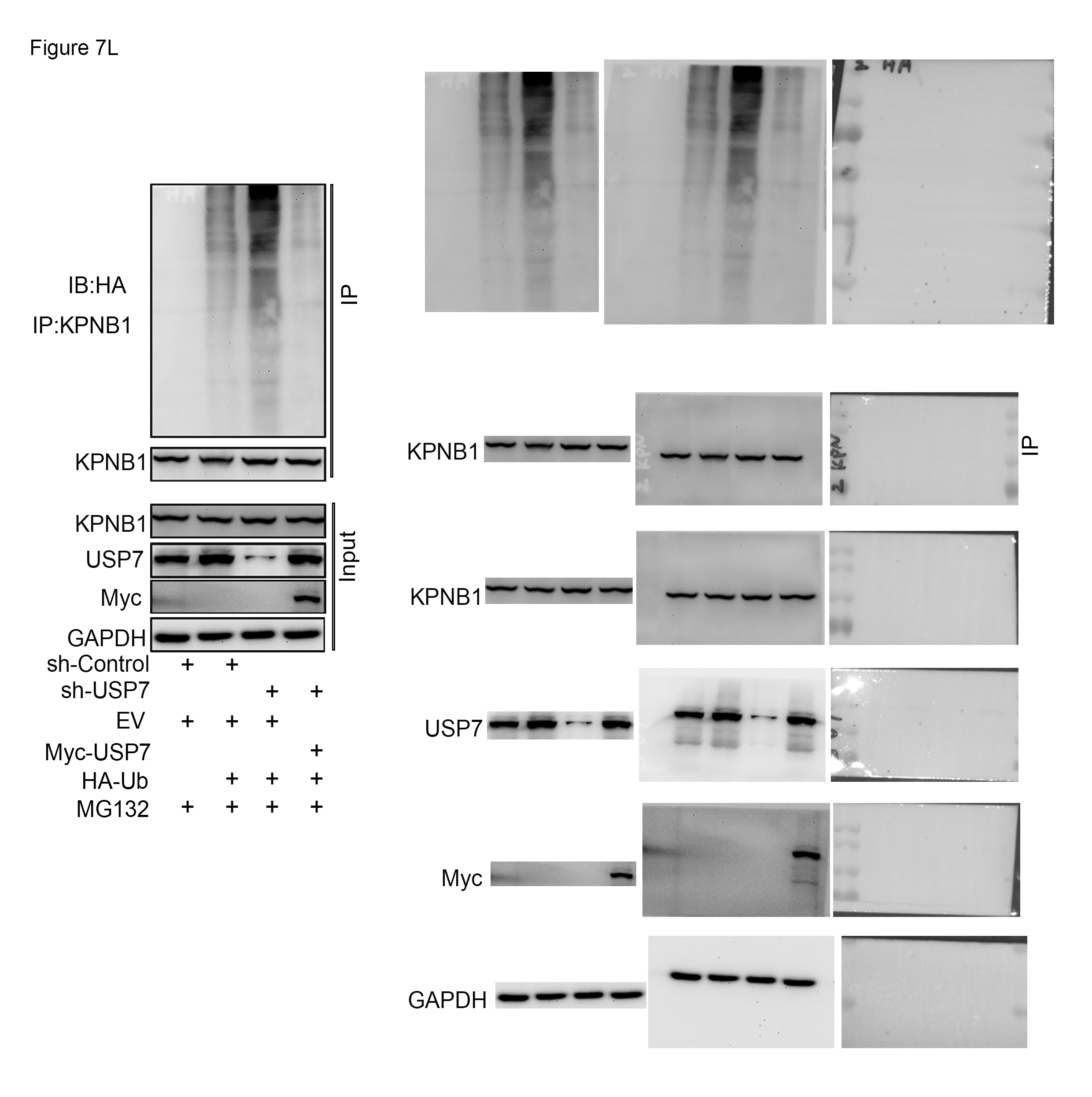

Supplement: Supplementary file 9 — Additional file 9. [file 13046_2024_2954_MOESM9_ESM.tif]
