# Supplementary material for: Stabilization of KPNB1 by deubiquitinase USP7 promotes glioblastoma progression through the YBX1-NLGN3 axis
Source: J Exp Clin Cancer Res. 2024 Jan 23;43:28. doi: 10.1186/s13046-024-02954-8 (PMC11040697; doi:10.1186/s13046-024-02954-8)
Supplement: Supplementary file 4 — Additional file 4: Supplementary Table S1. siRNA sequences. [file 13046_2024_2954_MOESM4_ESM.docx]

**Table**

**Supplementary Table S1, siRNA sequences**

| Gene | (5′-3′) |
| --- | --- |
| siKPNB1#1 | AAACGACTTTGGTCATCAT |
| siKPNB1#2 | CCAGAGCACATCCGATAGA |
| siKPNB1#3 | CGGAGATCGAAGACTAACA |
