# Supplementary material for: Stabilization of KPNB1 by deubiquitinase USP7 promotes glioblastoma progression through the YBX1-NLGN3 axis
Source: J Exp Clin Cancer Res. 2024 Jan 23;43:28. doi: 10.1186/s13046-024-02954-8 (PMC11040697; doi:10.1186/s13046-024-02954-8)
Supplement: Supplementary file 5 — Additional file 5: Supplementary Table S2. Primer sequences for lentivirus shRNA and amplification genes. [file 13046_2024_2954_MOESM5_ESM.docx]

**Table**

**Supplementary Table S2. Primer sequences for lentivirus shRNA and amplification genes**

| Gene | (5′-3′) |
| --- | --- |
| shControl | TTCTCCGAACGTGTCACGTTCAAGAGAACGTGACACGTTCGGAGAATTTT |
| shKPNB1#1 | CCGGGAAAGAAGAGCCTAGTAATAACTCGAGTTATTACTAGGCTCTTCTTC  TTTTTG |
| shKPNB1#2 | CCGGAGGCTATGCCCACCCTAATAGCTCGAGCTATTAGGGTGGGCATAGCC  TTTTTTG |

| shNLGN3#1 | CCGGGGCGAGGACTTAGCGGATAATCTCGAGATTATCCGCTAAGTCCTCGC  CTTTTTG |
| --- | --- |
| shNLGN3#2 | CCGGACCAAGGGTCCGAGATCATTACTCGAGTAATGATCTCGGACCCTTG  GTTTTTTG |
| shUSP7#1 | CCGGCCTGGATTTGTGGTTACGTTACTCGAGTAACGTAACCACAAATCCAGGTTTTTG |
| shUSP7#2 | CCGGCCAGCTAAGTATCAAAGGAAACTCGAGTTTCCTTTGATACTTAGCTGGTTTTTG |
| shYBX1#1 | CCGGAGCAGACCGTAACCATTATAGCTCGAGCTATAATGGTTACGGTCTGC  TTTTTTG |
| shYBX1#2 | CCGGCCAGTTCAAGGCAGTAAATATCTCGAGATATTTACTGCCTTGAACTG  GTTTTTG |
| KPNB1-F | GACTTCTAGAGCCACCCTAGAGAACCCACTGCTTAC |
| KPNB1-R | CAGTGGATCCGGAGCTGACACGGAAGAT |
| NLGN3-F | GACTTCTAGAGCCACCTTCCCCTGCAACTTCTCCAA |
| NLGN3-R | CAGTGGATCCCCTTAGTGGCCCGGTAATGA |
| USP7-F | CGCAAATGGGCGGTAGGCGTG |
| USP7-R | CGTTATCGTAGTGTTTAAAG |
| YBX1-F | GACTTCTAGAGCCACCGGTGTTCCAGTTCAAGGCAG |
| YBX1-R | CAGTGGATCCCCGCATGTAGTAAGGTGGGA |
