# Supplementary material for: Stabilization of KPNB1 by deubiquitinase USP7 promotes glioblastoma progression through the YBX1-NLGN3 axis
Source: J Exp Clin Cancer Res. 2024 Jan 23;43:28. doi: 10.1186/s13046-024-02954-8 (PMC11040697; doi:10.1186/s13046-024-02954-8)
Supplement: Supplementary file 6 — Additional file 6: Supplementary Table S3. Primer sequences for RT-qPCR. [file 13046_2024_2954_MOESM6_ESM.docx]

**Table**

**Supplementary Table S3. P r i m e r s e q u e n c e s f o r R T - q P C R.**

| Gene Name | Forward (5′-3′) | Reverse (5′-3′) |
| --- | --- | --- |
| GAPDH | AGAAGGCTGGGGCTCATTTG | AGGGGCCATCCACAGTCTTC |
| KPNB1 | CTTCAAATGTGTGCTGGGCT | TGCAGAACTCCTCAGGTTGT |
| YBX1 | GGTGTTCCAGTTCAAGGCAG | CCGCATGTAGTAAGGTGGGA |
| NLGN3 | TTCCCCTGCAACTTCTCCAA | CCTTAGTGGCCCGGTAATGA |
| USP7 | TACGTGACTTGCTCCCAGTT | ATCAACGCGGTGGTAGAGAT |
