# Supplementary material for: Stabilization of KPNB1 by deubiquitinase USP7 promotes glioblastoma progression through the YBX1-NLGN3 axis
Source: J Exp Clin Cancer Res. 2024 Jan 23;43:28. doi: 10.1186/s13046-024-02954-8 (PMC11040697; doi:10.1186/s13046-024-02954-8)
Supplement: Supplementary file 7 — Additional file 7: Supplementary Table S4. Primer sequences for ChIP. [file 13046_2024_2954_MOESM7_ESM.docx]

**Table**

**Supplementary Table S4. Primer sequences for ChIP**

| Gene | Forward | Reverse |
| --- | --- | --- |
| NLGN3 | GAATAGGTCGTGGAAGGA | CTGGGTAACTGCCAAGGT |
