# Supplementary material for: Stabilization of KPNB1 by deubiquitinase USP7 promotes glioblastoma progression through the YBX1-NLGN3 axis
Source: J Exp Clin Cancer Res. 2024 Jan 23;43:28. doi: 10.1186/s13046-024-02954-8 (PMC11040697; doi:10.1186/s13046-024-02954-8)
Supplement: Supplementary file 8 — Additional file 8: Table S5. RNA-seq of siKPNB1 vs. siControl. [file 13046_2024_2954_MOESM8_ESM.docx]

**Table S5 RNA-seq of siKPNB1 vs. siControl**

| gene_id | si_KPNB1 | si_Control | log2FoldChange | pvalue | padj | gene_name | gene_biotype |
| --- | --- | --- | --- | --- | --- | --- | --- |
| ENSG00000105825 | 16696.16066 | 6915.89113 | 1.271500887 | 0 | 0 | TFPI2 | protein_coding |
| ENSG00000108424 | 1966.926857 | 5809.766809 | -1.562948406 | 4.18E-277 | 3.54E-273 | KPNB1 | protein_coding |
| ENSG00000152952 | 6555.661929 | 13600.20806 | -1.052701559 | 1.30E-268 | 5.49E-265 | PLOD2 | protein_coding |
| ENSG00000099194 | 10871.43211 | 22080.18745 | -1.022182468 | 1.93E-259 | 5.45E-256 | SCD | protein_coding |
| ENSG00000139645 | 12945.13594 | 6412.861019 | 1.013180296 | 8.98E-222 | 1.69E-218 | ANKRD52 | protein_coding |
| ENSG00000068028 | 2627.995365 | 662.8239671 | 1.986775383 | 1.18E-206 | 1.99E-203 | RASSF1 | protein_coding |
| ENSG00000176171 | 1373.499392 | 3755.771932 | -1.450404626 | 5.81E-188 | 8.94E-185 | BNIP3 | protein_coding |
| ENSG00000103257 | 3033.14431 | 1084.582413 | 1.483567322 | 8.10E-183 | 1.14E-179 | SLC7A5 | protein_coding |
| ENSG00000166833 | 3723.00525 | 1425.782851 | 1.384644213 | 2.04E-180 | 2.65E-177 | NAV2 | protein_coding |
| ENSG00000117394 | 847.8594437 | 2591.093415 | -1.611864633 | 1.16E-173 | 1.40E-170 | SLC2A1 | protein_coding |
| ENSG00000166197 | 4149.137024 | 1706.16633 | 1.281765635 | 3.55E-169 | 4.01E-166 | NOLC1 | protein_coding |
| ENSG00000088325 | 6368.183434 | 3108.721651 | 1.034666921 | 7.42E-167 | 7.85E-164 | TPX2 | protein_coding |
| ENSG00000128510 | 1692.550517 | 4004.209152 | -1.24214228 | 1.67E-161 | 1.57E-158 | CPA4 | protein_coding |
| ENSG00000183160 | 219.3048215 | 1316.019166 | -2.583216931 | 2.90E-161 | 2.58E-158 | TMEM119 | protein_coding |
| ENSG00000185963 | 2858.116264 | 1035.498287 | 1.464427803 | 1.20E-157 | 1.02E-154 | BICD2 | protein_coding |
| ENSG00000101057 | 2866.938873 | 1128.634964 | 1.344301617 | 6.89E-148 | 5.30E-145 | MYBL2 | protein_coding |
| ENSG00000104419 | 1440.961843 | 3491.692474 | -1.277222208 | 3.06E-146 | 2.26E-143 | NDRG1 | protein_coding |
| ENSG00000159399 | 1602.338185 | 4017.541989 | -1.325696081 | 1.22E-145 | 8.57E-143 | HK2 | protein_coding |
| ENSG00000168209 | 1542.367213 | 3514.519265 | -1.188430607 | 1.64E-139 | 1.07E-136 | DDIT4 | protein_coding |
| ENSG00000154734 | 2870.464661 | 1092.537884 | 1.393031431 | 1.20E-135 | 7.24E-133 | ADAMTS1 | protein_coding |
| ENSG00000103888 | 780.8194703 | 2264.456604 | -1.535214495 | 1.32E-135 | 7.69E-133 | CEMIP | protein_coding |
| ENSG00000164176 | 478.5323681 | 1722.706041 | -1.847340715 | 1.21E-131 | 6.83E-129 | EDIL3 | protein_coding |
| ENSG00000129038 | 2904.401054 | 6425.904068 | -1.145357084 | 5.86E-127 | 3.20E-124 | LOXL1 | protein_coding |
| ENSG00000067082 | 4483.287816 | 2067.589904 | 1.117181983 | 1.32E-123 | 6.60E-121 | KLF6 | protein_coding |
| ENSG00000101638 | 382.9861593 | 1444.205449 | -1.915799551 | 4.87E-122 | 2.36E-119 | ST8SIA5 | protein_coding |
| ENSG00000145386 | 2900.83186 | 1254.607163 | 1.209726312 | 5.41E-122 | 2.55E-119 | CCNA2 | protein_coding |
| ENSG00000136997 | 1816.004251 | 652.8328778 | 1.475672532 | 2.47E-119 | 1.10E-116 | MYC | protein_coding |
| ENSG00000122884 | 1343.660463 | 3080.93677 | -1.197985151 | 3.58E-119 | 1.55E-116 | P4HA1 | protein_coding |
| ENSG00000151790 | 846.6378845 | 2211.932454 | -1.38684429 | 8.04E-119 | 3.32E-116 | TDO2 | protein_coding |
| ENSG00000148773 | 12130.5428 | 5929.806547 | 1.032599967 | 2.36E-118 | 9.52E-116 | MKI67 | protein_coding |
| ENSG00000089685 | 3840.998667 | 1734.864562 | 1.146095396 | 4.62E-118 | 1.82E-115 | BIRC5 | protein_coding |
| ENSG00000173706 | 2524.558286 | 5573.048719 | -1.142640166 | 7.83E-117 | 2.95E-114 | HEG1 | protein_coding |
| ENSG00000265972 | 1493.65773 | 3303.906883 | -1.146113538 | 3.41E-113 | 1.23E-110 | TXNIP | protein_coding |
| ENSG00000221955 | 364.0478187 | 1309.763322 | -1.845499774 | 1.08E-112 | 3.81E-110 | SLC12A8 | protein_coding |
| ENSG00000114268 | 1401.983197 | 3178.807037 | -1.181670931 | 1.18E-108 | 4.01E-106 | PFKFB4 | protein_coding |
| ENSG00000268089 | 4040.856851 | 1882.556718 | 1.101862146 | 1.84E-103 | 5.89E-101 | GABRQ | protein_coding |
| ENSG00000148841 | 2901.14395 | 1373.257182 | 1.079510892 | 2.18E-99 | 6.71E-97 | ITPRIP | protein_coding |
| ENSG00000166922 | 1081.032298 | 2433.682853 | -1.170366528 | 1.20E-98 | 3.63E-96 | SCG5 | protein_coding |
| ENSG00000138180 | 3734.145392 | 1776.612107 | 1.072465547 | 1.37E-94 | 3.87E-92 | CEP55 | protein_coding |
| ENSG00000068489 | 2155.008205 | 945.147791 | 1.189981092 | 5.45E-92 | 1.49E-89 | PRR11 | protein_coding |
| ENSG00000204160 | 1287.721646 | 453.7029081 | 1.504965562 | 2.85E-90 | 7.67E-88 | ZDHHC18 | protein_coding |
| ENSG00000170525 | 1195.353964 | 2547.969047 | -1.091434287 | 3.23E-84 | 7.70E-82 | PFKFB3 | protein_coding |
| ENSG00000099875 | 2716.526872 | 1289.467303 | 1.074340542 | 1.39E-83 | 3.27E-81 | MKNK2 | protein_coding |
| ENSG00000171992 | 674.1335652 | 1639.172271 | -1.281183535 | 3.89E-81 | 8.56E-79 | SYNPO | protein_coding |
| ENSG00000107159 | 125.0870463 | 647.428316 | -2.368472624 | 2.08E-80 | 4.46E-78 | CA9 | protein_coding |
| ENSG00000152558 | 602.1920409 | 1469.516914 | -1.287163438 | 2.43E-76 | 4.73E-74 | TMEM123 | protein_coding |
| ENSG00000164211 | 766.1680651 | 1813.844749 | -1.242879886 | 1.03E-75 | 1.97E-73 | STARD4 | protein_coding |
| ENSG00000137801 | 370.5379825 | 1083.904367 | -1.5484386 | 1.68E-75 | 3.17E-73 | THBS1 | protein_coding |
| ENSG00000166592 | 1308.325709 | 3101.171445 | -1.244460329 | 4.08E-75 | 7.51E-73 | RRAD | protein_coding |
| ENSG00000196139 | 589.5307437 | 1466.284305 | -1.316287192 | 2.52E-74 | 4.45E-72 | AKR1C3 | protein_coding |
| ENSG00000104936 | 851.5538795 | 1856.104887 | -1.123664603 | 2.16E-72 | 3.65E-70 | DMPK | protein_coding |
| ENSG00000185215 | 736.8801811 | 1660.633674 | -1.172856565 | 7.63E-68 | 1.15E-65 | TNFAIP2 | protein_coding |
| ENSG00000165244 | 1329.322594 | 528.802406 | 1.329711206 | 1.30E-67 | 1.95E-65 | ZNF367 | protein_coding |
| ENSG00000146670 | 2015.297195 | 933.3781439 | 1.110104243 | 3.01E-65 | 4.36E-63 | CDCA5 | protein_coding |
| ENSG00000187678 | 1870.536729 | 923.8022131 | 1.017465943 | 1.75E-64 | 2.51E-62 | SPRY4 | protein_coding |
| ENSG00000244242 | 360.165191 | 1036.746676 | -1.524112584 | 4.71E-64 | 6.60E-62 | IFITM10 | protein_coding |
| ENSG00000197405 | 162.3003876 | 620.004101 | -1.935015454 | 1.00E-63 | 1.38E-61 | C5AR1 | protein_coding |
| ENSG00000135476 | 1801.344524 | 890.4918169 | 1.016338254 | 4.46E-61 | 5.77E-59 | ESPL1 | protein_coding |
| ENSG00000151233 | 1726.975158 | 853.7654602 | 1.015869137 | 3.84E-60 | 4.89E-58 | GXYLT1 | protein_coding |
| ENSG00000137809 | 538.4942079 | 1251.432477 | -1.216781146 | 5.72E-59 | 6.97E-57 | ITGA11 | protein_coding |
| ENSG00000152256 | 468.7748715 | 1139.738479 | -1.280431951 | 8.88E-59 | 1.06E-56 | PDK1 | protein_coding |
| ENSG00000104415 | 55.06103421 | 366.2533094 | -2.732275591 | 1.17E-57 | 1.36E-55 | WISP1 | protein_coding |
| ENSG00000109107 | 205.5746051 | 724.4843648 | -1.814253149 | 1.81E-57 | 2.09E-55 | ALDOC | protein_coding |
| ENSG00000134690 | 1593.7352 | 784.949109 | 1.021193921 | 2.44E-54 | 2.57E-52 | CDCA8 | protein_coding |
| ENSG00000143847 | 102.8379963 | 476.8384878 | -2.21283838 | 9.50E-54 | 9.75E-52 | PPFIA4 | protein_coding |
| ENSG00000049249 | 137.8826453 | 528.8533977 | -1.939597594 | 4.84E-52 | 4.85E-50 | TNFRSF9 | protein_coding |
| ENSG00000165507 | 122.7342683 | 482.1231063 | -1.973519915 | 8.92E-52 | 8.79E-50 | DEPP1 | protein_coding |
| ENSG00000113721 | 505.5592394 | 1140.086899 | -1.171778765 | 1.55E-50 | 1.49E-48 | PDGFRB | protein_coding |
| ENSG00000079215 | 396.946841 | 980.8183072 | -1.304840648 | 3.00E-50 | 2.80E-48 | SLC1A3 | protein_coding |
| ENSG00000149090 | 642.5472537 | 1363.512583 | -1.085031688 | 4.40E-50 | 4.05E-48 | PAMR1 | protein_coding |
| ENSG00000108691 | 31.77452526 | 275.7828209 | -3.116008249 | 1.10E-49 | 9.90E-48 | CCL2 | protein_coding |
| ENSG00000001084 | 574.0415786 | 1206.5944 | -1.07182591 | 5.66E-49 | 5.02E-47 | GCLC | protein_coding |
| ENSG00000171051 | 641.6387557 | 1382.753713 | -1.107501655 | 2.63E-48 | 2.25E-46 | FPR1 | protein_coding |
| ENSG00000110076 | 561.801123 | 1168.547603 | -1.056136774 | 1.70E-46 | 1.39E-44 | NRXN2 | protein_coding |
| ENSG00000167513 | 1373.193096 | 665.2151215 | 1.045580289 | 3.33E-46 | 2.69E-44 | CDT1 | protein_coding |
| ENSG00000184524 | 484.112149 | 1143.280151 | -1.238641161 | 4.62E-46 | 3.71E-44 | CEND1 | protein_coding |
| ENSG00000168140 | 331.8652522 | 799.040628 | -1.267021101 | 4.95E-43 | 3.58E-41 | VASN | protein_coding |
| ENSG00000204381 | 304.3799419 | 779.7896502 | -1.356779872 | 5.13E-43 | 3.70E-41 | LAYN | protein_coding |
| ENSG00000168874 | 310.5489127 | 764.4295125 | -1.299254666 | 2.82E-42 | 1.98E-40 | ATOH8 | protein_coding |
| ENSG00000230882 | 938.9916996 | 316.3976136 | 1.570101145 | 2.18E-41 | 1.48E-39 | AC005077.4 | processed_pseudogene |
| ENSG00000198576 | 875.2035773 | 376.6941976 | 1.214190696 | 5.99E-40 | 3.88E-38 | ARC | protein_coding |
| ENSG00000178573 | 245.2473722 | 725.5766415 | -1.565484066 | 7.05E-40 | 4.54E-38 | MAF | protein_coding |
| ENSG00000023839 | 290.0491087 | 747.5759761 | -1.363714418 | 1.40E-37 | 8.25E-36 | ABCC2 | protein_coding |
| ENSG00000169884 | 78.5660044 | 323.0351399 | -2.04413094 | 3.61E-37 | 2.07E-35 | WNT10B | protein_coding |
| ENSG00000117461 | 376.4639434 | 809.274886 | -1.104530646 | 5.16E-37 | 2.92E-35 | PIK3R3 | protein_coding |
| ENSG00000164045 | 881.3187894 | 410.8115017 | 1.101986217 | 9.60E-37 | 5.38E-35 | CDC25A | protein_coding |
| ENSG00000137834 | 316.9654817 | 716.4239102 | -1.175920424 | 6.09E-36 | 3.34E-34 | SMAD6 | protein_coding |
| ENSG00000226380 | 306.458184 | 67.54994931 | 2.180755043 | 9.08E-36 | 4.94E-34 | LINC-PINT | lincRNA |
| ENSG00000247095 | 67.11696569 | 307.5600459 | -2.201905404 | 1.79E-35 | 9.58E-34 | MIR210HG | lincRNA |
| ENSG00000154188 | 312.9283362 | 731.7671482 | -1.225568941 | 3.00E-35 | 1.57E-33 | ANGPT1 | protein_coding |
| ENSG00000135362 | 272.1405701 | 654.8405576 | -1.266280148 | 3.58E-35 | 1.86E-33 | PRR5L | protein_coding |
| ENSG00000114023 | 341.3631612 | 750.4916097 | -1.136234628 | 6.68E-35 | 3.41E-33 | FAM162A | protein_coding |
| ENSG00000075702 | 915.5004212 | 441.9177484 | 1.05183878 | 6.01E-34 | 2.94E-32 | WDR62 | protein_coding |
| ENSG00000058091 | 633.3398372 | 275.3849326 | 1.200682084 | 1.20E-33 | 5.75E-32 | CDK14 | protein_coding |
| ENSG00000126351 | 513.806328 | 1059.248959 | -1.045931846 | 1.91E-33 | 9.02E-32 | THRA | protein_coding |
| ENSG00000181588 | 1001.525422 | 468.9147879 | 1.093594397 | 3.37E-33 | 1.56E-31 | MEX3D | protein_coding |
| ENSG00000134986 | 292.0194681 | 692.9379709 | -1.247897049 | 7.05E-33 | 3.17E-31 | NREP | protein_coding |
| ENSG00000102384 | 887.251662 | 442.0133969 | 1.004459279 | 7.45E-33 | 3.32E-31 | CENPI | protein_coding |
| ENSG00000129195 | 752.665596 | 358.8911808 | 1.068175412 | 5.91E-32 | 2.57E-30 | PIMREG | protein_coding |
| ENSG00000164125 | 133.7190304 | 391.7363049 | -1.549474229 | 2.79E-31 | 1.18E-29 | FAM198B | protein_coding |
| ENSG00000178999 | 813.6170905 | 400.8427631 | 1.021646462 | 3.06E-31 | 1.29E-29 | AURKB | protein_coding |
| ENSG00000185947 | 481.8432602 | 185.6307778 | 1.374876069 | 1.07E-30 | 4.37E-29 | ZNF267 | protein_coding |
| ENSG00000137501 | 311.4159483 | 666.5027701 | -1.098844542 | 1.48E-30 | 6.02E-29 | SYTL2 | protein_coding |
| ENSG00000078018 | 354.9072973 | 762.6413129 | -1.102594483 | 3.53E-30 | 1.42E-28 | MAP2 | protein_coding |
| ENSG00000162063 | 736.0296209 | 351.8929016 | 1.066510384 | 5.37E-30 | 2.15E-28 | CCNF | protein_coding |
| ENSG00000100426 | 814.9912315 | 394.0628452 | 1.047528574 | 5.69E-30 | 2.27E-28 | ZBED4 | protein_coding |
| ENSG00000181773 | 307.010434 | 89.89395751 | 1.768851116 | 1.41E-29 | 5.57E-28 | GPR3 | protein_coding |
| ENSG00000090530 | 95.54115295 | 320.5507834 | -1.745171051 | 3.55E-29 | 1.37E-27 | P3H2 | protein_coding |
| ENSG00000139734 | 872.3277592 | 423.5140867 | 1.042952812 | 7.54E-29 | 2.87E-27 | DIAPH3 | protein_coding |
| ENSG00000049089 | 25.81719174 | 170.5375907 | -2.728058459 | 1.30E-28 | 4.90E-27 | COL9A2 | protein_coding |
| ENSG00000012171 | 314.6921507 | 717.6310999 | -1.188387828 | 1.37E-28 | 5.14E-27 | SEMA3B | protein_coding |
| ENSG00000153531 | 809.1371119 | 393.4906126 | 1.042579763 | 8.12E-28 | 2.96E-26 | ADPRHL1 | protein_coding |
| ENSG00000176971 | 97.85784588 | 313.6745234 | -1.681974159 | 1.13E-27 | 4.11E-26 | FIBIN | protein_coding |
| ENSG00000164761 | 69.01499421 | 257.913202 | -1.89733118 | 1.57E-27 | 5.65E-26 | TNFRSF11B | protein_coding |
| ENSG00000142798 | 262.8379398 | 572.0251472 | -1.121121711 | 2.80E-27 | 9.92E-26 | HSPG2 | protein_coding |
| ENSG00000099957 | 179.2934508 | 445.7961623 | -1.314730824 | 6.13E-27 | 2.14E-25 | P2RX6 | protein_coding |
| ENSG00000139354 | 662.7239724 | 318.7148252 | 1.057170175 | 1.51E-26 | 5.20E-25 | GAS2L3 | protein_coding |
| ENSG00000160888 | 766.0535193 | 358.4407324 | 1.093051025 | 2.97E-26 | 1.01E-24 | IER2 | protein_coding |
| ENSG00000131153 | 618.7034827 | 299.6845688 | 1.0469737 | 1.35E-25 | 4.39E-24 | GINS2 | protein_coding |
| ENSG00000145934 | 157.5990034 | 425.0954756 | -1.427963793 | 2.68E-25 | 8.55E-24 | TENM2 | protein_coding |
| ENSG00000132639 | 285.4357344 | 606.9445972 | -1.089716667 | 3.03E-25 | 9.66E-24 | SNAP25 | protein_coding |
| ENSG00000135472 | 89.81674685 | 280.4822243 | -1.641704672 | 3.63E-25 | 1.15E-23 | FAIM2 | protein_coding |
| ENSG00000222009 | 263.4146874 | 560.9119377 | -1.091495887 | 3.69E-25 | 1.16E-23 | BTBD19 | protein_coding |
| ENSG00000114315 | 146.3297764 | 23.05281545 | 2.667629434 | 3.90E-25 | 1.23E-23 | HES1 | protein_coding |
| ENSG00000088340 | 34.63236288 | 175.0474986 | -2.331614019 | 2.69E-24 | 8.18E-23 | FER1L4 | transcribed_unitary_pseudogene |
| ENSG00000138764 | 226.35978 | 497.8391959 | -1.135089712 | 4.80E-24 | 1.45E-22 | CCNG2 | protein_coding |
| ENSG00000085840 | 633.2674917 | 312.9998037 | 1.017360997 | 4.91E-24 | 1.48E-22 | ORC1 | protein_coding |
| ENSG00000151150 | 132.8836815 | 370.5767698 | -1.477651478 | 6.90E-24 | 2.06E-22 | ANK3 | protein_coding |
| ENSG00000117600 | 59.78954614 | 219.2679817 | -1.873283782 | 7.53E-24 | 2.24E-22 | PLPPR4 | protein_coding |
| ENSG00000118898 | 26.89218962 | 152.0495971 | -2.493314023 | 1.09E-23 | 3.22E-22 | PPL | protein_coding |
| ENSG00000181458 | 166.3488283 | 410.2239761 | -1.304548336 | 2.01E-23 | 5.83E-22 | TMEM45A | protein_coding |
| ENSG00000247092 | 259.0826803 | 83.27365851 | 1.63927348 | 2.95E-23 | 8.45E-22 | SNHG10 | antisense |
| ENSG00000162576 | 134.1889036 | 342.1868637 | -1.351826318 | 5.21E-23 | 1.47E-21 | MXRA8 | protein_coding |
| ENSG00000170379 | 78.42361482 | 269.9646238 | -1.781242556 | 1.40E-22 | 3.91E-21 | TCAF2 | protein_coding |
| ENSG00000167703 | 40.70720855 | 188.1438148 | -2.217341636 | 1.60E-22 | 4.43E-21 | SLC43A2 | protein_coding |
| ENSG00000185305 | 233.6536936 | 494.6756846 | -1.082493837 | 2.70E-22 | 7.43E-21 | ARL15 | protein_coding |
| ENSG00000171316 | 509.739505 | 235.6300179 | 1.111787306 | 2.85E-22 | 7.82E-21 | CHD7 | protein_coding |
| ENSG00000198796 | 223.442105 | 474.6451466 | -1.086867033 | 3.95E-22 | 1.08E-20 | ALPK2 | protein_coding |
| ENSG00000265415 | 372.5711091 | 159.9649345 | 1.219654441 | 2.40E-21 | 6.31E-20 | AC099850.3 | antisense |
| ENSG00000101255 | 410.5092497 | 151.5253963 | 1.442985805 | 2.50E-21 | 6.58E-20 | TRIB3 | protein_coding |
| ENSG00000077942 | 148.4865815 | 363.1010227 | -1.291915419 | 3.74E-21 | 9.74E-20 | FBLN1 | protein_coding |
| ENSG00000129521 | 43.552728 | 175.4766071 | -2.007923178 | 1.05E-20 | 2.66E-19 | EGLN3 | protein_coding |
| ENSG00000183486 | 77.55307958 | 232.8829601 | -1.590134952 | 5.00E-20 | 1.22E-18 | MX2 | protein_coding |
| ENSG00000204611 | 273.6295491 | 95.20508795 | 1.523058748 | 6.40E-20 | 1.54E-18 | ZNF616 | protein_coding |
| ENSG00000213390 | 438.4127714 | 212.5003969 | 1.044801737 | 7.59E-20 | 1.82E-18 | ARHGAP19 | protein_coding |
| ENSG00000099365 | 180.5345906 | 400.5011317 | -1.150478768 | 1.48E-19 | 3.51E-18 | STX1B | protein_coding |
| ENSG00000143409 | 217.5745276 | 452.3455219 | -1.053492453 | 1.57E-19 | 3.71E-18 | MINDY1 | protein_coding |
| ENSG00000100055 | 220.379023 | 465.4208213 | -1.078582725 | 1.64E-19 | 3.89E-18 | CYTH4 | protein_coding |
| ENSG00000105204 | 165.8212657 | 400.2690451 | -1.271371438 | 1.97E-19 | 4.65E-18 | DYRK1B | protein_coding |
| ENSG00000069424 | 99.45079144 | 266.9367101 | -1.42134624 | 3.61E-19 | 8.36E-18 | KCNAB2 | protein_coding |
| ENSG00000141526 | 173.2486115 | 382.3477015 | -1.144830859 | 7.27E-19 | 1.64E-17 | SLC16A3 | protein_coding |
| ENSG00000138100 | 54.25761328 | 190.979317 | -1.818167903 | 2.21E-18 | 4.84E-17 | TRIM54 | protein_coding |
| ENSG00000261040 | 124.1747418 | 329.2602761 | -1.403185108 | 3.18E-18 | 6.90E-17 | WFDC21P | transcribed_unitary_pseudogene |
| ENSG00000144821 | 215.7441686 | 435.3333779 | -1.013835803 | 8.14E-18 | 1.71E-16 | MYH15 | protein_coding |
| ENSG00000168016 | 134.2448102 | 317.9292131 | -1.244192939 | 1.28E-17 | 2.66E-16 | TRANK1 | protein_coding |
| ENSG00000189410 | 396.7131684 | 179.3941393 | 1.145357681 | 1.48E-17 | 3.06E-16 | SH2D5 | protein_coding |
| ENSG00000188015 | 219.7900449 | 442.2973889 | -1.009051353 | 1.51E-17 | 3.11E-16 | S100A3 | protein_coding |
| ENSG00000171860 | 153.1610936 | 337.2960209 | -1.141294508 | 3.48E-17 | 7.00E-16 | C3AR1 | protein_coding |
| ENSG00000061656 | 123.4529745 | 301.1564037 | -1.289123774 | 5.19E-17 | 1.03E-15 | SPAG4 | protein_coding |
| ENSG00000225756 | 165.1092667 | 364.897169 | -1.143966757 | 7.72E-17 | 1.51E-15 | DBH-AS1 | antisense |
| ENSG00000106003 | 89.10554416 | 239.5583589 | -1.422644882 | 8.02E-17 | 1.57E-15 | LFNG | protein_coding |
| ENSG00000171388 | 148.5189769 | 325.1009182 | -1.128819936 | 9.53E-17 | 1.86E-15 | APLN | protein_coding |
| ENSG00000110237 | 148.8604591 | 333.5198592 | -1.164586509 | 9.56E-17 | 1.86E-15 | ARHGEF17 | protein_coding |
| ENSG00000187908 | 116.892109 | 24.14597665 | 2.279906519 | 1.13E-16 | 2.19E-15 | DMBT1 | protein_coding |
| ENSG00000183763 | 381.7107318 | 185.2044874 | 1.043023687 | 1.14E-16 | 2.21E-15 | TRAIP | protein_coding |
| ENSG00000162645 | 166.5767145 | 355.945479 | -1.097252719 | 1.15E-16 | 2.22E-15 | GBP2 | protein_coding |
| ENSG00000110446 | 109.8721104 | 274.7766246 | -1.322522913 | 1.36E-16 | 2.62E-15 | SLC15A3 | protein_coding |
| ENSG00000197142 | 194.7855602 | 399.7969887 | -1.037820614 | 2.39E-16 | 4.54E-15 | ACSL5 | protein_coding |
| ENSG00000104043 | 16.45775615 | 99.99635491 | -2.597987635 | 5.01E-16 | 9.28E-15 | ATP8B4 | protein_coding |
| ENSG00000232774 | 22.45564611 | 110.3823778 | -2.289651742 | 7.13E-16 | 1.30E-14 | FLJ22447 | lincRNA |
| ENSG00000189120 | 188.3050113 | 377.4521944 | -1.004611459 | 1.23E-15 | 2.21E-14 | SP6 | protein_coding |
| ENSG00000100629 | 366.2876436 | 180.502681 | 1.021363772 | 1.25E-15 | 2.23E-14 | CEP128 | protein_coding |
| ENSG00000125965 | 75.24277953 | 205.0451589 | -1.443537844 | 1.28E-15 | 2.28E-14 | GDF5 | protein_coding |
| ENSG00000042980 | 119.4960579 | 283.213535 | -1.248124114 | 4.74E-15 | 8.07E-14 | ADAM28 | protein_coding |
| ENSG00000189184 | 77.44788561 | 218.8007703 | -1.498554414 | 9.12E-15 | 1.52E-13 | PCDH18 | protein_coding |
| ENSG00000183044 | 21.596545 | 104.2561239 | -2.267419971 | 1.23E-14 | 2.03E-13 | ABAT | protein_coding |
| ENSG00000135269 | 138.8675729 | 296.2348924 | -1.090331812 | 2.32E-14 | 3.75E-13 | TES | protein_coding |
| ENSG00000227471 | 35.03087691 | 129.4915643 | -1.884031123 | 3.04E-14 | 4.85E-13 | AKR1B15 | protein_coding |
| ENSG00000184368 | 35.02448403 | 127.298824 | -1.865128157 | 4.63E-14 | 7.27E-13 | MAP7D2 | protein_coding |
| ENSG00000236658 | 16.88035125 | 96.02109072 | -2.522806745 | 6.55E-14 | 1.01E-12 | AL161733.1 | antisense |
| ENSG00000152049 | 33.63343665 | 122.0757231 | -1.862577263 | 7.81E-14 | 1.20E-12 | KCNE4 | protein_coding |
| ENSG00000276600 | 152.0119456 | 308.7986461 | -1.021314323 | 8.94E-14 | 1.37E-12 | RAB7B | protein_coding |
| ENSG00000125675 | 123.9597291 | 267.8863271 | -1.110077304 | 1.21E-13 | 1.84E-12 | GRIA3 | protein_coding |
| ENSG00000064201 | 35.16262874 | 123.3810135 | -1.8099455 | 3.27E-13 | 4.73E-12 | TSPAN32 | protein_coding |
| ENSG00000128567 | 270.1821635 | 127.8860712 | 1.080881482 | 5.05E-13 | 7.22E-12 | PODXL | protein_coding |
| ENSG00000116774 | 166.0792605 | 333.2463983 | -1.00749373 | 6.01E-13 | 8.51E-12 | OLFML3 | protein_coding |
| ENSG00000241749 | 293.9279079 | 146.2363867 | 1.008055143 | 8.34E-13 | 1.17E-11 | RPSAP52 | transcribed_processed_pseudogene |
| ENSG00000156475 | 72.45732395 | 188.8428292 | -1.380315808 | 1.01E-12 | 1.41E-11 | PPP2R2B | protein_coding |
| ENSG00000167889 | 64.64443987 | 173.981401 | -1.425915276 | 1.16E-12 | 1.61E-11 | MGAT5B | protein_coding |
| ENSG00000149506 | 21.76516367 | 97.23165539 | -2.150046238 | 1.20E-12 | 1.66E-11 | ZP1 | protein_coding |
| ENSG00000164220 | 98.41489609 | 224.5696903 | -1.189628146 | 1.40E-12 | 1.92E-11 | F2RL2 | protein_coding |
| ENSG00000133056 | 79.88434096 | 194.0008569 | -1.282443992 | 1.54E-12 | 2.10E-11 | PIK3C2B | protein_coding |
| ENSG00000261371 | 44.48374357 | 140.3381017 | -1.665490618 | 2.19E-12 | 2.96E-11 | PECAM1 | protein_coding |
| ENSG00000275074 | 60.64696683 | 165.7203688 | -1.445989742 | 2.74E-12 | 3.66E-11 | NUDT18 | protein_coding |
| ENSG00000103522 | 55.06103421 | 156.173163 | -1.503180461 | 2.97E-12 | 3.96E-11 | IL21R | protein_coding |
| ENSG00000133321 | 84.56805156 | 201.2087255 | -1.247997227 | 3.11E-12 | 4.13E-11 | RARRES3 | protein_coding |
| ENSG00000284946 | 231.1286073 | 107.4441591 | 1.105006466 | 3.13E-12 | 4.16E-11 | AC068831.7 | protein_coding |
| ENSG00000274180 | 144.0433163 | 291.7228159 | -1.019868799 | 3.33E-12 | 4.41E-11 | NATD1 | protein_coding |
| ENSG00000149212 | 79.26880502 | 199.1002732 | -1.334201166 | 3.64E-12 | 4.80E-11 | SESN3 | protein_coding |
| ENSG00000204257 | 104.2998476 | 225.8294886 | -1.114243681 | 5.39E-12 | 6.97E-11 | HLA-DMA | protein_coding |
| ENSG00000132182 | 6.222744081 | 57.27149301 | -3.22333712 | 6.07E-12 | 7.82E-11 | NUP210 | protein_coding |
| ENSG00000155792 | 73.21996194 | 184.1695666 | -1.325301588 | 6.37E-12 | 8.20E-11 | DEPTOR | protein_coding |
| ENSG00000070808 | 51.74597033 | 143.3031548 | -1.474010754 | 9.33E-12 | 1.18E-10 | CAMK2A | protein_coding |
| ENSG00000136244 | 48.41266297 | 141.0583627 | -1.537523786 | 9.91E-12 | 1.25E-10 | IL6 | protein_coding |
| ENSG00000047936 | 31.06891908 | 106.6343449 | -1.777781388 | 1.33E-11 | 1.65E-10 | ROS1 | protein_coding |
| ENSG00000109819 | 93.85444755 | 214.0319958 | -1.185107948 | 1.36E-11 | 1.69E-10 | PPARGC1A | protein_coding |
| ENSG00000089127 | 62.42845494 | 160.2300388 | -1.360931288 | 1.37E-11 | 1.69E-10 | OAS1 | protein_coding |
| ENSG00000125637 | 79.44998296 | 187.1591435 | -1.235504153 | 1.42E-11 | 1.75E-10 | PSD4 | protein_coding |
| ENSG00000183508 | 89.2232828 | 210.6049972 | -1.239920932 | 1.48E-11 | 1.82E-10 | TENT5C | protein_coding |
| ENSG00000142619 | 100.0307976 | 218.3131922 | -1.127236013 | 1.49E-11 | 1.84E-10 | PADI3 | protein_coding |
| ENSG00000168386 | 113.0322254 | 240.4605884 | -1.088534902 | 2.20E-11 | 2.66E-10 | FILIP1L | protein_coding |
| ENSG00000132938 | 56.49095762 | 147.1881283 | -1.380615562 | 3.67E-11 | 4.34E-10 | MTUS2 | protein_coding |
| ENSG00000122862 | 157.1911886 | 314.5402675 | -1.000083157 | 4.33E-11 | 5.09E-10 | SRGN | protein_coding |
| ENSG00000261115 | 58.5674462 | 150.6608049 | -1.362206698 | 5.03E-11 | 5.87E-10 | TMEM178B | protein_coding |
| ENSG00000196247 | 259.7468314 | 126.1872343 | 1.039421341 | 5.74E-11 | 6.65E-10 | ZNF107 | protein_coding |
| ENSG00000164188 | 18.98003686 | 82.16862842 | -2.113660753 | 6.01E-11 | 6.95E-10 | RANBP3L | protein_coding |
| ENSG00000185745 | 60.94565691 | 155.2239171 | -1.344164496 | 7.90E-11 | 9.02E-10 | IFIT1 | protein_coding |
| ENSG00000139209 | 13.26235241 | 70.11891616 | -2.407809396 | 9.83E-11 | 1.12E-09 | SLC38A4 | protein_coding |
| ENSG00000118292 | 48.56872237 | 135.6054902 | -1.482935377 | 1.05E-10 | 1.18E-09 | C1orf54 | protein_coding |
| ENSG00000214530 | 77.31086604 | 178.2045428 | -1.204295205 | 1.10E-10 | 1.24E-09 | STARD10 | protein_coding |
| ENSG00000131470 | 237.3382515 | 115.3489661 | 1.042324684 | 1.44E-10 | 1.61E-09 | PSMC3IP | protein_coding |
| ENSG00000169271 | 18.65983017 | 85.07220792 | -2.204169464 | 1.56E-10 | 1.74E-09 | HSPB3 | protein_coding |
| ENSG00000100100 | 44.93705372 | 130.88105 | -1.543175719 | 1.56E-10 | 1.74E-09 | PIK3IP1 | protein_coding |
| ENSG00000260708 | 75.07719292 | 17.29239442 | 2.124818598 | 1.58E-10 | 1.76E-09 | AL118516.1 | antisense |
| ENSG00000158258 | 47.25487179 | 126.7754975 | -1.421998781 | 2.45E-10 | 2.69E-09 | CLSTN2 | protein_coding |
| ENSG00000148541 | 84.95984393 | 198.1354124 | -1.221067316 | 2.51E-10 | 2.75E-09 | FAM13C | protein_coding |
| ENSG00000125740 | 70.01984574 | 14.86567739 | 2.232846063 | 2.55E-10 | 2.80E-09 | FOSB | protein_coding |
| ENSG00000102057 | 70.20358813 | 167.3810542 | -1.251755461 | 2.70E-10 | 2.95E-09 | KCND1 | protein_coding |
| ENSG00000150636 | 57.64180065 | 161.6577195 | -1.485827015 | 3.23E-10 | 3.49E-09 | CCDC102B | protein_coding |
| ENSG00000162415 | 70.15631003 | 163.8003959 | -1.221820578 | 4.93E-10 | 5.25E-09 | ZSWIM5 | protein_coding |
| ENSG00000125898 | 212.95289 | 105.4307549 | 1.014809533 | 5.19E-10 | 5.51E-09 | FAM110A | protein_coding |
| ENSG00000107738 | 43.1813271 | 119.4489309 | -1.466399724 | 6.33E-10 | 6.64E-09 | VSIR | protein_coding |
| ENSG00000104081 | 65.64761098 | 153.6032728 | -1.225894044 | 7.69E-10 | 8.00E-09 | BMF | protein_coding |
| ENSG00000138395 | 38.06573528 | 116.5257217 | -1.605719677 | 7.76E-10 | 8.06E-09 | CDK15 | protein_coding |
| ENSG00000182903 | 145.1683978 | 59.46451172 | 1.288854269 | 8.22E-10 | 8.51E-09 | ZNF721 | protein_coding |
| ENSG00000166402 | 38.82645176 | 111.2949084 | -1.523780441 | 9.34E-10 | 9.63E-09 | TUB | protein_coding |
| ENSG00000077420 | 60.18222255 | 153.0806838 | -1.354558835 | 1.14E-09 | 1.17E-08 | APBB1IP | protein_coding |
| ENSG00000116299 | 104.6077215 | 218.3492625 | -1.064167335 | 1.26E-09 | 1.28E-08 | KIAA1324 | protein_coding |
| ENSG00000175155 | 113.0099124 | 227.4124458 | -1.011251189 | 1.28E-09 | 1.30E-08 | YPEL2 | protein_coding |
| ENSG00000109339 | 62.94830953 | 150.0030774 | -1.254017764 | 1.56E-09 | 1.57E-08 | MAPK10 | protein_coding |
| ENSG00000119943 | 109.7042881 | 229.8232254 | -1.064915596 | 1.68E-09 | 1.68E-08 | PYROXD2 | protein_coding |
| ENSG00000110881 | 43.21852272 | 115.6312971 | -1.417637744 | 1.82E-09 | 1.82E-08 | ASIC1 | protein_coding |
| ENSG00000163395 | 55.11806603 | 138.3325957 | -1.322599588 | 2.26E-09 | 2.23E-08 | IGFN1 | protein_coding |
| ENSG00000170271 | 72.72642412 | 163.3893935 | -1.169975601 | 2.34E-09 | 2.30E-08 | FAXDC2 | protein_coding |
| ENSG00000146374 | 34.7050737 | 101.2834026 | -1.542844231 | 2.44E-09 | 2.41E-08 | RSPO3 | protein_coding |
| ENSG00000088280 | 77.64507132 | 172.3074156 | -1.146967813 | 2.83E-09 | 2.76E-08 | ASAP3 | protein_coding |
| ENSG00000223749 | 64.55357326 | 150.7782083 | -1.221546645 | 2.88E-09 | 2.81E-08 | MIR503HG | lincRNA |
| ENSG00000158008 | 21.97938002 | 85.9729137 | -1.957364885 | 3.08E-09 | 3.00E-08 | EXTL1 | protein_coding |
| ENSG00000123454 | 92.12145037 | 187.1612681 | -1.023194109 | 3.54E-09 | 3.42E-08 | DBH | protein_coding |
| ENSG00000163823 | 60.82487162 | 144.0882592 | -1.247568682 | 3.77E-09 | 3.61E-08 | CCR1 | protein_coding |
| ENSG00000115255 | 106.8126156 | 220.9802112 | -1.046294755 | 3.94E-09 | 3.78E-08 | REEP6 | protein_coding |
| ENSG00000137491 | 53.20413201 | 133.398522 | -1.327661291 | 4.01E-09 | 3.84E-08 | SLCO2B1 | protein_coding |
| ENSG00000167103 | 61.83947686 | 146.4349375 | -1.245909756 | 4.35E-09 | 4.15E-08 | PIP5KL1 | protein_coding |
| ENSG00000204947 | 61.73932414 | 144.6538447 | -1.227173762 | 4.69E-09 | 4.46E-08 | ZNF425 | protein_coding |
| ENSG00000143320 | 68.29739863 | 157.2574101 | -1.200374397 | 4.88E-09 | 4.63E-08 | CRABP2 | protein_coding |
| ENSG00000021645 | 94.67332099 | 196.7536875 | -1.052927584 | 5.07E-09 | 4.80E-08 | NRXN3 | protein_coding |
| ENSG00000163376 | 160.7513593 | 72.07639038 | 1.156209025 | 5.93E-09 | 5.57E-08 | KBTBD8 | protein_coding |
| ENSG00000170961 | 89.64140652 | 28.16862831 | 1.677033617 | 8.74E-09 | 8.05E-08 | HAS2 | protein_coding |
| ENSG00000163328 | 72.48644627 | 156.7496499 | -1.112266414 | 1.04E-08 | 9.50E-08 | GPR155 | protein_coding |
| ENSG00000214381 | 162.797615 | 78.11701784 | 1.059743417 | 1.41E-08 | 1.27E-07 | LINC00488 | processed_transcript |
| ENSG00000123689 | 48.21525076 | 124.7002973 | -1.369003332 | 1.45E-08 | 1.31E-07 | G0S2 | protein_coding |
| ENSG00000123405 | 8.106532917 | 47.11117774 | -2.538854859 | 1.68E-08 | 1.51E-07 | NFE2 | protein_coding |
| ENSG00000172346 | 10.14078464 | 54.24972391 | -2.418124783 | 2.92E-08 | 2.56E-07 | CSDC2 | protein_coding |
| ENSG00000184363 | 68.73198314 | 147.8689926 | -1.104860777 | 3.89E-08 | 3.33E-07 | PKP3 | protein_coding |
| ENSG00000224897 | 74.45975007 | 156.1305817 | -1.067230552 | 6.22E-08 | 5.20E-07 | POT1-AS1 | antisense |
| ENSG00000167941 | 1.674613724 | 32.26221935 | -4.257045615 | 6.83E-08 | 5.69E-07 | SOST | protein_coding |
| ENSG00000099998 | 10.17798025 | 51.08094747 | -2.328561827 | 7.47E-08 | 6.18E-07 | GGT5 | protein_coding |
| ENSG00000253414 | 10.36004224 | 51.11175257 | -2.29290725 | 7.76E-08 | 6.41E-07 | AC124067.2 | sense_intronic |
| ENSG00000267100 | 157.5987623 | 76.99873182 | 1.031168398 | 9.86E-08 | 8.08E-07 | ILF3-DT | lincRNA |
| ENSG00000185338 | 88.70231766 | 181.7671876 | -1.034671441 | 1.10E-07 | 8.98E-07 | SOCS1 | protein_coding |
| ENSG00000253368 | 68.78845968 | 147.58292 | -1.096003743 | 1.17E-07 | 9.49E-07 | TRNP1 | protein_coding |
| ENSG00000173727 | 55.79711435 | 124.5192517 | -1.156697442 | 1.23E-07 | 9.92E-07 | AP000769.1 | transcribed_unprocessed_pseudogene |
| ENSG00000169891 | 19.77034326 | 68.38530338 | -1.795937165 | 1.61E-07 | 1.28E-06 | REPS2 | protein_coding |
| ENSG00000161381 | 69.87385423 | 148.9646935 | -1.09628142 | 1.67E-07 | 1.33E-06 | PLXDC1 | protein_coding |
| ENSG00000134042 | 16.44464161 | 59.64828289 | -1.868615599 | 2.32E-07 | 1.81E-06 | MRO | protein_coding |
| ENSG00000196782 | 68.26155466 | 149.4737481 | -1.124394218 | 2.35E-07 | 1.83E-06 | MAML3 | protein_coding |
| ENSG00000119922 | 78.53889127 | 159.2075871 | -1.022345819 | 2.60E-07 | 2.02E-06 | IFIT2 | protein_coding |
| ENSG00000145284 | 19.1727366 | 66.09210791 | -1.795812419 | 2.74E-07 | 2.12E-06 | SCD5 | protein_coding |
| ENSG00000117407 | 53.68679098 | 121.0410314 | -1.173207771 | 2.90E-07 | 2.24E-06 | ARTN | protein_coding |
| ENSG00000196154 | 40.94630234 | 103.59087 | -1.337636235 | 3.22E-07 | 2.48E-06 | S100A4 | protein_coding |
| ENSG00000205809 | 33.00670768 | 86.03337127 | -1.378400154 | 4.62E-07 | 3.46E-06 | KLRC2 | protein_coding |
| ENSG00000237424 | 109.6833267 | 48.1173255 | 1.185721954 | 5.36E-07 | 3.97E-06 | FOXD2-AS1 | lincRNA |
| ENSG00000134871 | 170.1159895 | 83.66784936 | 1.024442174 | 5.46E-07 | 4.04E-06 | COL4A2 | protein_coding |
| ENSG00000136842 | 49.3584735 | 112.6156234 | -1.191291699 | 5.59E-07 | 4.13E-06 | TMOD1 | protein_coding |
| ENSG00000181450 | 96.07332572 | 40.10693986 | 1.263968809 | 5.97E-07 | 4.40E-06 | ZNF678 | protein_coding |
| ENSG00000105246 | 29.8971293 | 81.71065358 | -1.44553376 | 6.86E-07 | 5.00E-06 | EBI3 | protein_coding |
| ENSG00000138135 | 21.85187308 | 65.87125044 | -1.598364232 | 9.06E-07 | 6.49E-06 | CH25H | protein_coding |
| ENSG00000115657 | 20.31708448 | 62.86698648 | -1.628544473 | 1.04E-06 | 7.39E-06 | ABCB6 | protein_coding |
| ENSG00000165899 | 55.68776318 | 122.3434597 | -1.137382255 | 1.17E-06 | 8.22E-06 | OTOGL | protein_coding |
| ENSG00000115461 | 12.46532436 | 48.64762678 | -1.95859483 | 1.17E-06 | 8.25E-06 | IGFBP5 | protein_coding |
| ENSG00000103313 | 10.26077356 | 44.22847384 | -2.115768585 | 1.21E-06 | 8.51E-06 | MEFV | protein_coding |
| ENSG00000169583 | 19.41351081 | 64.06217062 | -1.713833567 | 1.27E-06 | 8.89E-06 | CLIC3 | protein_coding |
| ENSG00000111907 | 33.7805387 | 85.89781772 | -1.350905965 | 1.30E-06 | 9.09E-06 | TPD52L1 | protein_coding |
| ENSG00000170412 | 12.46196353 | 48.18124613 | -1.948196991 | 1.40E-06 | 9.73E-06 | GPRC5C | protein_coding |
| ENSG00000167646 | 54.76939457 | 116.2168348 | -1.08841577 | 1.79E-06 | 1.22E-05 | DNAAF3 | protein_coding |
| ENSG00000272121 | 66.18291807 | 22.35301492 | 1.564093362 | 2.34E-06 | 1.57E-05 | AC006058.3 | lincRNA |
| ENSG00000130649 | 28.85541093 | 77.00390441 | -1.411125558 | 2.36E-06 | 1.59E-05 | CYP2E1 | protein_coding |
| ENSG00000184584 | 20.43875381 | 60.61147823 | -1.573494937 | 2.55E-06 | 1.70E-05 | TMEM173 | protein_coding |
| ENSG00000182389 | 20.81990842 | 62.21540358 | -1.572099108 | 2.62E-06 | 1.74E-05 | CACNB4 | protein_coding |
| ENSG00000174669 | 15.63921148 | 52.0117208 | -1.736471466 | 3.10E-06 | 2.03E-05 | SLC29A2 | protein_coding |
| ENSG00000266709 | 42.61587482 | 9.606598707 | 2.165570414 | 3.21E-06 | 2.10E-05 | MGC12916 | lincRNA |
| ENSG00000143412 | 64.44086127 | 129.5061147 | -1.007162333 | 3.52E-06 | 2.29E-05 | ANXA9 | protein_coding |
| ENSG00000018625 | 47.56723168 | 103.8215644 | -1.12514196 | 3.57E-06 | 2.32E-05 | ATP1A2 | protein_coding |
| ENSG00000115041 | 7.419411301 | 37.72700939 | -2.339994515 | 3.90E-06 | 2.51E-05 | KCNIP3 | protein_coding |
| ENSG00000283511 | 2.645301687 | 26.97589125 | -3.326221767 | 3.96E-06 | 2.55E-05 | AC137936.2 | transcribed_unitary_pseudogene |
| ENSG00000099282 | 3.018382998 | 25.66644424 | -3.078227134 | 4.74E-06 | 3.03E-05 | TSPAN15 | protein_coding |
| ENSG00000105516 | 37.15800442 | 87.27404751 | -1.231952941 | 4.90E-06 | 3.12E-05 | DBP | protein_coding |
| ENSG00000179044 | 13.71990744 | 49.29851615 | -1.855345804 | 4.93E-06 | 3.14E-05 | EXOC3L1 | protein_coding |
| ENSG00000167972 | 87.42005158 | 37.93844506 | 1.203612424 | 5.16E-06 | 3.28E-05 | ABCA3 | protein_coding |
| ENSG00000239332 | 53.7396656 | 111.1084117 | -1.05164376 | 5.44E-06 | 3.45E-05 | LINC01119 | lincRNA |
| ENSG00000257732 | 45.43707211 | 99.69730488 | -1.136918453 | 5.80E-06 | 3.65E-05 | AC089983.1 | antisense |
| ENSG00000149596 | 53.83389303 | 110.2335683 | -1.035647247 | 6.19E-06 | 3.89E-05 | JPH2 | protein_coding |
| ENSG00000260563 | 97.99127812 | 44.70672337 | 1.135161592 | 6.83E-06 | 4.27E-05 | AC132872.1 | lincRNA |
| ENSG00000171631 | 35.02751608 | 82.03067183 | -1.224600291 | 8.20E-06 | 5.05E-05 | P2RY6 | protein_coding |
| ENSG00000130518 | 44.19033582 | 101.4074098 | -1.193453962 | 8.34E-06 | 5.13E-05 | IQCN | protein_coding |
| ENSG00000117266 | 6.513032085 | 32.64999235 | -2.334763127 | 9.17E-06 | 5.60E-05 | CDK18 | protein_coding |
| ENSG00000173578 | 20.67448678 | 62.51870282 | -1.585479438 | 9.51E-06 | 5.79E-05 | XCR1 | protein_coding |
| ENSG00000198774 | 18.92716224 | 59.24439179 | -1.63400124 | 9.65E-06 | 5.87E-05 | RASSF9 | protein_coding |
| ENSG00000164400 | 9.333674097 | 38.32866514 | -2.025005007 | 9.97E-06 | 6.05E-05 | CSF2 | protein_coding |
| ENSG00000230615 | 33.58615855 | 79.0903285 | -1.236027685 | 1.02E-05 | 6.20E-05 | AL139220.2 | lincRNA |
| ENSG00000170579 | 6.435280022 | 31.49605111 | -2.291485395 | 1.04E-05 | 6.28E-05 | DLGAP1 | protein_coding |
| ENSG00000177453 | 24.74299022 | 66.29227562 | -1.414654762 | 1.18E-05 | 7.10E-05 | NIM1K | protein_coding |
| ENSG00000260630 | 29.96176682 | 73.02845439 | -1.290923881 | 1.22E-05 | 7.28E-05 | SNAI3-AS1 | antisense |
| ENSG00000165694 | 32.05897565 | 81.92693938 | -1.364874723 | 1.23E-05 | 7.34E-05 | FRMD7 | protein_coding |
| ENSG00000221852 | 10.70320486 | 43.44207495 | -2.037749566 | 1.27E-05 | 7.57E-05 | KRTAP1-5 | protein_coding |
| ENSG00000234537 | 17.00449737 | 54.37410211 | -1.664150683 | 2.08E-05 | 0.0001202 | AL354751.1 | unprocessed_pseudogene |
| ENSG00000168453 | 2.074808165 | 20.73195548 | -3.344498271 | 2.11E-05 | 0.0001215 | HR | protein_coding |
| ENSG00000185561 | 15.15622374 | 46.80543142 | -1.622338481 | 2.20E-05 | 0.0001264 | TLCD2 | protein_coding |
| ENSG00000187634 | 41.14203414 | 87.89694992 | -1.092899526 | 2.32E-05 | 0.0001329 | SAMD11 | protein_coding |
| ENSG00000143512 | 53.17636133 | 110.4850942 | -1.048644622 | 2.35E-05 | 0.0001349 | HHIPL2 | protein_coding |
| ENSG00000129422 | 5.781993194 | 28.90967173 | -2.32589407 | 2.36E-05 | 0.0001353 | MTUS1 | protein_coding |
| ENSG00000133055 | 5.302366276 | 28.8180866 | -2.421080678 | 2.86E-05 | 0.0001608 | MYBPH | protein_coding |
| ENSG00000115380 | 13.50233026 | 45.17910678 | -1.758172572 | 2.94E-05 | 0.0001652 | EFEMP1 | protein_coding |
| ENSG00000231574 | 11.65485299 | 42.62838262 | -1.858143337 | 3.06E-05 | 0.0001715 | LINC02015 | lincRNA |
| ENSG00000228358 | 15.0818325 | 46.36800971 | -1.613166912 | 3.18E-05 | 0.0001774 | LINC02263 | lincRNA |
| ENSG00000119915 | 40.06648098 | 87.35852127 | -1.120916983 | 3.35E-05 | 0.0001861 | ELOVL3 | protein_coding |
| ENSG00000261364 | 93.94988781 | 44.73013866 | 1.069363402 | 3.57E-05 | 0.0001974 | AC063923.2 | lincRNA |
| ENSG00000152779 | 34.41398933 | 82.5368643 | -1.269832718 | 3.76E-05 | 0.0002071 | SLC16A12 | protein_coding |
| ENSG00000277632 | 16.44240592 | 53.57200021 | -1.698444006 | 3.96E-05 | 0.0002174 | CCL3 | protein_coding |
| ENSG00000127152 | 91.0760424 | 43.9518231 | 1.05019325 | 4.25E-05 | 0.0002324 | BCL11B | protein_coding |
| ENSG00000161896 | 15.44483133 | 47.29180831 | -1.611597147 | 4.41E-05 | 0.0002402 | IP6K3 | protein_coding |
| ENSG00000178199 | 30.72608523 | 71.30324742 | -1.213289634 | 4.46E-05 | 0.000243 | ZC3H12D | protein_coding |
| ENSG00000179344 | 25.63793531 | 66.47530924 | -1.3710956 | 4.56E-05 | 0.0002484 | HLA-DQB1 | protein_coding |
| ENSG00000114841 | 37.76233273 | 81.4751091 | -1.105172562 | 4.70E-05 | 0.0002553 | DNAH1 | protein_coding |
| ENSG00000270605 | 12.04193289 | 41.3433669 | -1.792926779 | 5.11E-05 | 0.0002759 | AL353622.1 | antisense |
| ENSG00000196338 | 1.019646481 | 17.69730151 | -4.119556891 | 5.98E-05 | 0.0003195 | NLGN3 | protein_coding |
| ENSG00000151689 | 18.71294588 | 51.77174128 | -1.460078059 | 6.01E-05 | 0.000321 | INPP1 | protein_coding |
| ENSG00000236197 | 6.360888788 | 28.90653113 | -2.174513652 | 6.40E-05 | 0.0003399 | AC002429.2 | antisense |
| ENSG00000154928 | 34.81698933 | 80.00004101 | -1.204406533 | 6.55E-05 | 0.0003473 | EPHB1 | protein_coding |
| ENSG00000145569 | 86.39480858 | 42.43094043 | 1.030804702 | 7.53E-05 | 0.0003953 | OTULINL | protein_coding |
| ENSG00000163545 | 13.89188694 | 42.12389967 | -1.601665323 | 7.93E-05 | 0.0004139 | NUAK2 | protein_coding |
| ENSG00000215644 | 12.25278842 | 39.24364285 | -1.683756103 | 8.16E-05 | 0.000425 | GCGR | protein_coding |
| ENSG00000205403 | 19.87129235 | 53.37982323 | -1.421194474 | 8.36E-05 | 0.0004348 | CFI | protein_coding |
| ENSG00000091262 | 22.03561547 | 54.95691425 | -1.318720909 | 8.94E-05 | 0.0004637 | ABCC6 | protein_coding |
| ENSG00000285796 | 44.85458919 | 95.06172951 | -1.084706413 | 8.95E-05 | 0.0004638 | AL162458.1 | antisense |
| ENSG00000166924 | 19.76306632 | 52.32374832 | -1.399094164 | 9.28E-05 | 0.0004782 | NYAP1 | protein_coding |
| ENSG00000256732 | 3.747741475 | 22.46515318 | -2.588638449 | 9.35E-05 | 0.0004815 | AC006065.4 | lincRNA |
| ENSG00000123358 | 70.28693671 | 32.14550941 | 1.127946516 | 9.50E-05 | 0.0004891 | NR4A1 | protein_coding |
| ENSG00000174807 | 2.322859309 | 18.83383022 | -2.998985771 | 1.00E-04 | 0.0005121 | CD248 | protein_coding |
| ENSG00000244560 | 29.77578874 | 67.37347533 | -1.176651111 | 0.0001017 | 0.0005202 | AC004890.2 | transcribed_unprocessed_pseudogene |
| ENSG00000236790 | 10.50378346 | 36.88084646 | -1.813754057 | 0.0001032 | 0.0005273 | LINC00299 | lincRNA |
| ENSG00000162591 | 3.668308997 | 21.69727543 | -2.551042983 | 0.0001052 | 0.0005365 | MEGF6 | protein_coding |
| ENSG00000173432 | 34.60748544 | 73.2349033 | -1.083582835 | 0.0001107 | 0.0005624 | SAA1 | protein_coding |
| ENSG00000250091 | 31.48423725 | 68.69488383 | -1.126225158 | 0.0001139 | 0.0005771 | DNAH10OS | antisense |
| ENSG00000111319 | 8.609356863 | 34.10584578 | -1.9723427 | 0.0001217 | 0.0006118 | SCNN1A | protein_coding |
| ENSG00000158246 | 40.16798535 | 81.78016189 | -1.025424408 | 0.0001231 | 0.000618 | TENT5B | protein_coding |
| ENSG00000128284 | 47.92686967 | 96.41796306 | -1.004423869 | 0.0001347 | 0.0006722 | APOL3 | protein_coding |
| ENSG00000180066 | 14.46406088 | 41.77977261 | -1.526399678 | 0.000137 | 0.0006824 | C10orf91 | lincRNA |
| ENSG00000152454 | 74.24184411 | 35.60603869 | 1.059944633 | 0.00015 | 0.0007412 | ZNF256 | protein_coding |
| ENSG00000105499 | 22.14216108 | 53.93293888 | -1.288276515 | 0.0001669 | 0.0008166 | PLA2G4C | protein_coding |
| ENSG00000146411 | 15.84502577 | 43.17512445 | -1.44304387 | 0.0001709 | 0.0008347 | SLC2A12 | protein_coding |
| ENSG00000166106 | 7.931192593 | 30.02837283 | -1.937049197 | 0.0001729 | 0.0008432 | ADAMTS15 | protein_coding |
| ENSG00000172935 | 24.54053677 | 60.29884979 | -1.292975549 | 0.0001741 | 0.0008488 | MRGPRF | protein_coding |
| ENSG00000166407 | 13.62367082 | 42.39884089 | -1.642918236 | 0.0001743 | 0.0008499 | LMO1 | protein_coding |
| ENSG00000174502 | 9.171777088 | 34.12750752 | -1.897749158 | 0.0001757 | 0.0008558 | SLC26A9 | protein_coding |
| ENSG00000234362 | 9.698353335 | 33.03222171 | -1.759391896 | 0.0001785 | 0.0008676 | LINC01914 | lincRNA |
| ENSG00000186510 | 5.704241131 | 25.41704292 | -2.151220037 | 0.0001824 | 0.0008836 | CLCNKA | protein_coding |
| ENSG00000198133 | 29.92569635 | 68.15543919 | -1.183301718 | 0.0001865 | 0.0009011 | TMEM229B | protein_coding |
| ENSG00000157368 | 3.308671002 | 20.87134316 | -2.63497186 | 0.0001905 | 0.0009185 | IL34 | protein_coding |
| ENSG00000284906 | 71.15275948 | 33.94475234 | 1.071180253 | 0.0001961 | 0.0009421 | AC091057.6 | protein_coding |
| ENSG00000239713 | 27.73649578 | 63.12493026 | -1.188683153 | 0.000197 | 0.0009457 | APOBEC3G | protein_coding |
| ENSG00000071909 | 25.86918231 | 59.77085895 | -1.202849539 | 0.000203 | 0.0009719 | MYO3B | protein_coding |
| ENSG00000124116 | 37.61859151 | 80.50239934 | -1.091743453 | 0.0002048 | 0.0009788 | WFDC3 | protein_coding |
| ENSG00000265190 | 26.51439584 | 60.18103124 | -1.184389 | 0.0002252 | 0.0010681 | ANXA8 | protein_coding |
| ENSG00000110665 | 15.10894563 | 42.32508337 | -1.479287601 | 0.0002663 | 0.0012472 | C11orf21 | protein_coding |
| ENSG00000231890 | 23.09245757 | 54.00628132 | -1.226920684 | 0.0002694 | 0.0012598 | DARS-AS1 | antisense |
| ENSG00000215853 | 2.035932134 | 16.43648722 | -3.017837884 | 0.0002986 | 0.0013832 | RPTN | protein_coding |
| ENSG00000138378 | 15.81119098 | 42.28670263 | -1.416812353 | 0.0003114 | 0.0014377 | STAT4 | protein_coding |
| ENSG00000181444 | 5.383479167 | 23.89131012 | -2.146591155 | 0.0003561 | 0.0016291 | ZNF467 | protein_coding |
| ENSG00000122367 | 1.016285652 | 13.99993331 | -3.784172657 | 0.0003601 | 0.0016458 | LDB3 | protein_coding |
| ENSG00000135744 | 19.26528362 | 46.73938618 | -1.277482877 | 0.0003745 | 0.001706 | AGT | protein_coding |
| ENSG00000083067 | 26.3099332 | 59.21113962 | -1.165166333 | 0.0004299 | 0.0019293 | TRPM3 | protein_coding |
| ENSG00000277945 | 38.35018567 | 13.97268389 | 1.456761597 | 0.0004356 | 0.0019542 | AC107308.1 | sense_intronic |
| ENSG00000232901 | 19.93592987 | 3.228540478 | 2.618360166 | 0.0004449 | 0.0019892 | CYCSP10 | processed_pseudogene |
| ENSG00000129170 | 5.628169483 | 24.0391036 | -2.076187037 | 0.0004483 | 0.0020008 | CSRP3 | protein_coding |
| ENSG00000268403 | 34.86057783 | 12.32437504 | 1.499964191 | 0.0004595 | 0.0020447 | AC132192.2 | antisense |
| ENSG00000262691 | 32.74050075 | 68.72171816 | -1.072900166 | 0.000461 | 0.0020497 | AC040160.1 | processed_transcript |
| ENSG00000142583 | 10.36899958 | 32.92008345 | -1.683559859 | 0.0004625 | 0.0020553 | SLC2A5 | protein_coding |
| ENSG00000101977 | 14.09602081 | 39.05086496 | -1.466011262 | 0.0004652 | 0.0020645 | MCF2 | protein_coding |
| ENSG00000152822 | 9.618920858 | 32.51203576 | -1.745197358 | 0.0005029 | 0.0022196 | GRM1 | protein_coding |
| ENSG00000237975 | 29.16305836 | 62.62903895 | -1.103819747 | 0.0005137 | 0.0022617 | FLG-AS1 | antisense |
| ENSG00000131386 | 2.76529061 | 17.43492269 | -2.671808425 | 0.0005316 | 0.002332 | GALNT15 | protein_coding |
| ENSG00000253819 | 0.690482445 | 13.03045677 | -4.260896888 | 0.0005518 | 0.0024135 | LINC01151 | lincRNA |
| ENSG00000121871 | 7.167999328 | 25.9917277 | -1.864594701 | 0.0005871 | 0.0025533 | SLITRK3 | protein_coding |
| ENSG00000214975 | 61.68005663 | 29.71463579 | 1.052612921 | 0.0006159 | 0.0026615 | PPIAP29 | processed_pseudogene |
| ENSG00000181418 | 16.11379716 | 41.91491108 | -1.38733226 | 0.0006237 | 0.0026918 | DDN | protein_coding |
| ENSG00000168961 | 0.654967243 | 12.9161939 | -4.263603576 | 0.0006379 | 0.0027462 | LGALS9 | protein_coding |
| ENSG00000186377 | 26.564706 | 57.99826451 | -1.124897456 | 0.0006512 | 0.0027976 | CYP4X1 | protein_coding |
| ENSG00000110195 | 14.28535972 | 37.78751098 | -1.40861375 | 0.0006866 | 0.0029374 | FOLR1 | protein_coding |
| ENSG00000114737 | 4.97320224 | 21.91143661 | -2.12473632 | 0.000697 | 0.0029756 | CISH | protein_coding |
| ENSG00000175309 | 36.30697661 | 13.42067685 | 1.444593156 | 0.0007389 | 0.0031401 | PHYKPL | protein_coding |
| ENSG00000128849 | 5.741436748 | 22.38238894 | -1.96118549 | 0.0007475 | 0.0031705 | CGNL1 | protein_coding |
| ENSG00000196220 | 1.382645305 | 13.48145688 | -3.303742324 | 0.0007532 | 0.0031923 | SRGAP3 | protein_coding |
| ENSG00000168309 | 13.84965008 | 36.35507283 | -1.390684045 | 0.0007763 | 0.0032718 | FAM107A | protein_coding |
| ENSG00000148795 | 9.06410634 | 29.9399283 | -1.74376497 | 0.0008028 | 0.0033678 | CYP17A1 | protein_coding |
| ENSG00000227496 | 24.87865815 | 54.69869203 | -1.139695427 | 0.0008041 | 0.0033718 | AC099066.2 | antisense |
| ENSG00000251281 | 44.60452885 | 19.12973965 | 1.221706294 | 0.0008124 | 0.0034022 | AC034223.2 | lincRNA |
| ENSG00000250629 | 0.651606414 | 12.76807795 | -4.245727599 | 0.0008557 | 0.0035642 | AC091435.2 | antisense |
| ENSG00000144285 | 2.513878638 | 16.89903374 | -2.800748796 | 0.000857 | 0.0035686 | SCN1A | protein_coding |
| ENSG00000240583 | 17.0788886 | 43.56391345 | -1.340843015 | 0.0008577 | 0.0035698 | AQP1 | protein_coding |
| ENSG00000267073 | 33.9486021 | 69.90507744 | -1.040492898 | 0.0008651 | 0.0035983 | AC005256.1 | lincRNA |
| ENSG00000257524 | 11.08099864 | 32.32928058 | -1.540300495 | 0.0008687 | 0.0036094 | AL157935.2 | protein_coding |
| ENSG00000153253 | 32.11120732 | 72.67703013 | -1.16891571 | 0.0008769 | 0.0036381 | SCN3A | protein_coding |
| ENSG00000233381 | 12.61746766 | 34.83987045 | -1.469366745 | 0.0008818 | 0.003656 | AK4P3 | processed_pseudogene |
| ENSG00000040608 | 4.109059884 | 18.84325202 | -2.206933531 | 0.0008852 | 0.003669 | RTN4R | protein_coding |
| ENSG00000103154 | 19.16265412 | 45.05754671 | -1.241184954 | 0.0008922 | 0.0036964 | NECAB2 | protein_coding |
| ENSG00000168928 | 1.708448512 | 13.7903438 | -3.026190883 | 0.0009104 | 0.0037645 | CTRB2 | protein_coding |
| ENSG00000198406 | 63.12285349 | 31.0492076 | 1.018603747 | 0.0009133 | 0.0037727 | BZW1P2 | processed_pseudogene |
| ENSG00000108176 | 19.26192279 | 45.87608917 | -1.25251708 | 0.0009211 | 0.0037997 | DNAJC12 | protein_coding |
| ENSG00000273521 | 5.95397269 | 23.54261138 | -1.962910191 | 0.0009456 | 0.0038966 | AL162274.1 | antisense |
| ENSG00000153982 | 28.58752359 | 58.89398353 | -1.047261721 | 0.0009555 | 0.0039346 | GDPD1 | protein_coding |
| ENSG00000170099 | 12.03297555 | 34.11282051 | -1.494069524 | 0.0010266 | 0.0042036 | SERPINA6 | protein_coding |
| ENSG00000244486 | 2.653703759 | 15.85510615 | -2.561044137 | 0.0010294 | 0.0042117 | SCARF2 | protein_coding |
| ENSG00000241644 | 3.710545858 | 18.68603674 | -2.331585936 | 0.0010617 | 0.0043317 | INMT | protein_coding |
| ENSG00000277758 | 16.32073658 | 42.94415165 | -1.3850847 | 0.0011 | 0.0044685 | FO681492.1 | protein_coding |
| ENSG00000278771 | 36.02845151 | 75.32294429 | -1.058796738 | 0.0011429 | 0.0046274 | RN7SL3 | misc_RNA |
| ENSG00000074660 | 6.433599608 | 23.55101719 | -1.86969976 | 0.0011446 | 0.0046326 | SCARF1 | protein_coding |
| ENSG00000196358 | 21.79092516 | 50.49998149 | -1.216867212 | 0.0011571 | 0.0046757 | NTNG2 | protein_coding |
| ENSG00000274213 | 56.87211223 | 24.71825839 | 1.198318486 | 0.0012248 | 0.0049294 | AC015912.3 | lincRNA |
| ENSG00000125864 | 24.23512503 | 52.63536076 | -1.117206479 | 0.0012428 | 0.0049956 | BFSP1 | protein_coding |
| ENSG00000128610 | 23.13637485 | 51.06880015 | -1.146911831 | 0.001243 | 0.0049956 | FEZF1 | protein_coding |
| ENSG00000280832 | 30.2014159 | 61.85275538 | -1.036465408 | 0.0012596 | 0.0050564 | GSEC | antisense |
| ENSG00000179921 | 12.64738634 | 1.285338188 | 3.294073756 | 0.0013043 | 0.0052175 | GPBAR1 | protein_coding |
| ENSG00000223511 | 27.40901216 | 55.81425253 | -1.025768049 | 0.0013278 | 0.0053039 | AL683807.1 | lincRNA |
| ENSG00000235578 | 20.18398101 | 45.61440388 | -1.181758033 | 0.0013419 | 0.0053526 | AC007731.3 | lincRNA |
| ENSG00000271447 | 16.988022 | 40.89093571 | -1.269723916 | 0.0013788 | 0.0054932 | MMP28 | protein_coding |
| ENSG00000130203 | 28.78325539 | 9.500741651 | 1.60581615 | 0.0013816 | 0.0055031 | APOE | protein_coding |
| ENSG00000184350 | 18.35330789 | 3.892685782 | 2.24185435 | 0.0013823 | 0.0055046 | MRGPRE | protein_coding |
| ENSG00000265735 | 2.979506966 | 17.75078434 | -2.553138598 | 0.0013859 | 0.0055174 | RN7SL5P | misc_RNA |
| ENSG00000204314 | 10.7163194 | 30.6233323 | -1.509525164 | 0.0014345 | 0.0056872 | PRRT1 | protein_coding |
| ENSG00000165879 | 16.09475733 | 39.59201912 | -1.292229078 | 0.0014628 | 0.0057897 | FRAT1 | protein_coding |
| ENSG00000205898 | 15.92109742 | 39.66707108 | -1.313961457 | 0.0015246 | 0.0060105 | AC019155.1 | processed_pseudogene |
| ENSG00000229855 | 0.690482445 | 10.75254922 | -3.983119844 | 0.0015294 | 0.0060263 | AC008568.1 | lincRNA |
| ENSG00000138131 | 15.63585066 | 38.06416172 | -1.285501456 | 0.0015369 | 0.0060548 | LOXL4 | protein_coding |
| ENSG00000188385 | 9.892733493 | 28.73319776 | -1.541746138 | 0.0015477 | 0.0060902 | JAKMIP3 | protein_coding |
| ENSG00000241935 | 10.90733873 | 30.61349541 | -1.492572717 | 0.0016454 | 0.00644 | HOGA1 | protein_coding |
| ENSG00000103196 | 0.653286828 | 10.67606619 | -3.990029443 | 0.0018496 | 0.0071582 | CRISPLD2 | protein_coding |
| ENSG00000081985 | 24.35007271 | 7.804215171 | 1.643694719 | 0.0019383 | 0.0074657 | IL12RB2 | protein_coding |
| ENSG00000188338 | 2.321178895 | 14.31681095 | -2.605261206 | 0.0019733 | 0.0075903 | SLC38A3 | protein_coding |
| ENSG00000280417 | 13.98107313 | 2.269501737 | 2.627766265 | 0.0019975 | 0.0076782 | AC096887.2 | TEC |
| ENSG00000176222 | 26.23082949 | 54.81336998 | -1.067150457 | 0.0021075 | 0.0080663 | ZNF404 | protein_coding |
| ENSG00000106927 | 3.01166134 | 15.56233732 | -2.359786387 | 0.0021425 | 0.0081852 | AMBP | protein_coding |
| ENSG00000205129 | 7.812884084 | 24.44401069 | -1.646689159 | 0.0023185 | 0.0087823 | C4orf47 | protein_coding |
| ENSG00000260448 | 16.47175474 | 39.19653383 | -1.260140107 | 0.0023251 | 0.0087994 | LCMT1-AS1 | bidirectional_promoter_lncRNA |
| ENSG00000196502 | 17.29142455 | 40.90248212 | -1.230631759 | 0.0023458 | 0.0088718 | SULT1A1 | protein_coding |
| ENSG00000144596 | 3.057259029 | 15.27270909 | -2.323319332 | 0.0023952 | 0.0090387 | GRIP2 | protein_coding |
| ENSG00000064989 | 9.853857461 | 27.3105965 | -1.472795501 | 0.0025127 | 0.009442 | CALCRL | protein_coding |
| ENSG00000124140 | 12.21727322 | 31.67455708 | -1.377689727 | 0.0025202 | 0.0094661 | SLC12A5 | protein_coding |
| ENSG00000198520 | 9.566930286 | 27.60752193 | -1.535250786 | 0.0025648 | 0.0096058 | ARMH1 | protein_coding |
| ENSG00000159339 | 2.034251719 | 12.61400327 | -2.635237842 | 0.0025712 | 0.0096276 | PADI4 | protein_coding |
| ENSG00000225880 | 32.41436877 | 12.50671513 | 1.384239174 | 0.0025737 | 0.0096348 | LINC00115 | lincRNA |
| ENSG00000164056 | 8.54560339 | 26.22644201 | -1.621365883 | 0.002577 | 0.0096452 | SPRY1 | protein_coding |
| ENSG00000275484 | 15.20485348 | 2.215002909 | 2.764646178 | 0.0026162 | 0.0097682 | AP003419.3 | lincRNA |
| ENSG00000144452 | 1.637418107 | 12.89106909 | -2.94788301 | 0.0026573 | 0.0098952 | ABCA12 | protein_coding |
| ENSG00000244675 | 9.441900119 | 27.77628362 | -1.551829963 | 0.0026964 | 0.0100228 | AC108676.1 | sense_overlapping |
| ENSG00000072952 | 13.84965008 | 33.37006756 | -1.267878368 | 0.0027229 | 0.010102 | MRVI1 | protein_coding |
| ENSG00000006377 | 9.524693425 | 26.75475533 | -1.496159859 | 0.0027578 | 0.0102202 | DLX6 | protein_coding |
| ENSG00000175538 | 1.303212828 | 11.61515271 | -3.119808495 | 0.0028612 | 0.0105618 | KCNE3 | protein_coding |
| ENSG00000259345 | 6.805000504 | 22.46727779 | -1.725846478 | 0.0029281 | 0.01079 | AC013652.1 | lincRNA |
| ENSG00000182931 | 1.711809341 | 11.99281038 | -2.819928491 | 0.0029527 | 0.0108711 | WFDC10B | protein_coding |
| ENSG00000230699 | 6.970258342 | 22.71182899 | -1.689556057 | 0.0029937 | 0.0110125 | AL645608.2 | lincRNA |
| ENSG00000137843 | 5.381798753 | 19.34602544 | -1.843134656 | 0.0030899 | 0.0113324 | PAK6 | protein_coding |
| ENSG00000231776 | 7.529317738 | 24.13068873 | -1.690733918 | 0.0031045 | 0.0113702 | LINC01611 | lincRNA |
| ENSG00000141505 | 20.41467274 | 44.52479836 | -1.119969088 | 0.0031402 | 0.0114941 | ASGR1 | protein_coding |
| ENSG00000102935 | 5.856384428 | 20.02799836 | -1.789201952 | 0.003286 | 0.0119821 | ZNF423 | protein_coding |
| ENSG00000151320 | 10.54602032 | 28.05163993 | -1.417366871 | 0.0032863 | 0.0119821 | AKAP6 | protein_coding |
| ENSG00000173641 | 6.474156054 | 21.47997364 | -1.734215977 | 0.0033078 | 0.0120474 | HSPB7 | protein_coding |
| ENSG00000197558 | 5.415633541 | 18.85793903 | -1.79924254 | 0.0034626 | 0.0125655 | SSPO | protein_coding |
| ENSG00000258711 | 2.685858133 | 13.87837324 | -2.364034894 | 0.0034712 | 0.0125882 | AL358334.2 | lincRNA |
| ENSG00000170153 | 11.16379195 | 29.84917334 | -1.422205387 | 0.0034727 | 0.0125882 | RNF150 | protein_coding |
| ENSG00000186765 | 15.96669511 | 36.33096402 | -1.188718819 | 0.0035071 | 0.0126968 | FSCN2 | protein_coding |
| ENSG00000260257 | 56.6891662 | 27.38537002 | 1.050845488 | 0.0035675 | 0.0128963 | AL035071.1 | lincRNA |
| ENSG00000228261 | 13.27075449 | 2.552848765 | 2.361671559 | 0.0036042 | 0.0130039 | AL162742.1 | lincRNA |
| ENSG00000154451 | 0.979090035 | 9.952156846 | -3.310132326 | 0.0036945 | 0.01331 | GBP5 | protein_coding |
| ENSG00000172824 | 19.22136635 | 41.40798106 | -1.102890551 | 0.0037057 | 0.0133416 | CES4A | protein_coding |
| ENSG00000165181 | 5.785354023 | 19.95110024 | -1.787415749 | 0.0037636 | 0.0135215 | C9orf84 | protein_coding |
| ENSG00000280109 | 36.23010859 | 15.61683615 | 1.212277138 | 0.0038491 | 0.0137993 | PLAC4 | antisense |
| ENSG00000140090 | 0.690482445 | 9.080131555 | -3.739888532 | 0.0039042 | 0.0139733 | SLC24A4 | protein_coding |
| ENSG00000258583 | 12.50083956 | 32.61290605 | -1.377501003 | 0.0039133 | 0.0139998 | LINC01500 | lincRNA |
| ENSG00000260190 | 11.47783225 | 1.666136465 | 2.809772225 | 0.0039358 | 0.0140674 | AL807752.5 | sense_overlapping |
| ENSG00000257058 | 13.30626969 | 2.064762352 | 2.734694366 | 0.0039488 | 0.0141 | AP001363.2 | antisense |
| ENSG00000128606 | 2.321178895 | 13.03987857 | -2.470238918 | 0.0039718 | 0.0141733 | LRRC17 | protein_coding |
| ENSG00000260823 | 20.57521811 | 6.533563993 | 1.661620469 | 0.0039995 | 0.014254 | AC026461.3 | lincRNA |
| ENSG00000269388 | 15.12574978 | 35.50189115 | -1.224912957 | 0.0040039 | 0.0142667 | AC018755.3 | transcribed_processed_pseudogene |
| ENSG00000182359 | 11.8086767 | 29.46481938 | -1.317698466 | 0.0040401 | 0.0143869 | KBTBD3 | protein_coding |
| ENSG00000272977 | 21.4326388 | 44.60613152 | -1.049321064 | 0.0042095 | 0.0149396 | AL008721.2 | sense_intronic |
| ENSG00000085465 | 34.65947602 | 14.42409909 | 1.259394841 | 0.004215 | 0.0149561 | OVGP1 | protein_coding |
| ENSG00000255468 | 15.63753107 | 36.53681202 | -1.228237415 | 0.0043299 | 0.0153123 | AP001107.9 | antisense |
| ENSG00000271828 | 10.79743229 | 1.662995863 | 2.723609046 | 0.004461 | 0.0157299 | AC008937.3 | antisense |
| ENSG00000265096 | 5.017119514 | 18.13088909 | -1.844957507 | 0.0044761 | 0.0157735 | C1QTNF1-AS1 | antisense |
| ENSG00000111087 | 24.40126692 | 50.40738036 | -1.054476671 | 0.004481 | 0.0157845 | GLI1 | protein_coding |
| ENSG00000261051 | 21.01764941 | 42.8372786 | -1.02819798 | 0.0045329 | 0.0159337 | AC107021.2 | sense_overlapping |
| ENSG00000136541 | 3.58887652 | 16.77077739 | -2.192882044 | 0.0045593 | 0.0160199 | ERMN | protein_coding |
| ENSG00000162511 | 18.66823224 | 41.44922395 | -1.163847969 | 0.0045739 | 0.0160678 | LAPTM5 | protein_coding |
| ENSG00000250320 | 14.29712262 | 33.51887704 | -1.231936279 | 0.0045812 | 0.016087 | AC113383.1 | antisense |
| ENSG00000154678 | 2.437806989 | 12.72300093 | -2.407457399 | 0.0045944 | 0.0161267 | PDE1C | protein_coding |
| ENSG00000280184 | 43.44841808 | 21.05095773 | 1.043284169 | 0.0047253 | 0.0165382 | AL023806.3 | TEC |
| ENSG00000179300 | 8.758139331 | 24.9101129 | -1.505686169 | 0.0047329 | 0.0165578 | RTL3 | protein_coding |
| ENSG00000136514 | 11.87970711 | 30.29389225 | -1.354701357 | 0.0047831 | 0.0167161 | RTP4 | protein_coding |
| ENSG00000138400 | 6.717165955 | 21.06666074 | -1.643713206 | 0.0048856 | 0.0170286 | MDH1B | protein_coding |
| ENSG00000172543 | 18.40114127 | 39.46791937 | -1.103236716 | 0.0048946 | 0.0170498 | CTSW | protein_coding |
| ENSG00000182308 | 25.16726574 | 9.071725748 | 1.469463404 | 0.0049372 | 0.0171811 | DCAF4L1 | protein_coding |
| ENSG00000142408 | 16.16578773 | 36.15490513 | -1.159924315 | 0.0049905 | 0.0173444 | CACNG8 | protein_coding |
| ENSG00000072163 | 1.706768098 | 11.42611633 | -2.749204258 | 0.0050033 | 0.0173852 | LIMS2 | protein_coding |
| ENSG00000223764 | 6.624618936 | 21.03313012 | -1.689024096 | 0.0050151 | 0.017412 | LINC02593 | lincRNA |
| ENSG00000279124 | 16.17923105 | 35.69092753 | -1.138586472 | 0.0050786 | 0.0175965 | AL356585.2 | unprocessed_pseudogene |
| ENSG00000188747 | 13.26739366 | 31.30216461 | -1.243948546 | 0.0051035 | 0.0176646 | NOXA1 | protein_coding |
| ENSG00000185669 | 4.76906837 | 18.16756031 | -1.93438322 | 0.0051099 | 0.0176832 | SNAI3 | protein_coding |
| ENSG00000231789 | 4.79954233 | 17.12261673 | -1.846700665 | 0.0051398 | 0.0177831 | PIK3CD-AS2 | antisense |
| ENSG00000258659 | 1.711809341 | 10.97825682 | -2.69339852 | 0.0052021 | 0.0179619 | TRIM34 | protein_coding |
| ENSG00000235036 | 17.23271232 | 4.822350502 | 1.830992824 | 0.0052434 | 0.0180676 | AL035456.1 | processed_pseudogene |
| ENSG00000251615 | 3.702143786 | 15.74296789 | -2.086937493 | 0.0052504 | 0.0180839 | AC104825.1 | lincRNA |
| ENSG00000168772 | 12.41972667 | 30.55169938 | -1.292452001 | 0.0052702 | 0.0181415 | CXXC4 | protein_coding |
| ENSG00000241769 | 10.11758761 | 29.06688702 | -1.53853217 | 0.0053326 | 0.0183303 | LINC00893 | antisense |
| ENSG00000197943 | 2.689218962 | 12.94344332 | -2.261920002 | 0.0054628 | 0.018717 | PLCG2 | protein_coding |
| ENSG00000162687 | 23.2442721 | 47.49682614 | -1.027285757 | 0.0054628 | 0.018717 | KCNT2 | protein_coding |
| ENSG00000163431 | 11.84083108 | 30.12684008 | -1.347565527 | 0.005819 | 0.019818 | LMOD1 | protein_coding |
| ENSG00000269069 | 15.02312027 | 33.93533054 | -1.179192214 | 0.005964 | 0.0202186 | AC007842.1 | processed_pseudogene |
| ENSG00000253948 | 8.643191652 | 24.10616484 | -1.46558299 | 0.0059681 | 0.0202232 | AC104986.2 | lincRNA |
| ENSG00000101306 | 26.36616865 | 10.15232455 | 1.378901155 | 0.006092 | 0.0205732 | MYLK2 | protein_coding |
| ENSG00000183054 | 24.05082736 | 8.751707502 | 1.462722628 | 0.0062279 | 0.0209984 | RGPD6 | protein_coding |
| ENSG00000168477 | 16.78355936 | 36.50702291 | -1.113963346 | 0.0062576 | 0.0210862 | TNXB | protein_coding |
| ENSG00000271857 | 21.29113327 | 42.98964376 | -1.014246089 | 0.0062875 | 0.0211658 | AL096865.1 | antisense |
| ENSG00000130303 | 7.959430864 | 23.78129647 | -1.568438308 | 0.0063062 | 0.021216 | BST2 | protein_coding |
| ENSG00000134339 | 6.869309252 | 21.1850802 | -1.633730808 | 0.0063369 | 0.0212952 | SAA2 | protein_coding |
| ENSG00000269974 | 39.91825379 | 19.2198937 | 1.057278326 | 0.0063374 | 0.0212952 | AC091057.4 | lincRNA |
| ENSG00000258317 | 17.78841088 | 4.992128191 | 1.848130389 | 0.0063597 | 0.0213493 | AC034102.6 | antisense |
| ENSG00000169129 | 1.632376864 | 10.86611856 | -2.706032415 | 0.0064579 | 0.0216506 | AFAP1L2 | protein_coding |
| ENSG00000168925 | 3.491288259 | 15.56335332 | -2.179712368 | 0.0065389 | 0.0219077 | CTRB1 | protein_coding |
| ENSG00000156427 | 32.41436877 | 13.56634572 | 1.269177535 | 0.0066217 | 0.0221498 | FGF18 | protein_coding |
| ENSG00000234500 | 17.55179387 | 37.36163567 | -1.090982998 | 0.0066272 | 0.0221639 | AC008267.3 | unprocessed_pseudogene |
| ENSG00000156675 | 6.759402815 | 19.97978073 | -1.565052751 | 0.0069245 | 0.0230534 | RAB11FIP1 | protein_coding |
| ENSG00000265982 | 31.81395656 | 13.70716448 | 1.214544911 | 0.007006 | 0.0233065 | AC103810.3 | unprocessed_pseudogene |
| ENSG00000130997 | 15.66632461 | 34.22638985 | -1.133387402 | 0.0070277 | 0.0233697 | POLN | protein_coding |
| ENSG00000227398 | 38.92404003 | 19.15698906 | 1.023359964 | 0.0071246 | 0.0236453 | KIF9-AS1 | antisense |
| ENSG00000147041 | 8.067656886 | 23.31902839 | -1.522653632 | 0.0071308 | 0.0236594 | SYTL5 | protein_coding |
| ENSG00000237886 | 4.691316307 | 16.5947185 | -1.813826803 | 0.0071316 | 0.0236594 | NALT1 | antisense |
| ENSG00000145824 | 1.056842098 | 8.833455746 | -3.101589861 | 0.0073356 | 0.0242633 | CXCL14 | protein_coding |
| ENSG00000050730 | 18.57984242 | 38.4778897 | -1.050354895 | 0.0073886 | 0.0244019 | TNIP3 | protein_coding |
| ENSG00000277383 | 8.714222057 | 1.005131762 | 3.151309981 | 0.0074215 | 0.0244821 | AC010331.1 | antisense |
| ENSG00000283537 | 1.055161684 | 8.748566901 | -3.091489062 | 0.0074494 | 0.0245646 | AC073264.3 | transcribed_unprocessed_pseudogene |
| ENSG00000174137 | 23.82517688 | 8.697208673 | 1.447150784 | 0.0075103 | 0.0247199 | FAM53A | protein_coding |
| ENSG00000184408 | 3.244917529 | 14.88664561 | -2.244825147 | 0.0075729 | 0.0249037 | KCND2 | protein_coding |
| ENSG00000234311 | 14.94872904 | 33.48636242 | -1.165224748 | 0.0078002 | 0.0255866 | AL451069.3 | antisense |
| ENSG00000226604 | 11.44231705 | 28.29577605 | -1.301676987 | 0.0078104 | 0.0256151 | PAPPA-AS2 | antisense |
| ENSG00000158445 | 16.14595153 | 4.502332257 | 1.830680241 | 0.0078883 | 0.0258208 | KCNB1 | protein_coding |
| ENSG00000244425 | 0.729358477 | 9.054591662 | -3.702762804 | 0.0079211 | 0.0259181 | RN7SL268P | misc_RNA |
| ENSG00000251442 | 18.79965529 | 38.4533658 | -1.036995263 | 0.0080612 | 0.0263054 | LINC01094 | lincRNA |
| ENSG00000125378 | 2.436126575 | 11.94985796 | -2.319527041 | 0.0082352 | 0.0268111 | BMP4 | protein_coding |
| ENSG00000227932 | 2.285663692 | 11.90792154 | -2.353067845 | 0.0084192 | 0.0273419 | SELENOOLP | transcribed_unitary_pseudogene |
| ENSG00000253882 | 3.421938268 | 14.06597854 | -2.045422113 | 0.0084832 | 0.0275233 | AC099548.2 | transcribed_unprocessed_pseudogene |
| ENSG00000258645 | 11.85259398 | 27.9940005 | -1.243410265 | 0.0085149 | 0.027605 | HSPE1P2 | processed_pseudogene |
| ENSG00000182950 | 4.43822392 | 15.47116727 | -1.810741282 | 0.0085995 | 0.0278303 | ODF3L1 | protein_coding |
| ENSG00000276718 | 2.035932134 | 10.86297796 | -2.422963726 | 0.0086672 | 0.0279861 | AC005840.4 | antisense |
| ENSG00000270681 | 18.53760556 | 6.216686348 | 1.584189931 | 0.008694 | 0.0280567 | AC095055.1 | antisense |
| ENSG00000164849 | 5.704241131 | 18.01459424 | -1.655605513 | 0.0087419 | 0.0281897 | GPR146 | protein_coding |
| ENSG00000120833 | 7.04632999 | 20.16526143 | -1.509698123 | 0.0089596 | 0.0287766 | SOCS2 | protein_coding |
| ENSG00000278997 | 2.841362259 | 13.5202527 | -2.282913072 | 0.0091632 | 0.0293471 | AL662907.1 | TEC |
| ENSG00000102387 | 22.03696712 | 7.822042782 | 1.501990431 | 0.0094813 | 0.0302458 | TAF7L | protein_coding |
| ENSG00000242574 | 2.726414579 | 13.1016746 | -2.267569423 | 0.0098742 | 0.0313222 | HLA-DMB | protein_coding |
| ENSG00000204176 | 12.17839718 | 27.84690054 | -1.193609257 | 0.0100816 | 0.0319077 | SYT15 | protein_coding |
| ENSG00000213700 | 13.84796967 | 30.77071069 | -1.147125605 | 0.0100833 | 0.0319077 | RPL17P50 | processed_pseudogene |
| ENSG00000268756 | 1.017966067 | 8.57250801 | -3.082778133 | 0.0101143 | 0.031994 | AC104534.1 | antisense |
| ENSG00000092096 | 15.08351292 | 33.15835345 | -1.133135452 | 0.0102358 | 0.0323061 | SLC22A17 | protein_coding |
| ENSG00000259347 | 13.47656877 | 3.58208934 | 1.916341786 | 0.01043 | 0.0328064 | AC087482.1 | antisense |
| ENSG00000257913 | 6.316971514 | 18.91557846 | -1.567497436 | 0.010432 | 0.0328064 | DDN-AS1 | antisense |
| ENSG00000266733 | 3.494649087 | 13.39342743 | -1.961401483 | 0.0114185 | 0.0354119 | TBC1D29 | protein_coding |
| ENSG00000203780 | 11.10003847 | 26.76316114 | -1.287216733 | 0.0115668 | 0.0358061 | FANK1 | protein_coding |
| ENSG00000279495 | 39.79578809 | 19.36343797 | 1.041006766 | 0.0115781 | 0.0358347 | AL928654.4 | TEC |
| ENSG00000284747 | 15.99716906 | 33.18731239 | -1.055373558 | 0.0116382 | 0.0360009 | AL034417.4 | antisense |
| ENSG00000276241 | 10.57985511 | 24.90798829 | -1.242241818 | 0.0117673 | 0.0363404 | AC243829.2 | antisense |
| ENSG00000260097 | 18.00430765 | 36.99681885 | -1.037992862 | 0.0119093 | 0.0367388 | SPDYE6 | protein_coding |
| ENSG00000147138 | 10.34659892 | 25.3401448 | -1.28253163 | 0.0119254 | 0.0367818 | GPR174 | protein_coding |
| ENSG00000157654 | 27.17967207 | 11.30455627 | 1.261587179 | 0.0119524 | 0.0368517 | PALM2-AKAP2 | protein_coding |
| ENSG00000249669 | 3.912999313 | 15.60214914 | -1.968861966 | 0.0120876 | 0.037221 | CARMN | lincRNA |
| ENSG00000226029 | 9.448621777 | 23.05909665 | -1.284652285 | 0.012282 | 0.037751 | LINC01772 | lincRNA |
| ENSG00000130045 | 8.467851327 | 21.4202096 | -1.339344183 | 0.0124462 | 0.0381865 | NXNL2 | protein_coding |
| ENSG00000169989 | 10.96605096 | 26.4238842 | -1.254992886 | 0.0126348 | 0.038688 | TIGD4 | protein_coding |
| ENSG00000158104 | 5.960694347 | 18.22732434 | -1.596043444 | 0.0127317 | 0.0389354 | HPD | protein_coding |
| ENSG00000171872 | 5.160860739 | 16.54650087 | -1.692728347 | 0.0129044 | 0.0393617 | KLF17 | protein_coding |
| ENSG00000272604 | 16.02764302 | 33.99925117 | -1.086832423 | 0.0129297 | 0.0394198 | AC073073.2 | antisense |
| ENSG00000144712 | 17.34261875 | 5.936479922 | 1.556726578 | 0.013074 | 0.0398025 | CAND2 | protein_coding |
| ENSG00000089820 | 9.779466227 | 24.02229199 | -1.293909984 | 0.0131912 | 0.0401233 | ARHGAP4 | protein_coding |
| ENSG00000232909 | 3.340825376 | 13.02731617 | -1.951732535 | 0.0133624 | 0.040571 | AL157823.2 | antisense |
| ENSG00000197705 | 16.70748771 | 34.53555521 | -1.03802141 | 0.0135318 | 0.04099 | KLHL14 | protein_coding |
| ENSG00000070182 | 4.070183853 | 13.86895144 | -1.772591539 | 0.0135572 | 0.0410521 | SPTB | protein_coding |
| ENSG00000149150 | 25.34316134 | 10.72529981 | 1.237297558 | 0.0135998 | 0.0411518 | SLC43A1 | protein_coding |
| ENSG00000135374 | 10.21517587 | 23.97619897 | -1.235636389 | 0.0137922 | 0.041652 | ELF5 | protein_coding |
| ENSG00000235677 | 22.38012974 | 8.739145098 | 1.354540067 | 0.0138686 | 0.0418604 | NPM1P26 | processed_pseudogene |
| ENSG00000235665 | 4.073544682 | 14.03558853 | -1.784899921 | 0.0141555 | 0.0426049 | LINC00298 | lincRNA |
| ENSG00000174586 | 13.27411531 | 29.02180999 | -1.130219299 | 0.0142353 | 0.0428299 | ZNF497 | protein_coding |
| ENSG00000176723 | 1.67293331 | 9.387587396 | -2.478752228 | 0.0143104 | 0.043048 | ZNF843 | protein_coding |
| ENSG00000100628 | 7.488761292 | 20.19394192 | -1.439244618 | 0.0145017 | 0.0435927 | ASB2 | protein_coding |
| ENSG00000169668 | 3.786617506 | 13.53424619 | -1.852965117 | 0.014597 | 0.0438636 | BCRP2 | transcribed_unprocessed_pseudogene |
| ENSG00000137699 | 11.04716385 | 25.56058719 | -1.203953746 | 0.0147019 | 0.0441395 | TRIM29 | protein_coding |
| ENSG00000224066 | 2.726414579 | 11.88552225 | -2.124437968 | 0.0150496 | 0.0450079 | AL049795.1 | antisense |
| ENSG00000128918 | 12.86719922 | 28.08059886 | -1.123710794 | 0.0154761 | 0.0460881 | ALDH1A2 | protein_coding |
| ENSG00000115165 | 5.334520649 | 16.55278207 | -1.622800526 | 0.0154798 | 0.0460909 | CYTIP | protein_coding |
| ENSG00000236366 | 2.327900552 | 10.67080098 | -2.182867897 | 0.0155161 | 0.0461667 | AL359313.1 | lincRNA |
| ENSG00000128833 | 4.319915411 | 14.74411733 | -1.76152979 | 0.0155223 | 0.046177 | MYO5C | protein_coding |
| ENSG00000272438 | 1.458716954 | 9.004664514 | -2.69360166 | 0.0156125 | 0.0463881 | AL645608.6 | lincRNA |
| ENSG00000279118 | 11.38192441 | 26.29391833 | -1.219699237 | 0.0156152 | 0.0463881 | AC093535.2 | TEC |
| ENSG00000237575 | 3.057259029 | 11.92574915 | -1.968950611 | 0.0157016 | 0.0466039 | PYY2 | transcribed_unprocessed_pseudogene |
| ENSG00000204776 | 16.57102342 | 5.848450476 | 1.504048272 | 0.0157925 | 0.046841 | IGKV1OR-3 | IG_V_pseudogene |
| ENSG00000115109 | 11.08435947 | 25.08718778 | -1.171516528 | 0.0158375 | 0.0469415 | EPB41L5 | protein_coding |
| ENSG00000130477 | 13.41057961 | 28.96347703 | -1.107847099 | 0.0164938 | 0.0485925 | UNC13A | protein_coding |
| ENSG00000261474 | 12.53299394 | 28.08373946 | -1.162252878 | 0.0165406 | 0.0487184 | AC026471.4 | lincRNA |
